# Synthesis of *N*-perfluoroalkyl-3,4-disubstituted pyrroles by rhodium-catalyzed transannulation of *N*-fluoroalkyl-1,2,3-triazoles with terminal alkynes

**DOI:** 10.3762/bjoc.17.44

**Published:** 2021-02-18

**Authors:** Olga Bakhanovich, Viktor Khutorianskyi, Vladimir Motornov, Petr Beier

**Affiliations:** 1 Institute of Organic Chemistry and Biochemistry of the Czech Academy of Sciences, Flemingovo náměstí 2, 166 10 Prague 6, Czech Republic; 2 Department of Organic Chemistry, Faculty of Science, Charles University, Hlavova 2030/8, 128 43 Prague, Czech Republic

**Keywords:** pyrrole, transannulation, rhodium carbene, triazole

## Abstract

The rhodium-catalyzed transannulation of *N-*perfluoroalkyl-1,2,3-triazoles with aromatic and aliphatic terminal alkynes under microwave heating conditions afforded *N-*perfluoroalkyl-3,4-disubstituted pyrroles (major products) and *N-*fluoroalkyl-2,4-disubstituted pyrroles (minor products). The observed selectivities in the case of the reactions with aliphatic alkynes were high.

## Introduction

**Figure 1:**
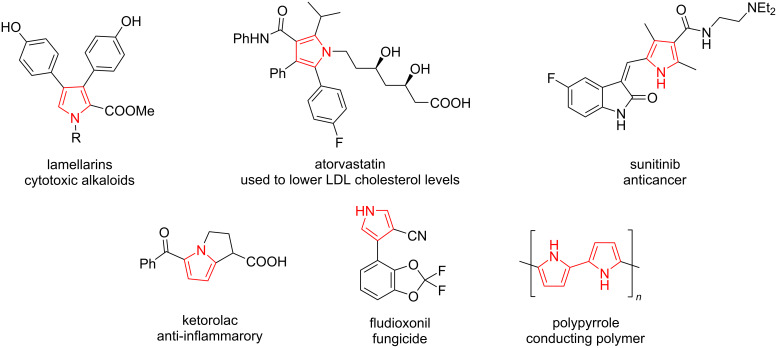
Selected pyrrole-containing natural products, drugs, agrochemicals, and functional materials.

Pyrroles are known to be important structural moieties appearing in natural products, synthetic drugs, agrochemicals, and functional materials (polymers, dyes, films, etc.) (Figure 1) [1–4]. Numerous methods exist for pyrrole synthesis, including the classical and industrially important condensation approaches, such as the Hantzsch, Huisgen, and Paal–Knorr processes [5]. However, the direct modification of pyrroles to the 3,4-disubstituted derivatives is challenging because an electrophilic aromatic substitution of pyrroles or the metalation of *N-*substituted pyrroles and the subsequent reaction with electrophiles take place in position two of the ring [6–7].

**Scheme 1:**
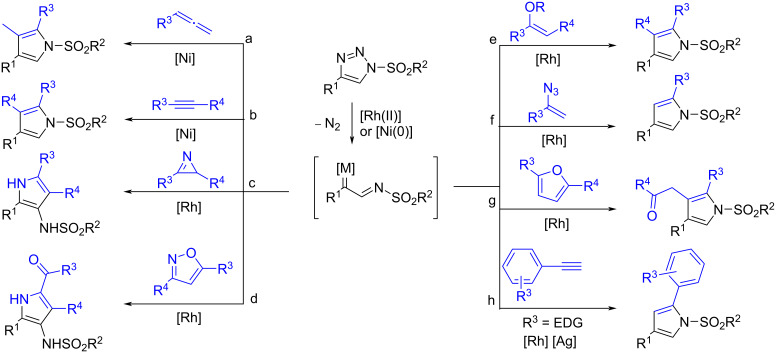
Transformation of *N-*sulfonyl-1,2,3-triazoles to pyrroles via metal iminocarbenes.

Recently, *N-*sulfonyl-1,2,3-triazoles, conveniently prepared by [3 + 2] cycloadditions of terminal alkynes with sulfonyl azides, have been used as the precursors to *N-*sulfonylindoles by transition*-*metal-catalyzed transannulation reactions. In the presence of Rh(II) or Ni(0) catalysts the triazole ring-opening takes place and intermediate highly electrophilic metal-bound iminocarbenes form. These iminocarbenes undergo a variety of intriguing reactions, such as a cycloaddition and a C–H functionalization, among others, leading mostly to nitrogen heterocycles [8–10]. Using this chemistry, a variety of pyrroles have been prepared starting from *N-*sulfonyl-1,2,3-triazoles (Scheme 1) [11–17].

We have recently reported that *N-*perfluoroalkyl-1,2,3-triazoles [18] undergo rhodium-catalyzed transannulation reactions leading to various nitrogen heterocycles, such as imidazoles, pyrrolones, imidazolones, oxazoles, azepines [19–22], and pyrroles [19].

The use of fluorine atoms and fluoro groups (including the trifluoromethyl group) is a widely used strategy to improve the properties of drug candidates or agrochemicals [23–28]. The development of new methods for the synthesis of selectively fluorinated and trifluorometylated compounds is essential for future progress in areas that eventually improve the quality of life. In this context, *N-*trifluoromethylated compounds (amines, amides and nitrogen heterocycles) are a relatively underexplored group of molecules with a high potential in medicinal chemistry [29–30]. Taking inspiration from the work of Gevorgyan (Scheme 1h) [11], we report herein our recent results on the rhodium-catalyzed transannulation of *N-*perfluoroalkyl-1,2,3-triazoles with terminal alkynes leading to unusually substituted *N-*perfluoroalkylpyrroles.

## Results and Discussion

The published transannulation of *N-*tosyl-1,2,3-triazoles with terminal alkynes requires the use of a Rh/Ag binary catalyst system, works only with electron*-*rich arylacetylenes and leads to *N-*tosyl-2,4-disubstituted pyrroles (Scheme 1h) [11]. The application of these conditions to *N-*trifluoromethyltriazole **1a** and phenylacetylene leads to a mixture of 3,4-diphenylpyrrole **2a** and 2,4-diphenylpyrrole **2a’**. However, repeating the experiment without the silver catalyst afforded the same results showing that the silver catalyst was not necessary in our case and that the product regioselectivity was not dependent on the silver catalyst (Table 1, entry 1). Chloroform was found to be the most suitable solvent and varying rhodium catalysts led to the **2a** + **2a’** product mixture of various ratios. Rh_2_(oct)_4_ and Rh_2_(esp)_2_ gave the highest **2a**/**2a’** ratio, while the electron-deficient Rh_2_(pfb)_4_ catalyst gave the lowest **2a**/**2a’** ratio and an incomplete conversion. Reducing the reaction temperature to 80 °C afforded a full conversion of the starting triazole, but no reaction took place at 60 °C. The optimized conditions are presented in Table 1, entry 8; however, for the following reaction scope study, a temperature of 100 °C and 20 min reaction time were used to ensure a full conversion for all studied substrates.

**Table 1:** Reaction conditions screening of the transannulation of triazole **1a** with phenylacetylene.

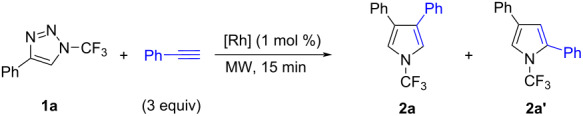					

Entry	[Rh]	Solvent	Temp. (^°^C)	Conv. (%)^a^	**2a**/**2a’**

1^b^	Rh_2_(oct)_4_	cyclohexane	120	90	75:25
2	Rh_2_(oct)_4_	CHCl_3_	120	100	60:40
3	Rh_2_(OAc)_4_	CHCl_3_	120	100	34:66
4	Rh_2_(esp)_2_	CHCl_3_	120	100	75:25
5	Rh_2_(pfb)_4_	CHCl_3_	120	54	14:86
6	Rh_2_(esp)_2_	CHCl_3_	100	100	75:25
7	Rh_2_(esp)_2_	DCE	100	100	40:60
8	Rh_2_(esp)_2_	CHCl_3_	80	100	75:25
9	Rh_2_(esp)_2_	CHCl_3_	60	NR	–

NR: no reaction. oct: *n*-C_7_H_15_COO. esp: α,α,α′,α′-tetramethyl-1,3-benzenedipropionate. pfb: *n*-C_3_F_7_COO. ^a^Conversion of **1a** was determined by ^19^F NMR spectroscopy. ^b^The same result was observed in the presence of CF_3_COOAg (5 mol %) in addition to Rh_2_(oct)_4_ (1 mol %).

**Scheme 2:**
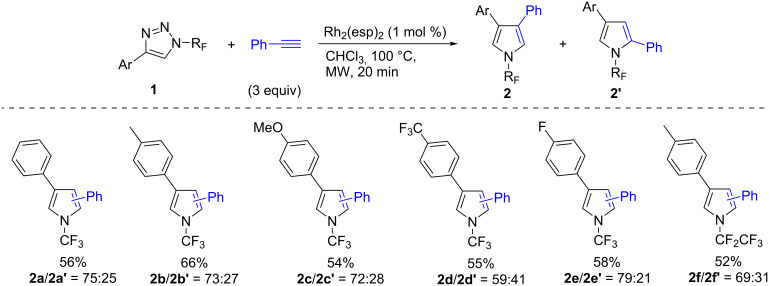
Transannulation of triazoles **2** with phenylacetylene.

Next, the transannulation reaction was tested using a range of different substituted *N-*trifluoromethyl- and *N-*pentafluoroethyl-4-aryl-1,2,3-triazoles **1** with phenylacetylene (Scheme 2). Good to moderate yields of product mixtures **2** and **2’** were obtained and the **2**/**2’** ratio ranged from 59:41 to 79:21.

**Scheme 3:**
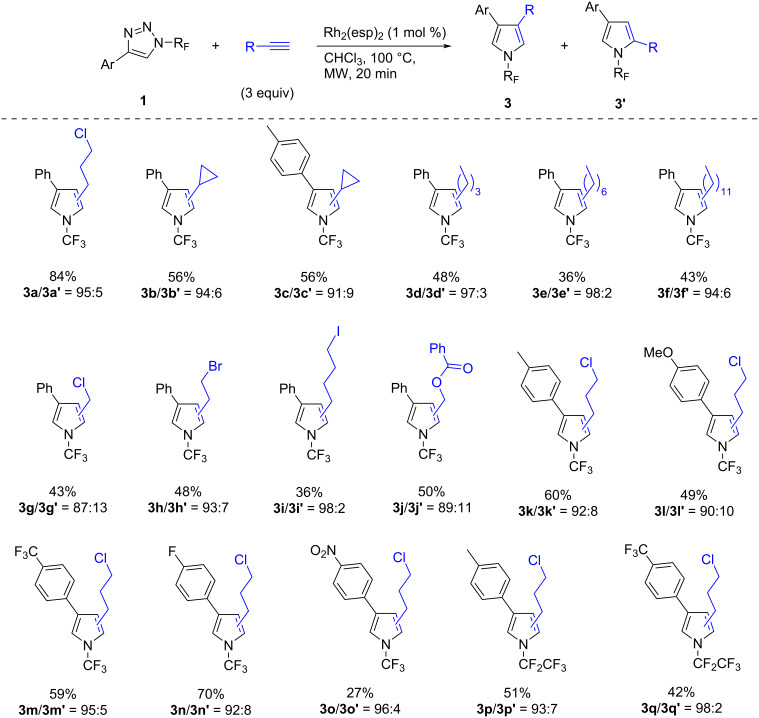
Transannulation of *N-*perfluoroalkyl-1,2,3-triazoles with aliphatic alkynes.

While aliphatic alkynes were found to be ineffective in transannulations with *N-*tosyl-1,2,3-triazoles, the reactions of *N-*perfluoroalkyl-1,2,3-triazoles with aliphatic alkynes proceeded well and the pyrroles **3** were formed in unexpectedly high selectivities, ranging from 87:13 to 98:2 (Scheme 3). The isolated product yields were moderate to good and the products were generally obtained as mixtures of regioisomers. Column chromatography allowed the separation of pure isomers of **2a**, **2a’**, **3a**, **3b**, **3d**, **3h**, **3l**, **3m**, and **3n**. However, no general trend in the efficiency of the reaction or product selectivity was observed.

**Scheme 4:**
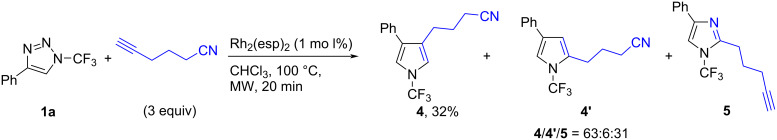
Reaction of **1a** with hex-5-ynenitrile.

Hex-5-ynenitrile was used in the transannulation with **1a** with the aim to assess the relative propensity of nitrile and alkyne groups in the reaction. The triple bond reacted in the transannulation about two times faster than the nitrile group and again the 3,4-disubstituted pyrrole **4** regioisomer dominated over the 2,4-disubstitued pyrrole **4’** (Scheme 4).

**Scheme 5:**
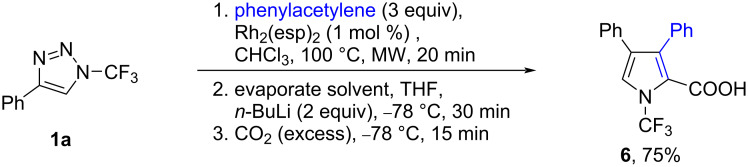
Metalation and carboxylation of in situ-prepared pyrrole **2a**.

To demonstrate the compatibility of the formed *N-*perfluoroalkylpyrroles with the conditions of pyrrole derivatization by metalation in position two and reaction with an electrophile, the crude product **2a** was treated with butyllithium, followed by the reaction with carbon dioxide to afford pyrrole carboxylic acid **6** in a good overall yield (Scheme 5). The trifluoromethyl group on the nitrogen was not affected by these highly basic reaction conditions.

**Scheme 6:**
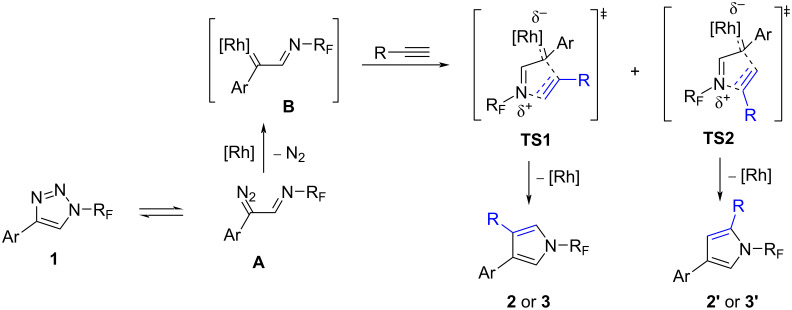
Plausible mechanism for rhodium-catalyzed transannulation of *N-*perfluoroalkyl-1,2,3-triazoles with alkynes.

The mechanism of the rhodium-catalyzed transannulation to pyrroles has recently been investigated computationally with *N-*sulfonyltriazoles [31]. It seems that the formed rhodium carbenoid **B** reacts with the alkyne in a concerted process and even in the presence of Ag^+^ salts, a nucleophilic addition of silver acetylides does not take place. In our case, the transition states **TS1** and **TS2** have roughly similar energies for phenylacetylene and **TS1** is lower in energy for aliphatic alkynes (Scheme 6).

## Conclusion

In conclusion, the rhodium-catalyzed transannulation of *N-*perfluoroalkyl-1,2,3-triazoles with terminal alkynes was described. The reaction led to a mixture of the *N-*perfluoroalkyl-3,4- and 2,4-disubstituted pyrroles. The reactions with phenylacetylene afforded a mixture of 3,4- and 2,4-disubstituted pyrroles in a ratio from 59:41 to 79:21, while the reactions with aliphatic acetylenes gave higher product regioselectivities (87:13 to 98:2). This is the first report of a transannulation leading to 3,4-disubstituted pyrroles. Additionally, the method did not require the use of a silver(I) co-catalyst. The scope for aliphatic alkynes is reasonably wide and the isolated yields were moderate to good. A one-pot transannulation/carboxylation process was demonstrated for the construction of the functionalized pyrrole 2-carboxylic acid with an *N-*trifluoromethyl functionality. Thus, this work improves the synthetic access to *N-*perfluoroalkyl-3,4-disubstituted pyrroles.

## Supporting Information

File 1 Experimental part.
