# Supplementary material for: Synthesis of N-perfluoroalkyl-3,4-disubstituted pyrroles by rhodium-catalyzed transannulation of N-fluoroalkyl-1,2,3-triazoles with terminal alkynes
Source: Beilstein J Org Chem. 2021 Feb 18;17:504–10. doi: 10.3762/bjoc.17.44 (PMC7934709; doi:10.3762/bjoc.17.44)

BEILSTEIN JOURNAL OF ORGANIC CHEMISTRY

Supporting Information

for

Synthesis of *N*-perfluoroalkyl-3,4-disubstituted pyrroles by rhodium-catalyzed transannulation of *N*-fluoroalkyl-1,2,3-triazoles with terminal alkynes

Olga Bakhanovich, Viktor Khutorianskyi, Vladimir Motornov and Petr Beier

Beilstein J. Org. Chem. **2021,** *17,* 504–510. doi:10.3762/bjoc.17.44

Experimental part

License and Terms: This is a supporting information file under the terms of the Creative Commons Attribution License (https://creativecommons.org/licenses/by/4.0). Please note that the reuse, redistribution and reproduction in particular requires that the author(s) and source are credited and that individual graphics may be subject to special legal provisions.
The license is subject to the *Beilstein Journal of Organic Chemistry* terms and conditions: (https://www.beilstein-journals.org/bjoc/terms)

General

Chloroform stabilized with ethanol (≈ 1%) was distilled over sulfuric acid and stored under argon. All commercially available chemicals were used as received unless stated otherwise. The starting triazoles **1a–i** were prepared according to procedures published in the literature [1]. Flash column chromatography was performed using silica gel 60 (0.040–0.063 mm) and pure pentane as the mobile phase or C18 silica gel using a MeCN/H_2O mixture as the mobile phase. Automated flash column chromatography was performed on Teledyne ISCO CombiFlash Rf+ Lumen Automated Flash Chromatography System with UV–vis detection. ^{1}H, ^{13}C, and ^{19}F NMR spectra were measured at ambient temperature using 5 mm diameter NMR tubes. ^{13}C NMR spectra were proton decoupled. The chemical shift values (δ) are reported in ppm relative to internal Me_4Si (0 ppm for ^{1}H and ^{13}C NMR) or residual solvents and internal $CFCl_3$ (0 ppm for ^{19}F NMR). Coupling constants (*J*) are reported in hertz. The structural elucidation was aided by the additional acquisition of ^{13}C APT and/or various 2D spectra (^{1}H-^{1}H COSY, ^{1}H-^{13}C HSQC, ^{1}H-^{13}C HMBC, ^{13}C-^{19}F HMBC). High resolution MS spectra (HRMS) were recorded on a Waters Micromass AutoSpec Ultima or an Agilent 7890A GC coupled with a Waters GCT Premier orthogonal acceleration time-of-flight detector using electron impact (EI) ionization or chemical ionization (CI), Q-Tof micro (Waters) is a quadrupole orthogonal acceleration time-of-flight tandem mass spectrometer using atmospheric-pressure chemical ionization (APCI). A CEM Discover System (150 W power) was used for reactions carried out in a microwave reactor.

General procedure for reactions of triazoles and alkynes

The *N*-(per)fluoroalkyl-triazole **1a–i** (0.20 mmol) was dissolved in dry $CHCl_3$ (2 mL) in a 10 mL microwave tube. The alkyne (3 equiv, 0.60 mmol) and bis[rhodium(α,α,α′,α′-tetramethyl-1,3-benzenedipropionic acid)] (0.002 mmol; 1.52 mg) were added, the vial capped and heated at 100 °C for 20 min in a microwave reactor. The solvent and unreacted alkyne were removed under reduced pressure and the product was purified by CombiFlash automatic column chromatography (silica gel using pentane as the mobile phase – general procedure A or reversed phase chromatography using water/MeCN as the mobile phase – general procedure B).

3,4-Diphenyl-1-(trifluoromethyl)-1H-pyrrole and *2,4-diphenyl-1-(trifluoromethyl)-1H-*

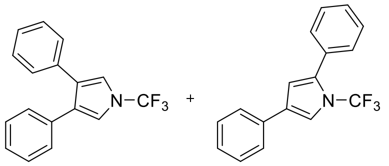

*pyrrole (***2a** + **2a'***)*: Prepared according to the **general procedure A** with phenylacetylene as the starting alkyne. Yield: 56%; colourless oil; crude ^{19}F NMR ratio: 75:25; ^{1}H NMR (401 MHz, $CDCl_3$) δ 7.60 – 7.53 (m, 2H, **2a'**), 7.53 – 7.48 (m, 2H, **2a'**), 7.47 – 7.43 (m, 4H, **2a'**), 7.43 – 7.41 (m, 1H, **2a'**), 7.41 – 7.39 (m, 1H, **2a'**), 7.36 (dq, *J* = 1.7, 0.9 Hz, 1H, **2a'**), 7.35 – 7.23 (m, 10H, **2a**), 7.11 (s, 2H, **2a'**), 6.61 (dq, *J* = 1.7, 0.8 Hz, 1H, **2a'**); ^{13}C NMR (101 MHz, $CDCl_3$) δ 134.7, 133.9, 133.8, 132.6, 131.7, 129.7, 129.0, 128.7, 128.6, 128.5, 128.3, 127.3, 127.0, 127.0, 125.7, 119.1 (q, $^{1}J_{C\text{-}F}$ = 260.5 Hz), 116.4, 115.4, 112.4; ^{19}F NMR (377 MHz, $CDCl_3$) δ -52.18 (s, **2a'**), -57.67 (s, **2a**); HRMS (EI^+) *m/z* calcd for $C_{17}H_{12}F_3N$ $[M]^+$: 287.0922, found 287.0920.

*3,4-Diphenyl-1-(trifluoromethyl)-1H-pyrrole (***2a***)*: white crystalline solid; ^{1}H NMR (401 MHz, $CDCl_3$) δ 7.24 – 7.11 (m, 10H), 7.01 (s, 2H); ^{13}C NMR (101 MHz, $CDCl_3$) δ 133.9, 128.7, 128.5, 127.3, 127.0, 119.1 (q, $^{1}J_{C\text{-}F}$ = 260.7 Hz), 116.4; ^{19}F NMR (377 MHz, $CDCl_3$) δ -57.68 (s).

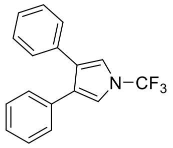

*2,4-Diphenyl-1-(trifluoromethyl)-1H-pyrrole (***2a'***)*: colourless oil; ^{1}H NMR (401 MHz, $CDCl_3$) δ 7.57 – 7.52 (m, 2H), 7.51 – 7.46 (m, 2H), 7.45 – 7.36 (m, 4H), 7.34 (dd, *J* = 2.0, 0.9 Hz, 1H), 7.30 – 7.27 (m, 1H), 6.59 (dd, *J* = 2.0, 1.0 Hz, 1H); ^{13}C NMR (101 MHz, $CDCl_3$) δ 134.7, 133.8, 131.7, 129.7, 129.7, 129.0, 128.6, 128.3, 127.0, 126.8, 126.8, 125.7, 119.6 (q, $^{1}J_{C\text{-}F}$ = 262.6 Hz), 115.41, 115.39, 115.37, 115.35, 112.39, 112.38; ^{19}F NMR (377 MHz, $CDCl_3$) δ -52.18 (s).

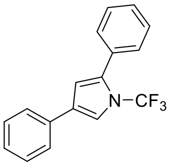

3-Phenyl-4-(p-tolyl)-1-(trifluoromethyl)-1H-pyrrole in mixture with *2-phenyl-4-(p-tolyl)-1-(trifluoromethyl)-1H-pyrrole (***2b** + **2b'***)*: Prepared according to the **general procedure A** with phenylacetylene as the starting alkyne. Yield: 66%; colourless oil; crude ^{19}F NMR ratio: 73:27; ^{1}H NMR (401 MHz, $CDCl_3$) δ 7.55 – 7.05 (m, 11H + 10H', **2b** + **2b'**), 6.58 (dd, *J* = 1.9, 0.9 Hz, 1H, **2b'**), 2.39 (s, 3H, **2b'**), 2.36 (s, 3H, **2b**); ^{13}C NMR (101 MHz, $CDCl_3$) δ 136.7, 136.7, 134.6, 134.0, 131.8, 130.9, 129.6, 129.2, 128.7, 128.6, 128.5, 128.4, 128.3, 127.2, 126.9, 126.8, 125.6, 119.4 (q, $^{1}J_{C\text{-}F}$ = 262.3 Hz), 119.1 (q, $^{1}J_{C\text{-}F}$ = 260.4 Hz), 116.4, 116.2, 115.1, 115.0, 112.4, 112.4, 21.3; ^{19}F NMR (377 MHz, $CDCl_3$) δ -51.61 (s, **2b'**), -57.14 (s, **2b**); HRMS (EI^{+}) *m/z* calcd for $C_{18}H_{14}F_3N$ $[M]^{+}$: 301.1078, found 301.1076.

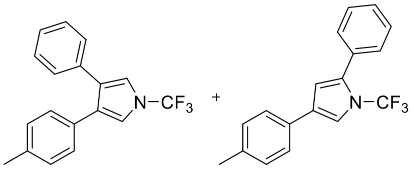

3-(4-Methoxyphenyl)-4-phenyl-1-(trifluoromethyl)-1H-pyrrole and *4-(4-methoxyphenyl)-2-phenyl-1-(trifluoromethyl)-1H-pyrrole* (**2c** + **2c'**):

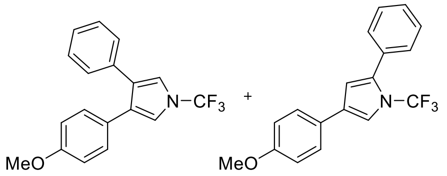

Prepared according to the **general procedure A** with phenylacetylene as the starting alkyne. Yield: 54%; colourless oil; crude ^{19}F NMR ratio: 72:28; ^{1}H NMR (401 MHz, $CDCl_3$) δ 7.55 – 7.24 (m, 5H + 9H', **2c** + **2c'**), 7.23 – 7.14 (m, 2H, **2c**), 7.09 (dd, J = 15.5, 2.5 Hz, 2H, **2c**), 7.01 – 6.92 (m, 2H, **2c'**), 6.91 – 6.80 (m, 2H, **2c**), 6.56 (dq, *J* = 1.7, 0.8 Hz, 1H, **2c'**), 3.87 (s, 3H, **2c'**), 3.84 (s, 3H, **2c**); ^{13}C NMR (101 MHz, $CDCl_3$) δ 158.8, 134.0, 129.9, 129.6, 128.7, 128.5, 128.5, 128.3, 127.2, 126.9, 126.8, 126.3, 119.4 (q, $^{1}J_{C\text{-}F}$ = 262.1 Hz), 119.1 (q, $^{1}J_{C\text{-}F}$ = 260.6 Hz), 117.8, 116.3, 119.0, 114.4, 113.9, 112.4, 55.4, 55.4; ^{19}F NMR (377 MHz, $CDCl_3$) δ -52.11 (s, **2c'**), -57.65 (s, **2c**); HRMS (EI^+) *m/z* calcd for $C_{18}H_{14}F_3NO$ $[M]^+$: 317.1027, found 317.1026.

3-Phenyl-1-(trifluoromethyl)-4-(4-(trifluoromethyl)phenyl)-1H-pyrrole with *2-phenyl-1-(trifluoromethyl)-4-(4-(trifluoromethyl)phenyl)-1H-pyrrole* (**2d** + **2d'**):

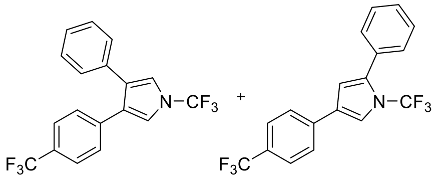

Prepared according to the **general procedure A** with phenylacetylene as the starting alkyne. Yield: 55%; colourless oil; crude ^{19}F NMR ratio: 59:41; ^{1}H NMR (401 MHz, $CDCl_3$) δ 7.64 (s, 4H, **2d'**), 7.55 – 7.51 (m, 2H, **2d**), 7.50 – 7.46 (m, 2H, **2d'**), 7.46 – 7.42 (m, 1H, **2d**), 7.42 – 7.39 (m, 1H, **2d'**), 7.36 – 7.28 (m, 4H + 3H', **2d** + **2d'**), 7.24 – 7.20 (m, 2H, **2d**), 7.13 (dd, *J* = 16.3, 2.5 Hz, 2H, **2d**), 6.60 (dd, *J* = 2.0, 0.9 Hz, 1H, **2d'**); ^{13}C NMR (126 MHz, $CDCl_3$) δ 136.4, 136.2, 134.0, 132.2, 130.1, 128.7, 128.6, 128.6, 128.5, 128.5, 128.4, 128.3, 128.2, 128.1, 128.0, 128.0, 127.9, 127.9, 127.9, 127.9, 127.8, 127.8, 127.8,

127.7, 127.6, 127.6, 127.6, 127.6, 127.5, 127.5, 127.4, 127.3, 127.2, 126.9, 126.9, 126.9, 126.9, 126.8, 126.5, 126.1, 126.1, 124.9, 124.8, 124.8, 124.8, 124.7, 124.7, 124.6, 124.6, 124.5, 124.4, 124.3, 124.3, 124.3, 124.2, 124.2, 124.1, 124.0, 123.7, 123.5, 123.2 (q, *J* = 271.7 Hz, **2d'**), 123.2 (q, *J* = 272.1 Hz, **2d**), 123.0, 118.1, 118.1, 118.0 (q, *J* = 263.0 Hz, **2d'**), 117.8 (q, *J* = 261.0 Hz, **2d**), 115.8, 115.7, 115.0, 115.0, 115.0, 115.0, 114.9, 114.4, 110.9; ^{19}F NMR (377 MHz, $CDCl_3$) δ -52.33 (s, *N*-CF_3, **2d'**), -57.76 (s, *N*-CF_3, **2d**), -62.93 (s, **2d'**), -62.94 (s, **2d**); HRMS (APCI$^+$) *m/z* calcd for C18H1F6N [M + H]$^+$: 356.08685, found 356.08675.

3-(4-Fluorophenyl)-4-phenyl-1-(trifluoromethyl)-1H-pyrrole and *4-(4-fluorophenyl)-2-phenyl-1-(trifluoromethyl)-1H-pyrrole* (**2e** + **2e'**): Prepared according to the **general procedure A** with phenylacetylene as the starting alkyne. Yield: 58%; colourless oil; crude ^{19}F NMR ratio: 79:21; ^{1}H NMR (401 MHz, $CDCl_3$) δ 7.57 – 7.39 (m, 7H, **2e'**), 7.38 – 7.27 (m, 3H + 1H', **2e** + **2e'**), 7.25 – 7.16 (m, 4H, **2e**), 7.14 – 7.06 (m, 2H + 2H', **2e** + **2e'**), 7.04 – 6.95 (m, 2H, **2e**), 6.55 (dq, *J* = 1.6, 0.8 Hz, 1H, **2e'**); ^{13}C NMR (101 MHz, $CDCl_3$) δ 162.1 (d, *J* = 245.8 Hz), 162.1 (d, *J* = 245.6 Hz), 134.7, 133.6, 131.5, 131.1, 130.3, 130.2, 130.0, 129.9, 129.9, 129.9, 129.8, 129.8, 129.6, 129.6, 129.5, 129.5, 129.2, 129.1, 128.9, 128.6, 128.5, 128.5, 128.5, 128.2, 128.0, 127.9, 127.5, 127.2, 127.1, 127.0, 127.0, 126.6, 126.6, 126.2, 125.8, 125.4, 122.8, 119.4 (q, *J* = 262.7 Hz), 119.1 (q, *J* = 260.8 Hz), 116.3, 116.2, 115.8, 115.8, 115.6, 115.4, 115.2, 115.0, 112.2, 112.2.; ^{19}F NMR (377 MHz, $CDCl_3$) δ -51.69 (s, *N*-CF_3, **2e'**), -57.18 (s, *N*-CF_3, **2e**), -115.61 to -115.73 (m, **2e**, **2e'**); HRMS (APCI$^+$) *m/z* calcd for $C_{17}H_{11}F_4N$ [M + H]$^+$: 306.09004, found 306.08998.

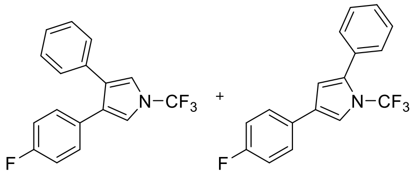

1-(Perfluoroethyl)-3-phenyl-4-(p-tolyl)-1H-pyrrole with *1-(perfluoroethyl)-2-phenyl-4-(p-tolyl)-1H-pyrrole (***2f** + **2f’***)*: Prepared according to the **general procedure A** with phenylacetylene as the starting alkyne. Yield: 52%; colourless oil; crude ^{19}F NMR ratio: 69:31; ^{1}H NMR (401 MHz, $CDCl_3$) δ 7.56 – 7.22 (m, 5H + 8H’, **2f’** + **2f’**), 7.21 – 7.12 (m, 4H + 1H’, **2f’** + **2f’**), 7.10 – 7.02 (m, 2H + 1H’, **2f’** + **2f’**), 6.60 (dt, *J* = 2.0, 1.0 Hz, 1H, **2f’**), 2.42 (s, 3H, **2f’**), 2.39 (s, 3H, **2f’**); ^{13}C NMR (101 MHz, $CDCl_3$) δ 140.11, 139.56, 139.35, 139.16, 136.83, 136.70, 135.91, 135.11, 135.05, 133.98, 132.48, 132.30, 132.09, 131.22, 130.88, 130.78, 130.39, 130.31, 129.79, 129.65, 129.36, 129.22, 128.88, 128.84, 128.74, 128.61, 128.49, 128.46, 128.27, 128.19, 128.15, 128.09, 128.03, 127.92, 127.88, 127.81, 127.75, 127.69, 127.51, 127.49, 127.48, 127.24, 126.96, 125.58, 124.14, 124.02, 123.98, 123.19, 123.01, 122.41, 122.32, 122.13, 121.83, 120.71, 119.74, 119.28, 118.81, 116.89, 116.75, 115.80, 113.58, 113.29, 112.02, 111.60, 111.08, 110.67, 109.01, 108.68, 108.46, 108.29, 107.96, 21.30; ^{19}F NMR (377 MHz, $CDCl_3$) δ -84.75 (s, $-CF_3$, **2f’**), -85.70 (s, $-CF_3$, **2f**), -93.82 (s, *N*-CF_2, **2f’**), -99.11 (s, *N*-CF_2, **2f**); HRMS (EI$^+$) *m/z* calcd for $C_{19}H_{14}F_5N$ $[M]^+$: 351.1046, found 351.1045.

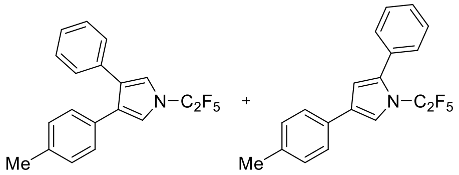

3-Chloropropyl-4-phenyl-1-(trifluoromethyl)-1H-pyrrole and 2-chloropropyl-4-phenyl-1-(trifluoromethyl)-1H-pyrrole (**3a** + **3a’**): Prepared according to the **general procedure A** with 1-chloro-pent-4-yne the as starting alkyne. Yield: 84%; colourless oil; crude ^{19}F NMR ratio: 95:5; ^{1}H NMR (401 MHz, $CDCl_3$) δ 7.51 – 7.47 (m, 2H, **3a’**),

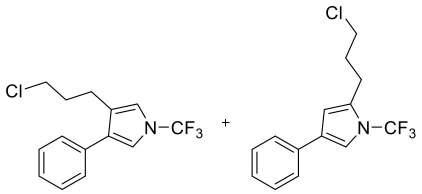

7.44 – 7.36 (m, 4H + 2H', **3a** + **3a'**), 7.35 – 7.29 (m, 1H, **3a**), 7.27 – 7.22 (m, 1H, **3a'**), 7.21 – 7.18 (m, 1H, **3a'**), 7.00 (d, *J* = 2.5 Hz, 1H, **3a**), 6.86 (dd, *J* = 2.3, 1.1 Hz, 1H, **3a**), 6.41 (dq, *J* = 1.9, 0.9 Hz, 1H, **3a'**), 3.65 (t, *J* = 6.4 Hz, 2H, **3a'**), 3.51 (ddt, *J* = 6.4 Hz, 2H, **3a**), 2.91 (td, *J* = 7.6, 2.1 Hz, 2H, **3a'**), 2.75 (td, *J* = 7.5, 1.0 Hz, 2H, **3a**), 2.24 – 2.12 (m, 2H, **3a'**), 1.95 (ddt, *J* = 8.7, 7.5, 6.4 Hz, 2H, **3a**); ^{13}C NMR (101 MHz, $CDCl_3$) δ 134.4, 133.9, 132.6, 132.6, 128.9, 128.7, 128.7, 128.6, 128.3, 127.1, 126.9, 126.4, 125.5, 124.5, 119.1 (q, $^{1}J_{C\text{-}F}$ = 260.0 Hz), 115.9, 114.3, 110.0, 44.5, 44.1, 32.4, 31.4, 22.9; ^{19}F NMR (377 MHz, $CDCl_3$) δ -55.00 (s, **4a'**), -57.55 (s, **4a**); HRMS (EI^+) *m/z* calcd for $C_{14}H_{13}ClF_3N$ $[M]^+$: 287.0689, found 287.0687.

*3-(3-Chloropropyl)-4-phenyl-1-(trifluoromethyl)-1H-pyrrole (***3a***)*: Prepared according to

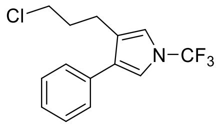

the **general procedure A** with 1-chloro-pent-4-yne the as starting alkyne. Colourless oil; ^{1}H NMR (400 MHz, $CDCl_3$) δ 7.44 – 7.33 (m, 4H), 7.36 – 7.27 (m, 1H), 6.99 (d, *J* = 2.5 Hz, 1H), 6.85 (dd, *J* = 2.4, 1.2 Hz, 1H), 3.50 (t, *J* = 6.4 Hz, 2H), 2.78 – 2.70 (m, 2H), 2.00 – 1.89 (m, 2H); ^{13}C NMR (101 MHz, $CDCl_3$) δ 134.4, 128.8, 128.3, 127.1, 124.6, 119.1 (q, $^{1}J_{C\text{-}F}$ = 260.1 Hz), 115.9, 44.5, 32.5, 22.9; ^{19}F NMR (376 MHz, $CDCl_3$) δ -57.56 (s).

3-Cyclopropyl-4-phenyl-1-(trifluoromethyl)-1H-pyrrole and 2-cyclopropyl-4-phenyl-1-

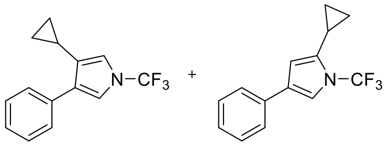

(trifluoromethyl)-1H-pyrrole (**3b** + **3b'**): Prepared according to the **general procedure A** with cyclopropylacetylene as the starting alkyne. Yield: 56%; colourless oil; crude ^{19}F NMR ratio: 94:6; ^{1}H NMR (401 MHz, $CDCl_3$) δ 7.67 – 7.58 (m, 2H, **3b**), 7.57 – 7.50 (m, 1H, **3b'**), 7.45 – 7.28 (m, 3H +

3H’, **3b** + **3b’**), 7.26 – 7.21 (m, 1H, **3b’**), 7.17 (dt, *J* = 2.1, 0.8 Hz, 1H, **3b’**), 7.03 (d, *J* = 2.5 Hz, 1H, **3b**), 6.67 (dd, *J* = 2.5, 1.0 Hz, 1H, **3b**), 6.24 (dt, *J* = 1.9, 1.0 Hz, 1H, **3b’**), 1.90 (ttd, *J* = 8.6, 6.8, 4.5 Hz, 1H, **3b’**), 1.75 (ttd, *J* = 8.3, 5.2, 1.0 Hz, 1H, **3b**), 0.97 – 0.82 (m, 2H + 2H’, **3b** + **3b’**), 0.78 – 0.71 (m, 2H, **3b’**), 0.64 – 0.56 (m, 2H, **3b**); ^{13}C NMR (101 MHz, $CDCl_3$) δ 136.6, 134.5, 134.1, 132.7, 129.4, 128.9, 128.9, 128.7, 128.6, 128.6, 128.4, 128.3, 128.1, 126.8, 126.7, 125.9 125.5, 125.1, 119.1 (q, *J* = 259.9 Hz), 115.4, 114.4, 114.2, 114.2, 108.2, 8.0, 7.5, 6.7; ^{19}F NMR (377 MHz, $CDCl_3$) δ -55.31 (s, **3b’**), -57.59 (s, **3b**); HRMS (EI$^+$) *m/z* calcd for $C_{14}H_{12}F_3N$ [M]$^+$: 251.0922, found 251.0923.

*3-Cyclopropyl-4-phenyl-1-(trifluoromethyl)-1H-pyrrole (**3b**)*: ^{1}H NMR (400 MHz, $CDCl_3$) δ

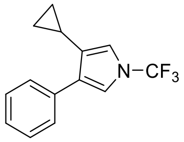

7.63 – 7.57 (m, 2H), 7.43 – 7.37 (m, 2H), 7.33 – 7.27 (m, 1H), 7.02 (d, *J* = 2.5 Hz, 1H), 6.66 (dd, *J* = 2.5, 1.0 Hz, 1H), 1.74 (ttd, *J* = 8.3, 5.2, 1.0 Hz, 1H), 0.93 – 0.82 (m, 2H), 0.64 – 0.52 (m, 2H); ^{13}C NMR (101 MHz, $CDCl_3$) δ 134.5, 128.9, 128.7, 128.6, 128.2, 128.1, 126.8, 125.5, 119.1 (q, *J* = 259.8 Hz); 115.4, 114.4, 8.0, 7.5, 6.7; ^{19}F NMR (376 MHz, $CDCl_3$) δ -57.61 (s).

3-Cyclopropyl-4-(p-tolyl)-1-(trifluoromethyl)-1H-pyrrole and 2-cyclopropyl-4-(p-tolyl)-1-

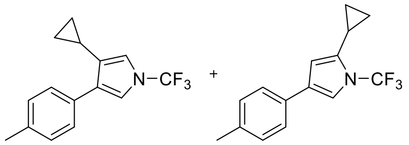

(trifluoromethyl)-1H-pyrrole (**3c** + **3c’**): Prepared according to the **general procedure A** with cyclopropylaceylene as the starting alkyne. Yield: 56%; colourless oil; crude ^{19}F NMR ratio: 91:9; ^{1}H NMR (401 MHz, $CDCl_3$) δ 7.52 – 7.45 (m, 2H, **3c**), 7.38 – 7.32 (m, 2H, **3c’**), 7.24 – 7.18 (m, 2H, **3c’**), 7.18 – 7.14 (m, 3H, **3c’**), 7.12 (q, *J* = 0.9 Hz, 1H, **3c’**), 6.98 (d, *J* = 2.5 Hz, 1H, **3c**), 6.64 (dd, *J* = 2.5, 1.0 Hz, 1H, **3c**), 6.20 (dt, *J* = 2.0, 1.0 Hz, 1H, **3c’**), 2.39 (s, 3H, **3c’**), 2.35 (s,

3H, **3c’**), 1.73 (ttd, *J* = 8.4, 5.2, 1.0 Hz, 1H, **3c**), 0.95 – 0.81 (m, 2H, **3c**), 0.63 – 0.53 (m, 2H, **3c**); ^{13}C NMR (101 MHz, $CDCl_3$) δ 136.5, 131.5, 129.5, 129.3, 128.8, 128.7, 128.0, 125.4, 119.1 (q, $^{1}J_{C\text{-}F}$ = 259.8 Hz), 115.1, 115.1, 114.3, 21.3, 21.3, 8.0, 7.5; ^{19}F NMR (377 MHz, $CDCl_3$) δ -54.76 (s, **3c’**), -57.05 (s, **3c**); HRMS (EI^+) *m/z* calcd for $C_{15}H_{14}F_3N$ $[M]^+$: 265.1078, found 265.1073.

3-Butyl-4-phenyl-1-(trifluoromethyl)-1H-pyrrole and *2-butyl-4-phenyl-1-(trifluoromethyl)-1H-pyrrole (***3d** + **3d’***)*: Yield: 48%; colourless oil; Prepared according to the **general procedure A** with 1-hexyne as the starting alkyne; crude ^{19}F NMR ratio: 97:3; ^{1}H NMR (401 MHz, $CDCl_3$) δ 7.52 – 7.48 (m, 2H, **3d’**), 7.44 – 7.34 (m, 4H + 3H’, **3d** + **3d’**), 7.33 – 7.28 (m, 1H, **3d**), 7.18 – 7.16 (m, 1H, **3d’**), 6.98 (d, *J* = 2.5 Hz, 1H, **3d**), 6.82 (dt, *J* = 2.3, 1.1 Hz, 1H, **3d**), 6.37 (dq, *J* = 1.9, 1.0 Hz, 2H, **3d’**), 2.75 – 2.66 (m, 2H, **3d’**) 2.60 – 2.52 (m, 2H, **3d**), 1.76 – 1.64 (m, 3H, **3d’**), 1.58 – 1.49 (m, 3H, **3d**), 1.50 – 1.43 (m, 2H, **3d’**), 1.42 – 1.30 (m, 2H, **3d**), 0.99 (t, *J* = 7.4 Hz, 3H, **3d’**), 0.90 (t, *J* = 7.3 Hz, 3H, **3d**); ^{13}C NMR (101 MHz, $CDCl_3$) δ 134.7, 134.2, 128.8, 128.6, 128.4, 128.4, 126.9, 126.7, 126.7, 125.5, 119.2 (q, $^{1}J_{C\text{-}F}$ = 259.8 Hz), 115.5, 115.5, 113.8, 113.7. 109.1, 32.0, 30.6, 26.3, 25.5, 22.7, 22.6, 14.0, 14.0; ^{19}F NMR (377 MHz, $CDCl_3$) δ -55.07 (s, **3d’**), -57.51 (s, **3d**); HRMS (EI^+) *m/z* calcd for $C_{15}H_{16}F_3N$ $[M]^+$: 267.1235, found 267.1239.

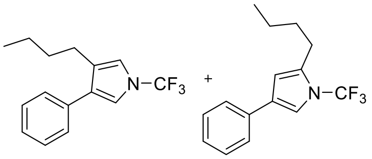

*3-Butyl-4-phenyl-1-(trifluoromethyl)-1H-pyrrole (***3d***):* ^{1}H NMR (401 MHz, $CDCl_3$) δ 7.39 (d, *J* = 1.0 Hz, 1H), 7.38 (s, 1H), 7.33 – 7.28 (m, 1H), 6.98 (d, *J* = 2.6 Hz, 1H), 6.81 (dt, *J* = 2.3, 1.1 Hz, 1H), 2.59 – 2.51 (m, 2H), 1.58 – 1.46 (m,

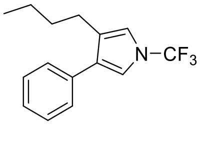

1H), 1.34 (dq, J = 14.4, 7.2 Hz, 1H), 0.89 (t, J = 7.3 Hz, 3H); ^{13}C NMR (101 MHz, $CDCl_3$) δ 134.74, 128.59, 128.39, 128.35, 126.87, 126.66, 119.2 (q, $^{1}J_{C\text{-}F}$ = 259.8 Hz), 115.5, 115.5, 32.0, 25.5, 22.7, 14.0; ^{19}F NMR (377 MHz, $CDCl_3$) δ -57.51 (s).

3-Heptyl-4-phenyl-1-(trifluoromethyl)-1H-pyrrole and *2-heptyl-4-phenyl-1-(trifluoromethyl)-1H-pyrrole* (**3e** + **3e'**): Prepared according to the **general procedure B** with 1-nonyne as the starting alkyne. Yield: 36%; colourless oil; crude ^{19}F NMR ratio: 98:2; ^{1}H NMR (401 MHz, $CDCl_3$) δ 7.52 – 7.48 (m, 2H, **3e'**), 7.39 (d, J = 4.9 Hz, 4H + 3H', **3e**+ **3e'**), 7.33 – 7.26 (m, 1H, **3e**), 7.17 (d, J = 2.0 Hz, 1H, **3e'**), 6.98 (d, J = 2.5 Hz, 1H, **3e**), 6.81 (dd, J = 2.5, 1.3 Hz, 1H, **3e**), 6.38 – 6.33 (m, 1H, **3e'**), 2.69 (t, J = 7.8 Hz, 2H, **3e'**), 2.59 – 2.50 (m, 2H, **3e**), 1.71 (q, J = 7.4 Hz, 2H, **3e'**), 1.59 – 1.47 (m, 2H, **3e**), 1.38 – 1.18 (m, 8H + 8H', **3e** + **3e'**), 0.88 (t, J = 6.8 Hz, 3H + 3H', **3e** + **3e'**); ^{13}C NMR (101 MHz, $CDCl_3$) δ 134.8, 128.9, 128.6, 128.4, 128.4, 126.9, 126.8, 126.7, 125.6, 119.2 (q, $^{1}J_{C\text{-}F}$ = 259.8 Hz), 115.5, 115.5, 31.9, 29.9, 29.6, 29.2, 25.8, 22.8, 14.2; ^{19}F NMR (377 MHz, $CDCl_3$) δ -54.54 (s, **3e'**), -56.97 (s, **3e**); HRMS (EI$^+$) *m/z* calcd for $C_{18}H_{22}F_3N$ $[M]^+$: 309.1704, found 309.1709.

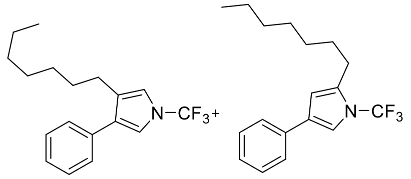

3-Dodecyl-4-phenyl-1-(trifluoromethyl)-1H-pyrrole and 2-dodecyl-4-phenyl-1-(trifluoromethyl)-1H-pyrrole (**3f** + **3f'**): Prepared according to the **general procedure B** with 1-tetradecyne as the starting alkyne. Yield: 43%; colourless oil; crude ^{19}F NMR ratio: 94:6; ^{1}H NMR (401 MHz, $CDCl_3$) δ 7.53 – 7.48 (m,

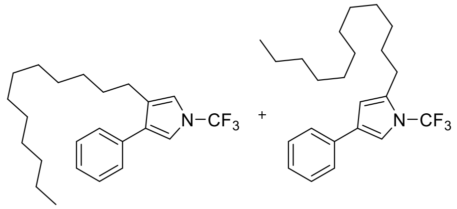

2H, **3f'**), 7.43 – 7.37 (m, 4H + 3H', **3f** + **3f'**), 7.35 – 7.20 (m, 1H, **3f**), 7.19 – 7.16 (m, 1H, **3f'**), 6.99 (d, *J* = 2.5 Hz, 1H, **3f**), 6.82 (dd, *J* = 2.4, 1.2 Hz, 1H, **3f**), 6.37 (dq, *J* = 1.9, 0.9 Hz, 1H, **3f'**), 2.70 (t, *J* = 7.8 Hz, 2H, **3f'**), 2.55 (t, *J* = 8.3 Hz, 2H, **3f**), 1.82 – 1.65 (m, 2H, **3f'**), 1.60 – 1.48 (m, 2H, **3f**), 1.41 – 1.13 (m, 18H + 18H', **3f** + **3f'**), 0.89 (t, 3H + 3H', **3f** + **3f'**); ^{13}C NMR (101 MHz, $CDCl_3$) δ 134.96, 134.76, 134.22, 128.84, 128.59, 128.41, 128.36, 126.87, 126.74, 126.70, 125.54, 115.50, 115.46, 119.2 (q, $^{1}J_{C\text{-}F}$ = 259.7 Hz), 113.75, 109.07, 32.09, 29.85, 29.84, 29.81, 29.79, 29.71, 29.63, 29.55, 29.52, 25.80, 22.86, 14.27; ^{19}F NMR (377 MHz, $CDCl_3$) δ -55.07 (s, **3f'**), -57.51 (s, **3f**); HRMS (EI$^+$) *m/z* calcd for $C_{23}H_{32}F_3N$ [M]$^+$: 379.2487, found 379.2490.

3-(Chloromethyl)-4-phenyl-1-(trifluoromethyl)-1H-pyrrole and 2-(chloromethyl)-4-phenyl-1-(trifluoromethyl)-1H-pyrrole (**3g** + **3g'**): Prepared according to the **general procedure A** with propargyl chloride as the starting alkyne. Yield: 43%; colourless oil; crude ^{19}F NMR ratio: 87:13; ^{1}H NMR (401 MHz, $CDCl_3$) δ 7.56 – 7.52 (m, 1H, **3g'**), 7.51 – 7.40 (m, 4H + 1H', **3g** + **3g'**), 7.40 – 7.32 (m, 1H + 2H', **3g** + **3g'**), 7.30 – 7.27 (m, 2H, **3g'**), 7.17 (d, *J* = 2.5 Hz, 1H, **3g**), 7.04 (d, *J* = 2.5 Hz, 1H, **3g**), 6.75 – 6.69 (m, 1H, **3g'**), 4.72 (s, 2H, **3g'**), 4.58 (s, 2H, **3g**); ^{13}C NMR (101 MHz, $CDCl_3$) δ 138.5, 133.2, 133.1, 132.6, 130.6, 129.3, 129.0, 129.0, 128.9, 128.4, 128.1, 127.5, 127.3, 127.0, 125.6, 122.4, 120.4, 118.8 (q, $^{1}J_{C\text{-}F}$ = 259.9 Hz), 118.7, 116.9, 116.6 (q, *J* = 1.8 Hz), 115.8, 114.3, 114.3, 37.9, 36.9, 36.9; ^{19}F NMR (377 MHz, $CDCl_3$) δ -55.17 (s, **3g'**), -57.74 (s, **3g**); HRMS (EI$^+$) *m/z* calcd for $C_{12}H_9ClF_3N$ [M]$^+$: 259.0376, found 259.0377.

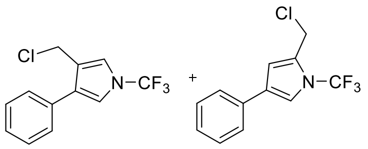

3-(2-Bromoethyl)-4-phenyl-1-(trifluoromethyl)-1H-pyrrole (**3h**): Prepared according to the **general procedure A** with 1-bromo-4-butyne as the starting alkyne. Yield: 48%; pale-yellow oil; crude ^{19}F NMR ratio to **3h'**: 93:7; ^{1}H NMR (401 MHz, $CDCl_3$) δ 7.45 – 7.37 (m, 2H), 7.36 – 7.30 (m, 4H), 6.99 (d, J = 2.6 Hz, 1H), 6.94 (dd, J = 2.3, 1.2 Hz, 1H), 3.41 (t, J = 7.8 Hz, 2H), 3.14 (t, J = 7.3 Hz, 2H); ^{13}C NMR (101 MHz, $CDCl_3$) δ 134.0, 128.9, 128.4, 128.3, 128.2, 127.3, 123.0, 119.0 (q, $^{1}J_{C\text{-}F}$ = 260.4 Hz), 116.4, 115.9, 31.8, 29.5; ^{19}F NMR (377 MHz, $CDCl_3$) δ -57.58 (s); HRMS (EI$^+$) *m/z* calcd for $C_{13}H_{11}BrF_3N$ [M]$^+$: 317.0027, found 317.0026.

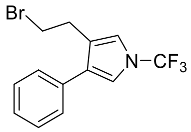

*3-(4-Iodobutyl)-4-phenyl-1-(trifluoromethyl)-1H-pyrrole and 2-(4-iodobutyl)-4-phenyl-1-(trifluoromethyl)-1H-pyrrole (***3i** + **3i'***)*: Prepared according to the **general procedure A** with 6-iodo-1-hexyne as the starting alkyne. Yield: 36%; colourless oil; crude ^{19}F NMR ratio: 98:2; ^{1}H NMR (401 MHz, $CDCl_3$) δ 7.57 – 7.48 (m, 2H, **3i'**), 7.43 – 7.36 (m, 4H + 3H', **3i** + **3i'**), 7.35 – 7.29 (m, 1H, **3i**), 7.20 – 7.16 (m, 1H, **3i'**), 6.99 (d, J = 2.5 Hz, 1H, **3i**), 6.84 (dt, J = 2.3, 1.0 Hz, 1H, **3i**), 6.40 (dq, J = 1.9, 1.0 Hz, 1H, **3i'**), 3.25 (t, J = 6.7 Hz, 2H, **3i'**), 3.15 (t, J = 6.9 Hz, 2H, **3i**), 2.78 – 2.70 (m, 2H, **3i'**), 2.59 (td, J = 7.7, 1.0 Hz, 2H, **3i**), 2.02 – 1.91 (m, 2H, **3i'**), 1.91 – 1.79 (m, 2H + 2H', **3i** + **3i'**), 1.69 – 1.59 (m, 2H, **3i**); ^{13}C NMR (101 MHz, $CDCl_3$) δ 134.5, 128.9, 128.7, 128.6, 128.4, 128.4, 127.0, 126.8, 125.7, 125.5, 119.1 (q, J = 260.0 Hz), 115.7, 115.6, 114.0, 33.3, 30.6, 24.8, 6.7; ^{19}F NMR (377 MHz, $CDCl_3$) δ -54.98 (s, **3i'**), -57.49 (s, **3i**); HRMS (EI$^+$) *m/z* calcd for $C_{15}H_{15}F_3IN$ [M]$^+$: 393.0201, found 393.0205.

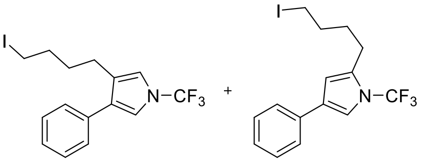

(4-Phenyl-1-(trifluoromethyl)-1H-pyrrol-3-yl)methyl benzoate and (4-phenyl-1-(trifluoromethyl)-1H-pyrrol-2-yl)methyl benzoate (**3j** + **3j'**): Prepared according to the **general procedure B** with propargyl benzoate as the starting alkyne. Yield: 50%; colourless oil; crude ^{19}F NMR ratio: 89:11; ^{1}H NMR (401 MHz, $CDCl_3$) δ 8.14 – 7.90 (m, 2H + 2H', **3j** + **3j'**), 7.65 – 7.28 (m, 10H + 10H', **3j**+ **3j'**), 7.24 (d, J = 2.5 Hz, 1H, **3j**), 7.09 (d, J = 2.5 Hz, 1H, **3j**), 6.82 – 6.77 (m, 1H, **3j'**), 5.43 (s, 2H, **3j'**), 5.31 (s, 2H, **3j**); ^{13}C NMR (101 MHz, $CDCl_3$) δ 166.5, 166.2, 133.4, 133.3, 133.2, 130.2, 129.8, 129.4, 129.0, 128.9, 128.8, 128.7, 128.7, 128.6, 128.6, 128.5, 128.3, 127.4, 127.2, 125.7, 120.4, 119.4, 119.1, 119.0 (q, $^1J_{C-F}$ = 261.2 Hz), 118.9 (q, $^1J_{C-F}$ = 261.9 Hz), 116.5, 116.5, 115.6, 58.8, 58.2; ^{19}F NMR (377 MHz, $CDCl_3$) δ -55.35 (s, **3j'**), -57.59 (s, **3j**); HRMS (EI$^+$) *m/z* calcd for $C_{19}H_{14}F_3NO_2$ [M]$^+$: 345.0977, found 345.0974.

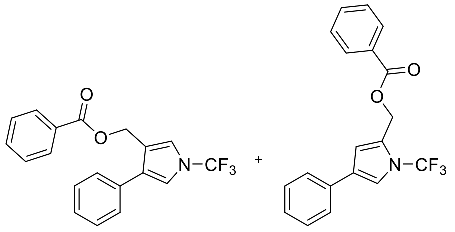

3-(3-Chloropropyl)-4-(p-tolyl)-1-(trifluoromethyl)-1H-pyrrole and 2-(3-chloropropyl)-4-(p-tolyl)-1-(trifluoromethyl)-1H-pyrrole (**3k** + **3k'**): Prepared according to the **general procedure A** with 1-chloro—4-pentyne as the starting alkyne. Yield: 60%; colourless oil; crude ^{19}F NMR ratio: 92:8; ^{1}H NMR (401 MHz, $CDCl_3$) δ 7.42 – 7.35 (m, 2H, **3k'**), 7.33 – 7.26 (m, 2H), 7.24 – 7.18 (m, 2H + 2H', **3k** + **3k'**), 7.18 – 7.14 (m, 2H, **3k'**), 6.98 (d, J = 2.4 Hz, 1H, **3k**), 6.85 (dt, J = 2.3, 1.0 Hz, 1H, **3k**), 6.39 (dq, J = 1.9, 1.0 Hz, 1H, **3k'**), 3.65 (t, J = 6.4 Hz, 2H, **3k'**), 3.52 (t, J = 6.4 Hz, 2H, **3k**), 2.94 – 2.84 (m, 2H, **3k'**), 2.74 (td, J = 7.5, 1.0 Hz, 2H, **3k**), 2.40 (s, 3H, **3k**), 2.37 (s, 3H, **3k'**), 2.23 – 2.13 (m, 2H, **3k'**), 1.96 (ddt, J = 8.6, 7.4, 6.5 Hz, 2H, **3k**); ^{13}C NMR (101 MHz, $CDCl_3$) δ 136.8, 136.6, 132.4, 131.4,

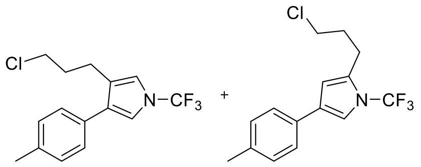

131.0, 129.6, 129.4, 128.2, 128.2, 128.2, 126.4, 125.4, 124.6, 119.1 (q, J = 260.0 Hz), 115.8, 115.7, 113.9, 113.9, 110.0, 110.0, 44.5, 32.4, 22.9, 21.3; ^{19}F NMR (377 MHz, $CDCl_3$) δ -54.96 (s, **3k'**), -57.53 (s, **3k**); HRMS (EI^+) *m/z* calcd for $C_{15}H_{15}ClF_3N$ $[M]^+$: 301.0845, found 301.0844.

3-(3-Chloropropyl)-4-(4-methoxyphenyl)-1-(trifluoromethyl)-1H-pyrrole and 2-(3-chloropropyl)-4-(4-methoxyphenyl)-1-(trifluoromethyl)-1H-pyrrole (**3l**):

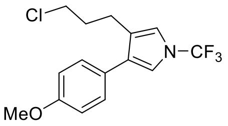

Prepared according to the **general procedure A** with 1-chloro-4-pentyne as the starting alkyne. Yield: 49%; colourless oil; crude ^{19}F NMR ratio to **3l'**: 90:10; ^{1}H NMR (401 MHz, $CDCl_3$) δ 7.32 – 7.27 (m, 2H), 6.96 – 6.91 (m, 3H), 6.83 (dt, J = 2.3, 1.0 Hz, 1H), 3.84 (s, 3H), 3.50 (t, J = 6.4 Hz, 2H), 2.81 – 2.58 (m, 2H), 2.03 – 1.85 (m, 2H); ^{13}C NMR (101 MHz, $CDCl_3$) δ 158.8, 129.4, 128.6, 128.4, 128.0, 126.9, 126.8, 124.6, 119.1 (q, J = 259.9 Hz), 115.7, 115.5, 114.2, 55.4, 44.6, 32.4, 22.9; ^{19}F NMR (377 MHz, $CDCl_3$) δ -57.52 (s); HRMS (CI^+) *m/z* calcd for $C_{15}H_{15}ClF_3NO$ $[M+H]^+$: 318.0873, found 318.0877.

3-(3-Chloropropyl)-1-(trifluoromethyl)-4-(4-(trifluoromethyl)phenyl)-1H-pyrrole and 2-heptyl-4-phenyl-1-(trifluoromethyl)-1H-pyrrole (**3m** + **3m'**):

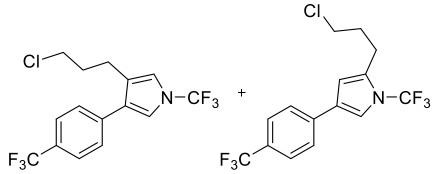

Prepared according to the **general procedure A** with 1-chloro-4-pentyne as the starting alkyne. Yield: 59%; colourless oil; crude ^{19}F NMR ratio: 95:5; ^{1}H NMR (401 MHz, $CDCl_3$) δ 7.70 – 7.62 (m, 2H, **3m**), 7.65 – 7.54 (m, 4H, **3m'**), 7.49 (dtd, J = 8.5, 1.9, 1.0 Hz, 2H), 7.05 (d, J = 2.5 Hz, 1H, **3m**), 6.89 (dt, J = 2.2, 1.0 Hz, 1H, **3m**), 6.43 (dq, J = 1.9, 0.9 Hz, 1H, **3m'**), 3.65 (t, J = 6.3 Hz, 2H, **3m**), 3.52 (t,

J = 6.4 Hz, 2H, **3m**), 2.96 – 2.87 (m, 2H, **3m'**), 2.80 – 2.71 (m, 2H, **3m**), 2.18 (ddt, *J* = 8.5, 7.4, 6.4 Hz, 2H, **3m'**), 1.96 (ddt, *J* = 8.8, 7.5, 6.3 Hz, 2H, **3m**); ^{13}C NMR (101 MHz, $CDCl_3$) δ 138.1, 138.1, 129.2 (q, *J* = 32.5 Hz), 128.4, 127.0, 125.9, 125.9, 125.7 (q, *J* = 3.8 Hz), 125.6, 124.4, 123.0, 120.3, 120.2, 118.9 (q, *J* = 260.6 Hz), 116.4, 116.3, 109.8, 44.4, 32.4, 22.8; ^{19}F NMR (377 MHz, $CDCl_3$) δ -55.14 (s, *N*-CF_3, **3m'**), -57.64 (s, *N*-CF_3, **3m**), -62.92 (s, **3m'**), -62.96 (s, **3m**); HRMS (CI$^+$) *m/z* calcd for $C_{15}H_{12}ClF_6N$ [M+H]$^+$: 356.0641, found 356.0647.

3-(3-Chloropropyl)-1-(trifluoromethyl)-4-(4-(trifluoromethyl)phenyl)-1H-pyrrole (**3m**):

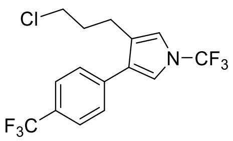

colourless oil; ^{1}H NMR (401 MHz, $CDCl_3$) δ 7.57 (dd, *J* = 65.5, 7.9 Hz, 2H), 7.05 (d, *J* = 2.4 Hz, 1H), 6.88 (dd, *J* = 2.4, 1.1 Hz, 1H), 3.52 (t, *J* = 6.3 Hz, 2H), 2.75 (td, *J* = 7.5, 1.0 Hz, 2H), 1.95 (ddt, *J* = 8.8, 7.5, 6.3 Hz, 2H); ^{13}C NMR (101 MHz, $CDCl_3$) δ 138.1, 129.2 (q, *J* = 32.5 Hz), 128.4, 127.0, 125.7 (q, *J* = 3.8 Hz), 124.4, 123.0, 120.3, 118.9 (q, *J* = 260.7 Hz), 116.4, 116.3, 44.4, 32.4, 22.8; ^{19}F NMR (377 MHz, $CDCl_3$) δ -57.64 (s, *N*-CF_3), -62.97 (s).

3-(3-Chloropropyl)-4-(4-fluorophenyl)-1-(trifluoromethyl)-1H-pyrrole and 2-(3-chloropropyl)-4-(4-fluorophenyl)-1-(trifluoromethyl)-1H-pyrrole (**3n** + **3n'**):

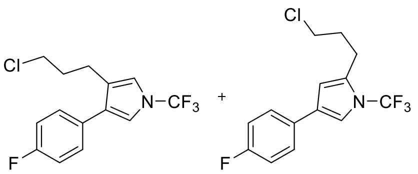

Prepared according to the **general procedure A** with 1-chloro-4-pentyne as the starting alkyne. Yield: 70%; colourless oil; crude ^{19}F NMR ratio: 92:8; ^{1}H NMR (400 MHz, $CDCl_3$) δ 7.58 (dd, *J* = 8.7, 0.8 Hz, 1H, **3n'**), 7.50 – 7.39 (m, 3H, **3n'**), 7.38 – 7.32 (m, 1H, **3n**), 7.21 – 7.04 (m, 2H + 1H', **3n** + **3n'**), 6.99 (d, *J* = 2.5 Hz, 1H, **3n**), 6.87 (dt, *J* = 2.3, 1.0 Hz, 1H, **3n**), 6.37 (dq, *J* = 1.8, 0.9

Hz, 1H, **3n’**), 3.66 (t, *J* = 6.4 Hz, 2H, **3n’**), 3.53 (t, *J* = 6.4 Hz, 2H, **3n**), 2.93 (t, *J* = 7.6 Hz, 2H, **3n’**), 2.82 – 2.63 (m, 2H, **3n**), 2.19 (dq, *J* = 8.0, 6.5 Hz, 2H, **3n’**), 1.96 (ddt, *J* = 8.7, 7.4, 6.4 Hz, 2H, **3n**); ^{13}C NMR (101 MHz, $CDCl_3$) δ 162.07 (d, *J* = 246.1 Hz), δ 161.9 (d, *J* = 245.5 Hz), 147.1, 133.4, 132.6, 130.2, 130.2, 129.9, 129.8, 127.3, 127.0, 126.9, 124.8, 124.5, 124.4, 124.0, 122.8, 120.2, 118.9 (q, *J* = 260.2 Hz), 119.1, 117.6, 115.8, 115.7, 115.6, 115.5, 115.4, 115.2, 115.0, 113.9, 109.8, 44.30, 32.3, 22.7; ^{19}F NMR (376 MHz, $CDCl_3$) δ -54.52 (s, **3n’**), -57.08 (s, **3n**), -115.42 to -115.59 (m, 1F, **3n**), -115.80 to -116.00 (m, 1F, **3n’**); HRMS (CI^+) *m/z* calcd for $C_{14}H_{12}ClF_4N$ $[M + H]^+$: 306.0673, found 306.0674.

*3-(3-Chloropropyl)-4-(4-fluorophenyl)-1-(trifluoromethyl)-1H-pyrrole (***3n***)*: colourless oil;

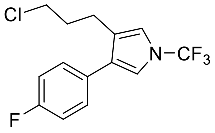

^{1}H NMR (401 MHz, $CDCl_3$) δ 7.38 – 7.28 (m, 2H), 7.14 – 7.04 (m, 2H), 6.96 (d, *J* = 2.5 Hz, 1H), 6.84 (dt, *J* = 2.3, 1.0 Hz, 1H), 3.50 (t, *J* = 6.4 Hz, 2H), 2.84 – 2.44 (m, 2H), 2.12 – 1.76 (m, 2H); ^{13}C NMR (101 MHz, $CDCl_3$) δ 162.2 (d, *J* = 246.1 Hz), 130.4, 130.3, 130.0, 129.9, 127.4, 124.5, 118.9 (q, *J* = 260.2 Hz), 115.9, 115.8, 115.8, 115.6, 44.5, 32.4, 22.8; ^{19}F NMR (377 MHz, $CDCl_3$) δ -57.58 (s, *N*-CF_3), -116.04 (m, 1F).

3-(3-Chloropropyl)-4-(4-nitrophenyl)-1-(trifluoromethyl)-1H-pyrrole (**3o**): Prepared

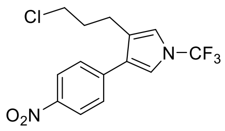

according to the **general procedure A** with 1-chloro-4-pentyne as the starting alkyne. Yield: 27%; colourless oil; crude ^{19}F NMR ratio to **3o’**: 96:4; ^{1}H NMR (401 MHz, $CDCl_3$) δ 8.29 – 8.24 (m, 2H), 7.61 – 7.51 (m, 2H), 7.12 (d, *J* = 2.5 Hz, 1H), 6.91 (dt, *J* = 2.3, 1.0 Hz, 1H), 3.53 (t, *J* = 6.3 Hz, 2H), 2.82 – 2.73 (m, 2H), 1.96 (ddt, *J* = 8.9, 7.5, 6.3 Hz, 2H); ^{13}C NMR (101 MHz, $CDCl_3$) δ 146.8, 141.3, 128.6,

126.2, 126.1, 124.4, 124.2, 118.8 (q, J = 261.1 Hz), 116.9, 116.7, 44.3, 32.4, 22.9; ^{19}F NMR (377 MHz, $CDCl_3$) δ -57.73 (s); HRMS (EI$^+$) *m/z* calcd for $C_{14}H_{12}ClF_3N_2O_2$ $[M]^+$: 332.0539, found 332.0538.

3-(3-Chloropropyl)-1-(perfluoroethyl)-4-(p-tolyl)-1H-pyrrole and 2-(3-chloropropyl)-1-(perfluoroethyl)-4-(p-tolyl)-1H-pyrrole (**3p** + **3p'**): Prepared according to the **general procedure A** with 1-chloro-4-pentyne as the starting alkyne. Yield: 51%; colourless oil; crude ^{19}F NMR ratio: 97:3; ^{1}H NMR (401 MHz, $CDCl_3$) δ 7.39 (d, J = 8.1 Hz, 2H, **3p'**), 7.28 (d, J = 8.2 Hz, 2H, **3p**), 7.24 – 7.16 (m, 2H + 2H', **3p** + **3p'**), 7.06 (q, J = 1.0 Hz, 1H, **3p'**), 6.93 (d, J = 2.5 Hz, 1H, **3p**), 6.84 – 6.73 (m, 1H, **3p**), 6.42 (dt, J = 1.9, 0.9 Hz, 1H, **3p'**), 3.64 (t, J = 6.4 Hz, 2H, **3p'**), 3.51 (t, J = 6.4 Hz, 2H, **3p**), 2.91 – 2.83 (m, 2H, **3p'**), 2.85 – 2.62 (m, 2H, **3p**), 2.39 (s, 3H, **3p**), 2.37 (s, 3H, **3p'**), 2.22 – 2.12 (m, 2H, **3p'**), 1.96 (ddt, J = 8.7, 7.5, 6.4 Hz, 2H, **3p**); ^{13}C NMR (101 MHz, $CDCl_3$) δ 136.8, 131.3, 129.6, 129.4, 128.5, 128.2, 125.4, 124.8, 122.1 (t, J = 47.5 Hz), 119.3 (t, J = 47.4 Hz), 116.4 (q, $^1J_{C\text{-}F}$ = 47.5 Hz), 116.3, 116.2, 113.6 (q, $^1J_{C\text{-}F}$ = 47.4 Hz), 113.4 (q, J = 41.7 Hz), 110.8 (q, J = 41.7 Hz), 108.2 (q, J = 41.5 Hz); 44.5, 32.4, 23.0, 21.3; ^{19}F NMR (377 MHz, $CDCl_3$) δ -84.98 (s, $-CF_3$, **3p'**), -85.78 (s, $-CF_3$, **3p**), -96.06 (s, *N*-CF_2-, **3p'**), -98.97 (s, *N*-CF_2-, **3p**); HRMS (EI$^+$) *m/z* calcd for $C_{16}H_{15}ClF_5N$ $[M]^+$: 351.0813, found 351.0813.

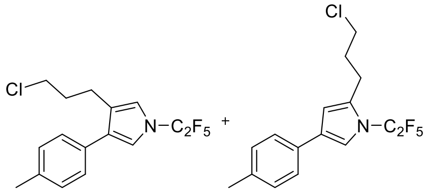

3-(3-Chloropropyl)-1-(perfluoroethyl)-4-(4-(trifluoromethyl)phenyl)-1H-pyrrole and 2-(3-chloropropyl)-1-(perfluoroethyl)-4-(4-(trifluoromethyl)phenyl)-1H-pyrrole (**3q** + **3q'**): Prepared according to the **general procedure A** with 1-chloro-4-pentyne as the starting alkyne. Yield: 42%; colourless oil; crude ^{19}F NMR ratio: 98:2; ^{1}H NMR (401 MHz, $CDCl_3$) δ 7.67 – 7.56 (m, 4H + 4H', **3q'** + **3q'**), 7.58 (dd, *J* = 62.7, 8.1 Hz, 4H, **3q**), 7.17 – 7.13 (m, 1H, **3q'**), 7.00 (d, *J* = 2.4 Hz, 1H, **3q**), 6.84 (d, *J* = 2.4 Hz, 1H, **3q**), 6.46 (dt, *J* = 2.1, 1.0 Hz, 1H, **3q'**), 3.64 (t, *J* = 6.3 Hz, 2H, **3q'**), 3.52 (t, *J* = 6.3 Hz, 2H, **3q**), 2.89 (t, *J* = 7.4 Hz, 2H, **3q'**), 2.92 – 2.61 (m, 2H, **3q**), 2.23 – 2.13 (m, 2H, **3q'**), 1.96 (ddt, *J* = 8.8, 7.5, 6.3 Hz, 2H, **3q**); ^{13}C NMR (101 MHz, $CDCl_3$) δ 138.1, 138.0, 138.0, 129.2 (q, *J* = 32.6 Hz), 128.4, 127.2, 125.7 (q, *J* = 3.8 Hz), 124.6, 123.0, 122.0 (t, *J* = 47.1 Hz), 120.3, 119.2 (t, *J* = 47.0 Hz), 116.9, 116.9, 116.1 (t, *J* = 47.1 Hz), 113.5 (t, $^{1}J_{C\text{-}F}$ = 47.1 Hz), 113.4 (q, *J* = 41.7 Hz), 110.7 (q, *J* = 41.8 Hz), 108.1 (q, *J* = 41.9 Hz), 44.4, 32.4, 22.9; ^{19}F NMR (377 MHz, $CDCl_3$) δ -62.95 (CF3, **3q'**), -62.98 (CF_3, **3q**), -85.05 (-CF_3, **3q'**), -85.84 (-CF_3, **3q**), -96.33 (*N*-CF_2, **3q'**), -99.26 (*N*-CF_2, **3q**); HRMS (EI$^+$) *m/z* calcd for $C_{16}H_{12}ClF_8N$ $[M]^+$: 405.0531, found 405.0539.

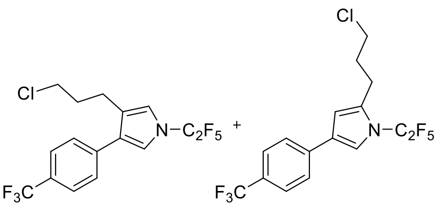

*4-(4-Phenyl-1-(trifluoromethyl)-1H-pyrrol-3-yl)butanenitrile (***4***)*: Yield: 32%; colourless oil; crude ^{19}F NMR ratio to **4'** and **5**: 63:6:31; ^{1}H NMR (401 MHz, $CDCl_3$) δ 7.47 – 7.29 (m, 5H), 7.00 (d, *J* = 2.5 Hz, 1H), 6.86 (dt, *J* = 2.3, 1.0 Hz, 1H), 2.84 – 2.56 (m, 2H), 2.28 (t, *J* = 7.1 Hz, 2H), 1.89 – 1.72 (m, 2H); ^{13}C NMR (101 MHz, $CDCl_3$) δ 134.14, 128.95, 128.86, 128.26, 128.18, 127.29, 123.73, 119.48, 119.0

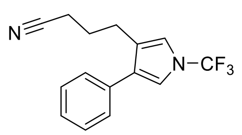

(q, J = 260.7 Hz), 116.15, 116.03, 25.39, 24.62, 16.72; ^{19}F NMR (377 MHz, $CDCl_3$) δ -57.58 (s); HRMS (CI^+) *m/z* calcd for $C_{15}H_{13}F_3N_2$ $[M + H]^+$: 279.1109, found 279.1107.

*3,4-Diphenyl-1-(trifluoromethyl)-1H-pyrrole-2-carboxylic acid (***6***)*: The crude mixture of **2a** + **2a'** (0.2 mmol) prepared according to the **general procedure A** was dried under reduced pressure, then THF (8 mL) was added. The solution was cooled down to -78 °C and *n*-BuLi (2.5M in hexane, 160 µL, 0.4 mmol, 2 eq.) was added. The reaction was stirred for 30 minutes under nitrogen atmosphere. Then, excess of CO_2 (in form of dry ice, ~2g) was added to the reaction mixture. After 15 minutes of stirring the reaction was quenched with acetic acid (1 mL). The reaction mixture was extracted with ether; the organic layer was washed with brine (3 × 20 mL), brine/water (1:1, 3 × 20 mL) and water (3 × 20 mL). Solvent evaporation afforded pure product. Yield: 75%, white crystalline solid; ^{1}H NMR (401 MHz, $CDCl_3$) δ 7.39 (s, 1H), 7.34 – 7.28 (m, 3H), 7.23 – 7.16 (m, 5H), 7.04 – 6.97 (m, 2H); ^{13}C NMR (126 MHz, $CDCl_3$) δ 161.8, 136.4, 132.8, 132.3, 130.28, 128.45, 128.31, 127.95, 127.83, 127.15, 122.08, 118.57 (q, J = 264.3 Hz); ^{19}F NMR (377 MHz, $CDCl_3$) δ -53.50 (s); HRMS (CI^+) *m/z* calcd for $C_{18}H_{12}F_3NO_2$ $[M + H]^+$: 331.0820, found 331.07778

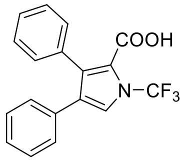

References

[1] Z. E. Blastik, S. Voltrová, V. Matoušek, B. Jurásek, D. W. Manley, B. Klepetářová, P. Beier, *Angew. Chem. - Int. Ed.* **2017**, *56*, 346–349.

^{1}H NMR (401 MHz, $CDCl_3$) of **2a** + **2a'**

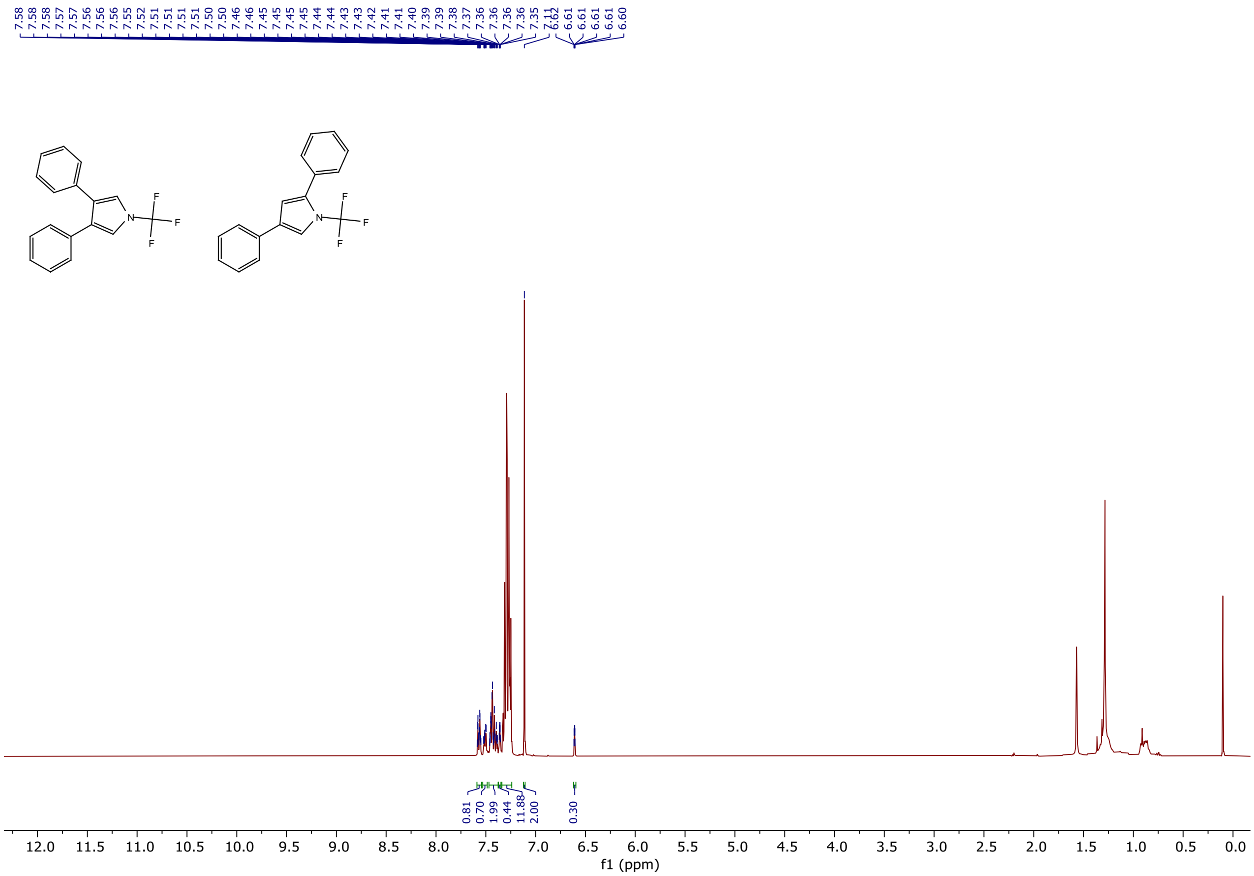

^{13}C NMR (101 MHz, $CDCl_3$) of **2a** + **2a'**

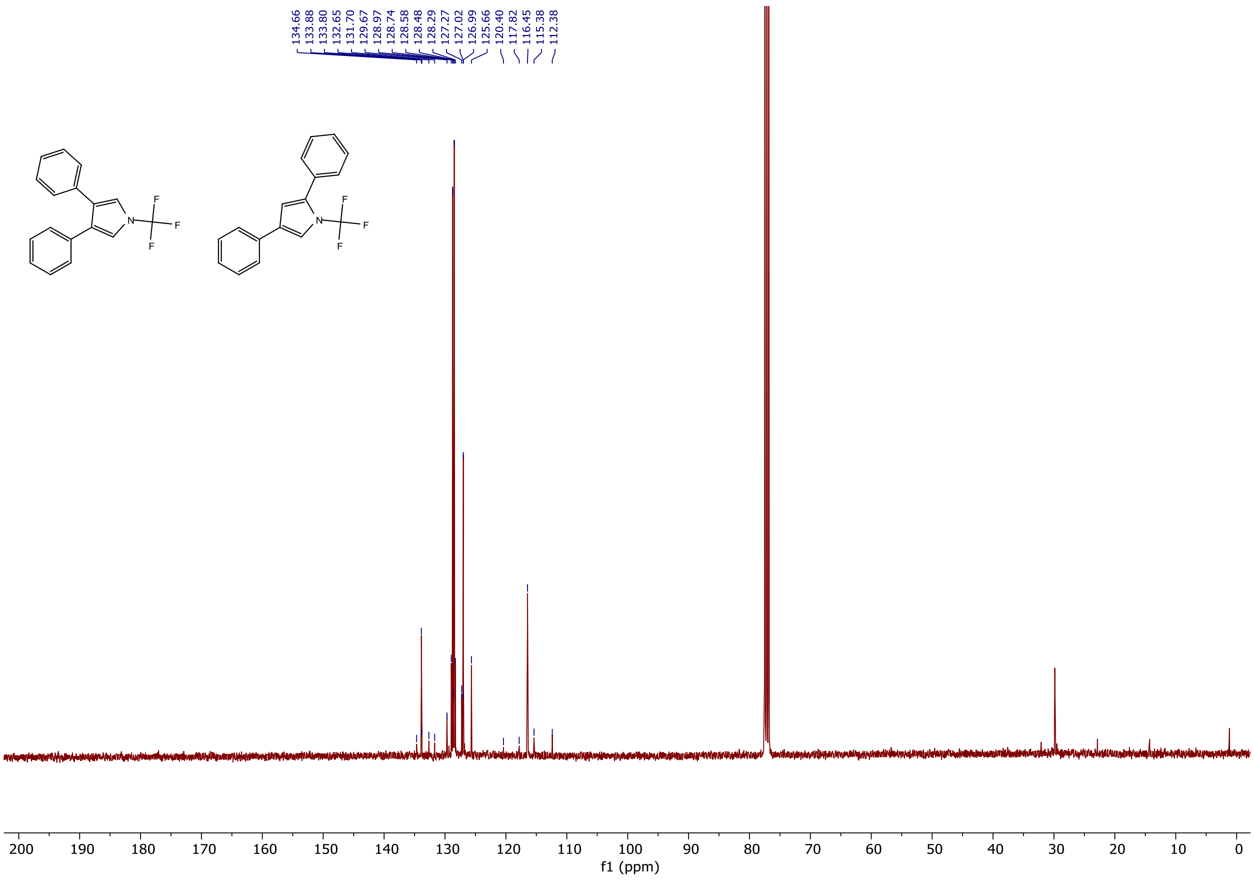

^{19}F NMR (377 MHz, $CDCl_3$) of **2a** + **2a’**

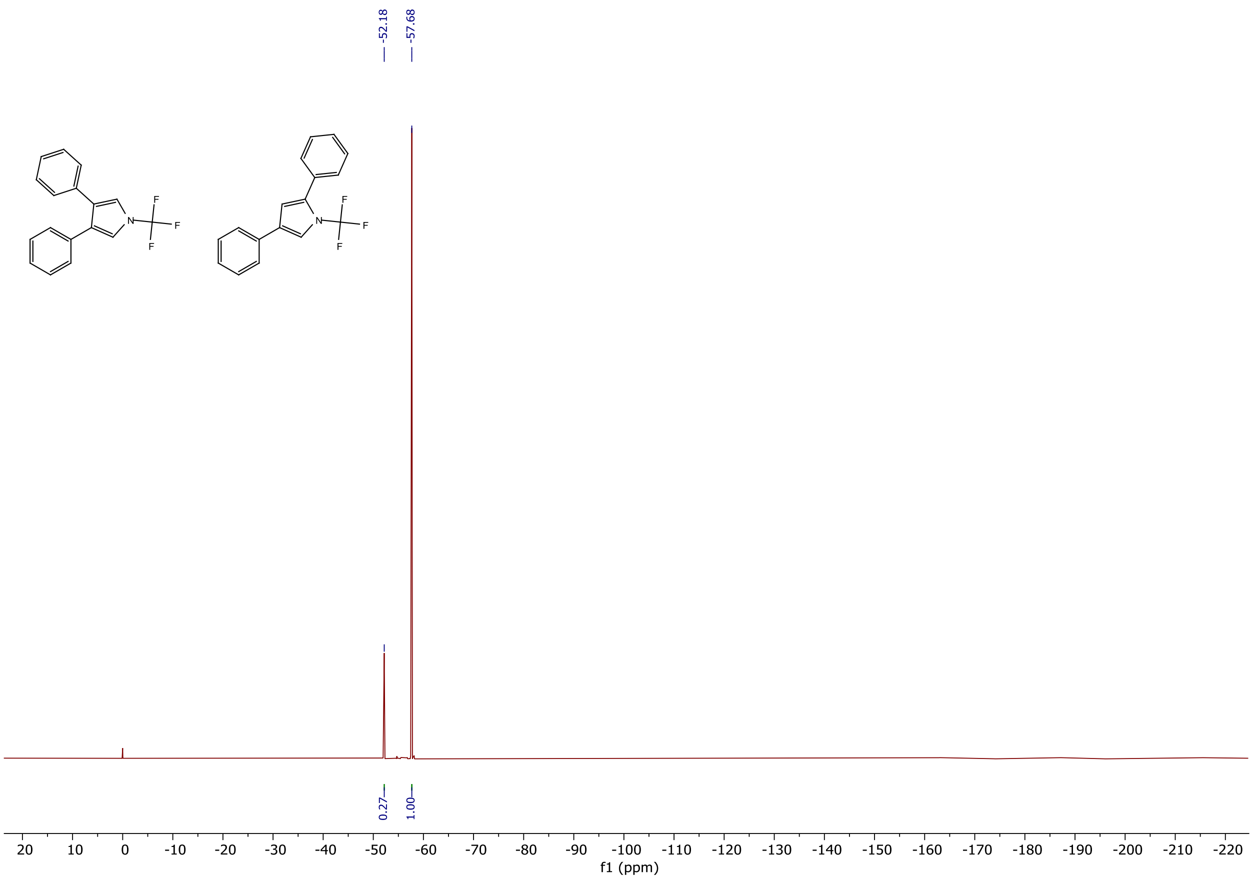

^{1}H NMR (401 MHz, $CDCl_3$) of **2a**

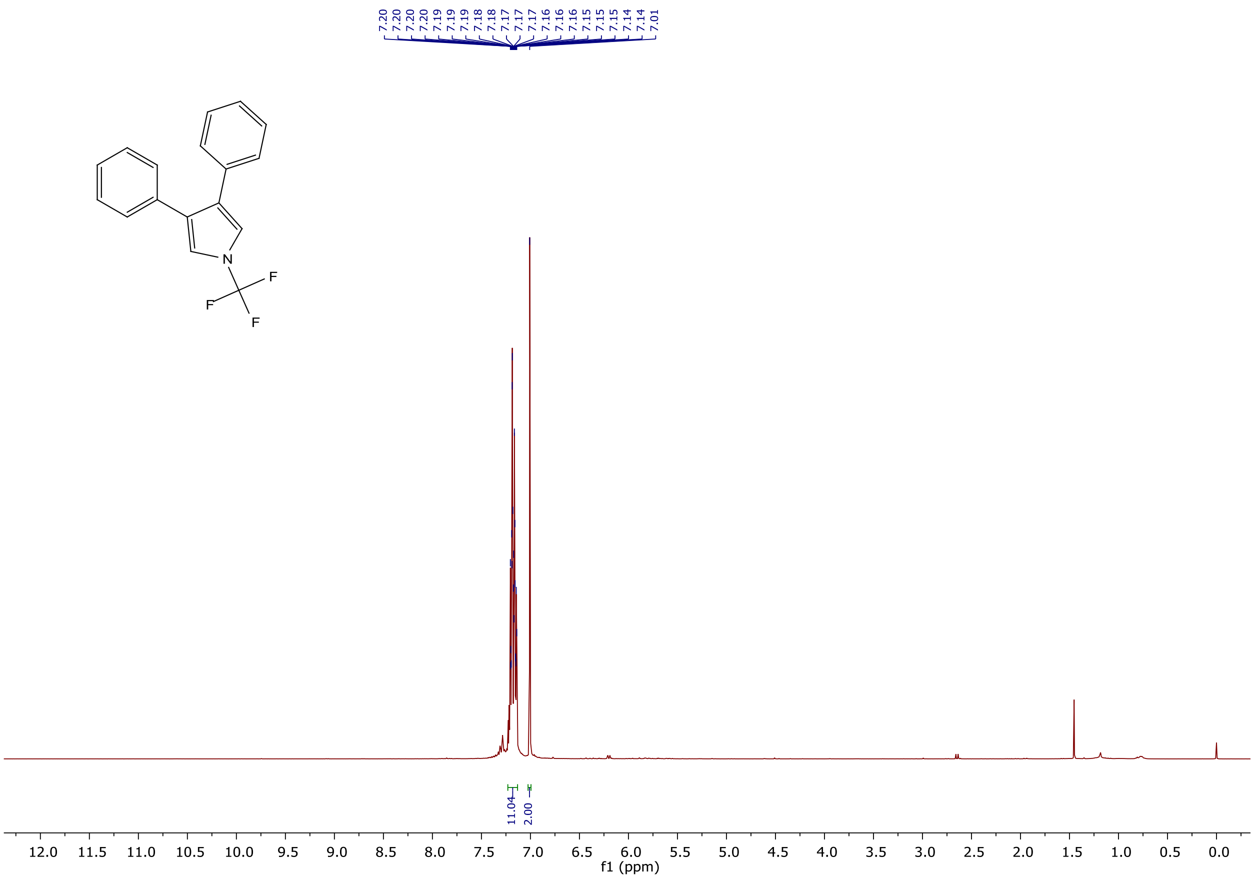

^{13}C NMR (101 MHz, $CDCl_3$) of **2a**

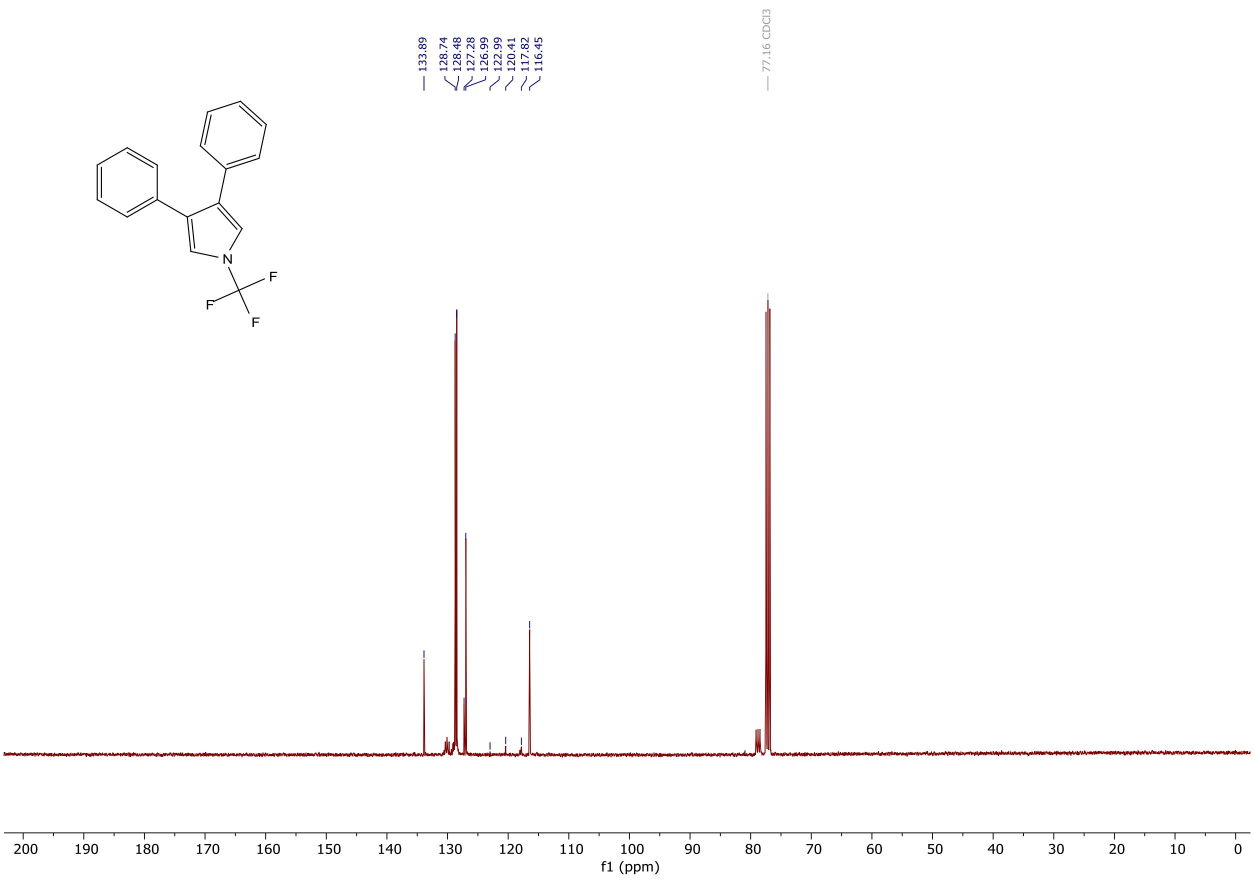

^{19}F NMR (377 MHz, $CDCl_3$) of **2a**

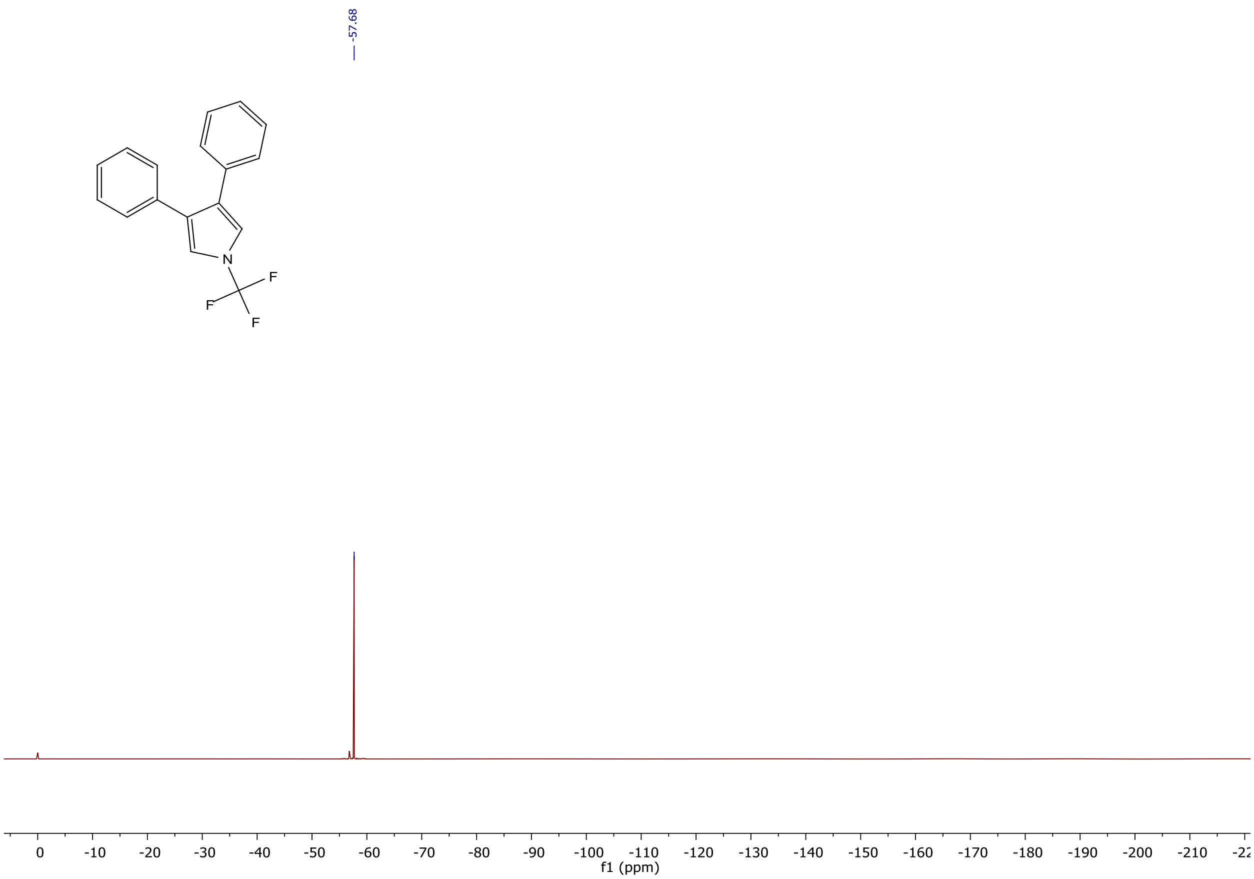

^{1}H NMR (401 MHz, $CDCl_3$) of **2a’**

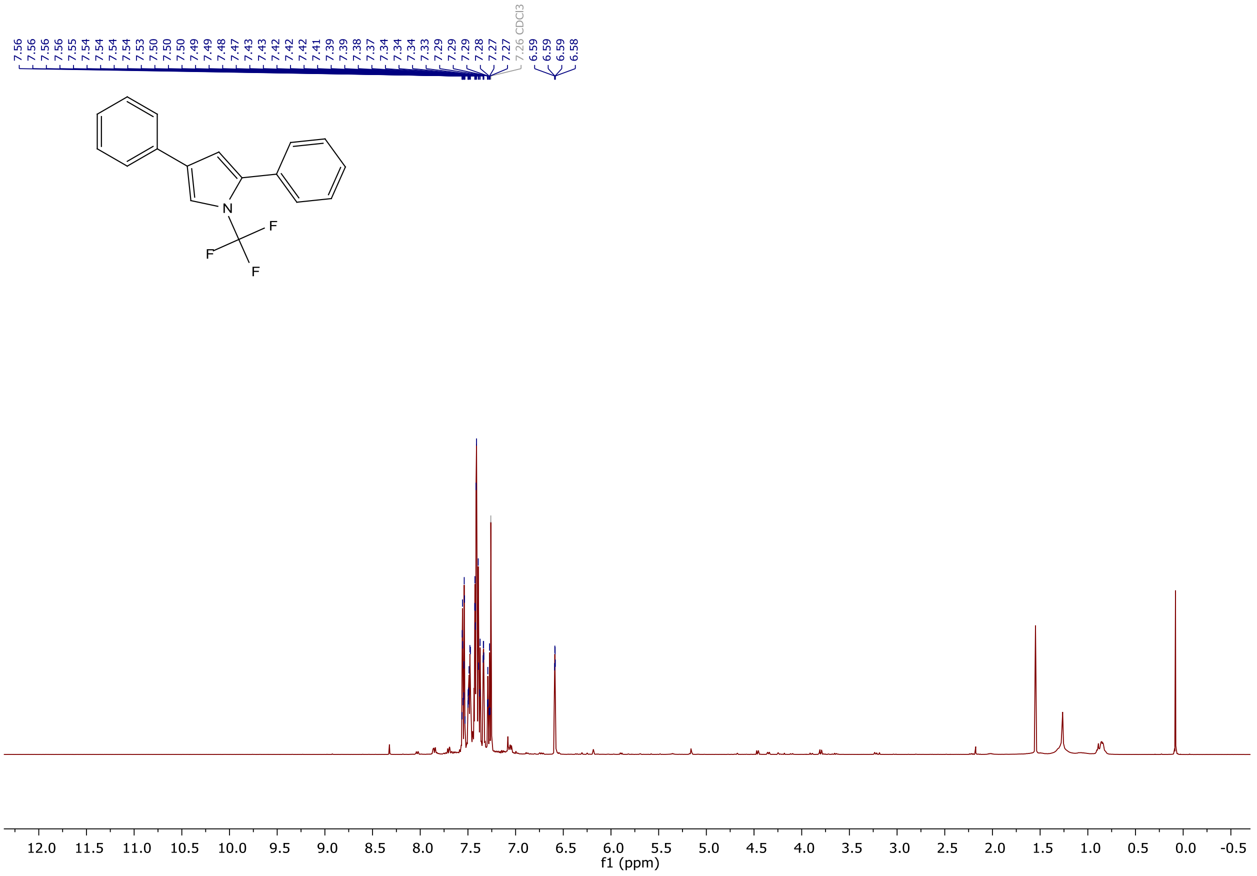

^{13}C NMR (101 MHz, $CDCl_3$) of **2a’**

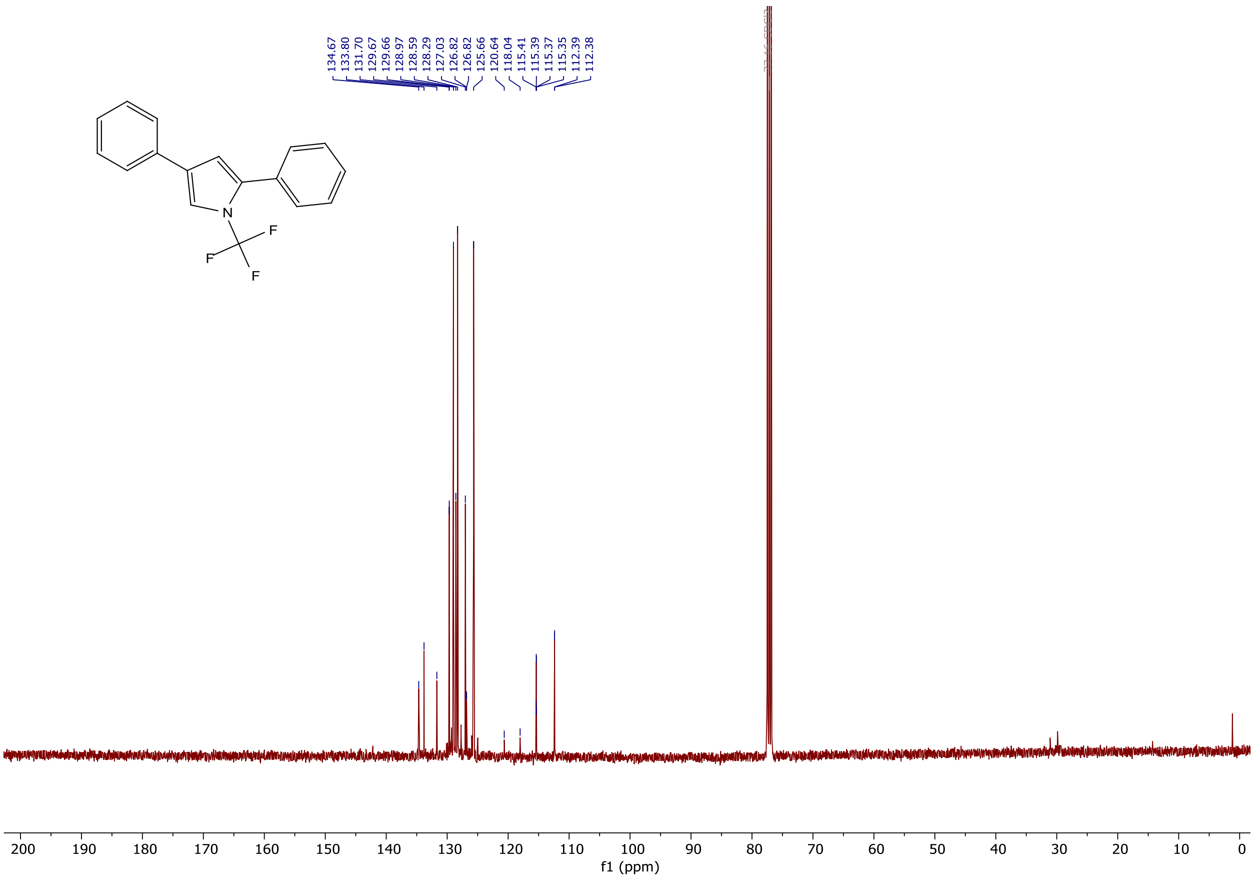

^{19}F NMR (377 MHz, $CDCl_3$) of **2a’**

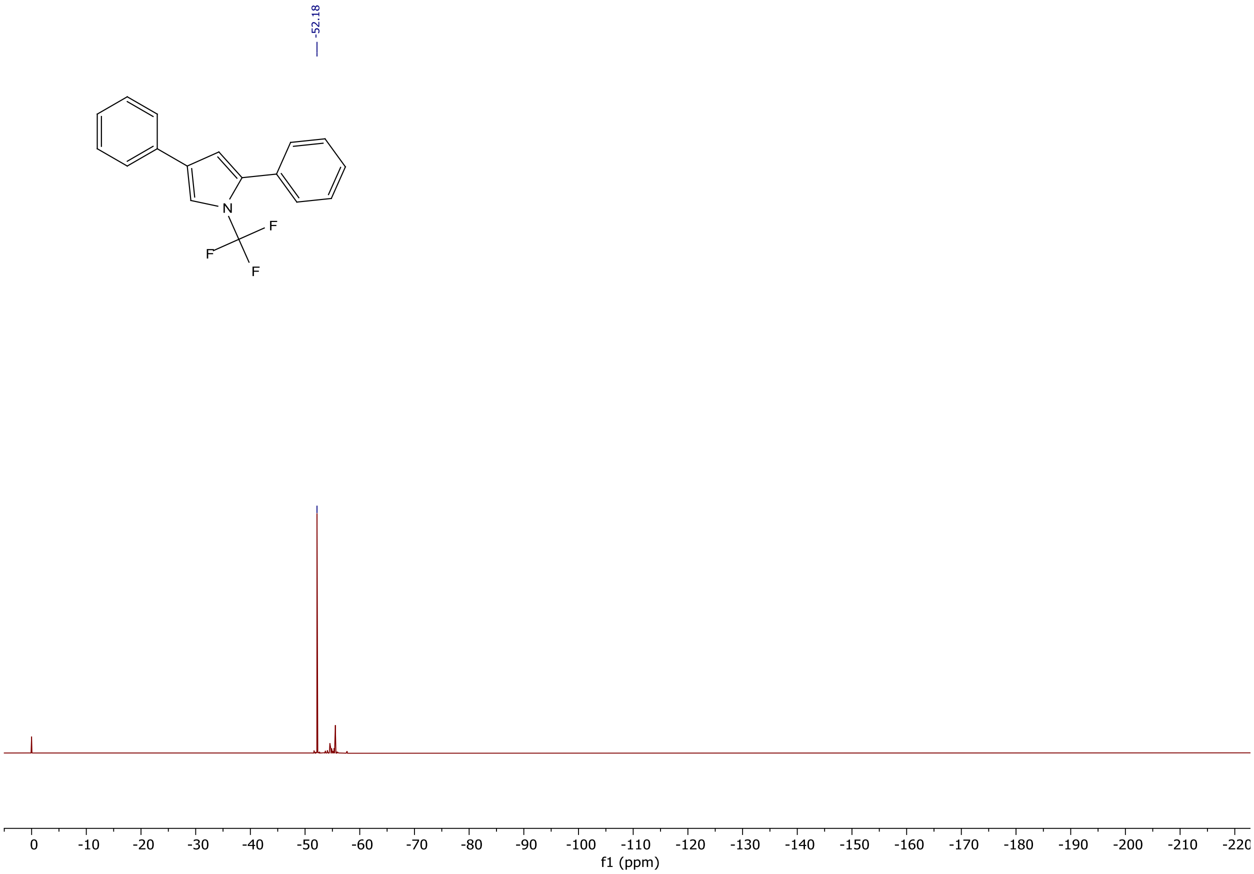

^{1}H NMR (401 MHz, $CDCl_3$) of **2b** + **2b'**

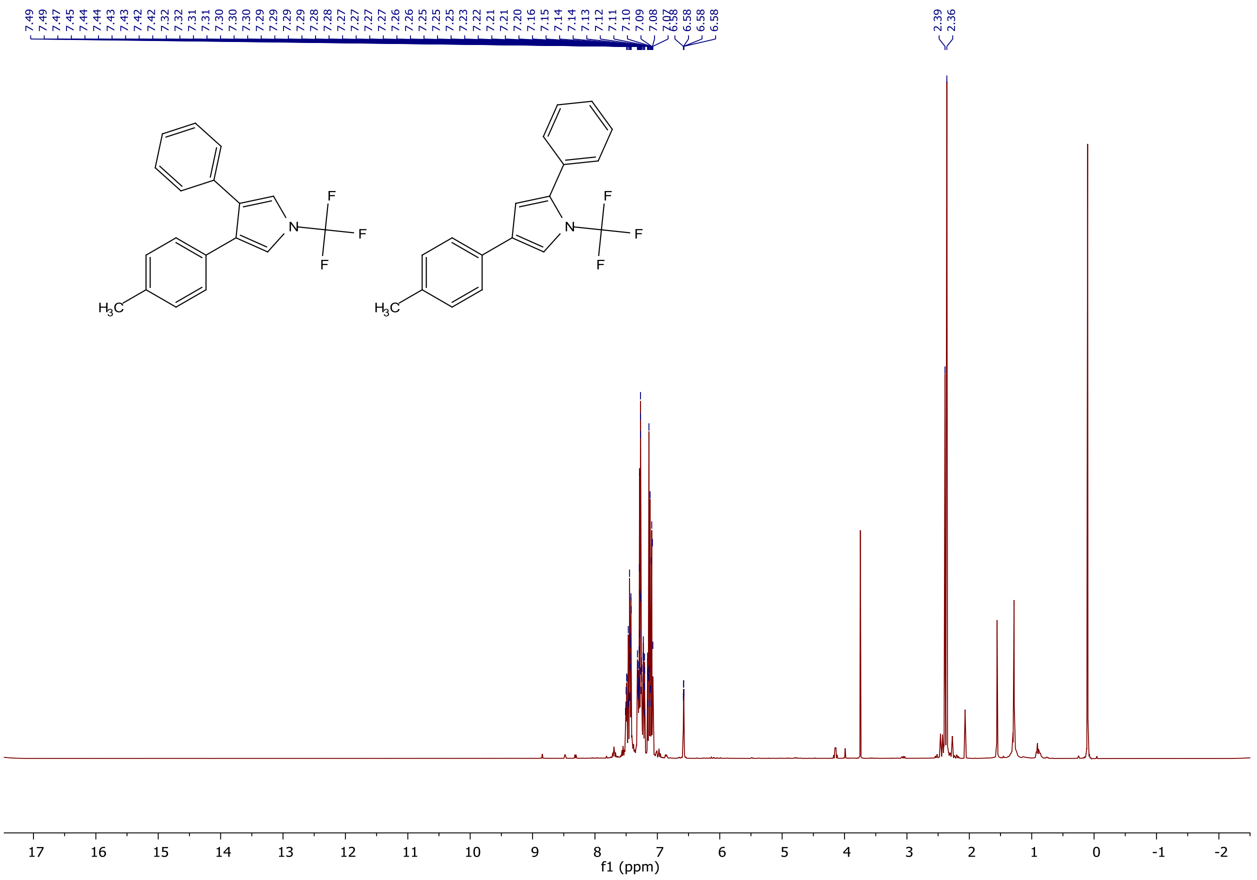

^{13}C NMR (101 MHz, $CDCl_3$) of **2b** + **2b’**

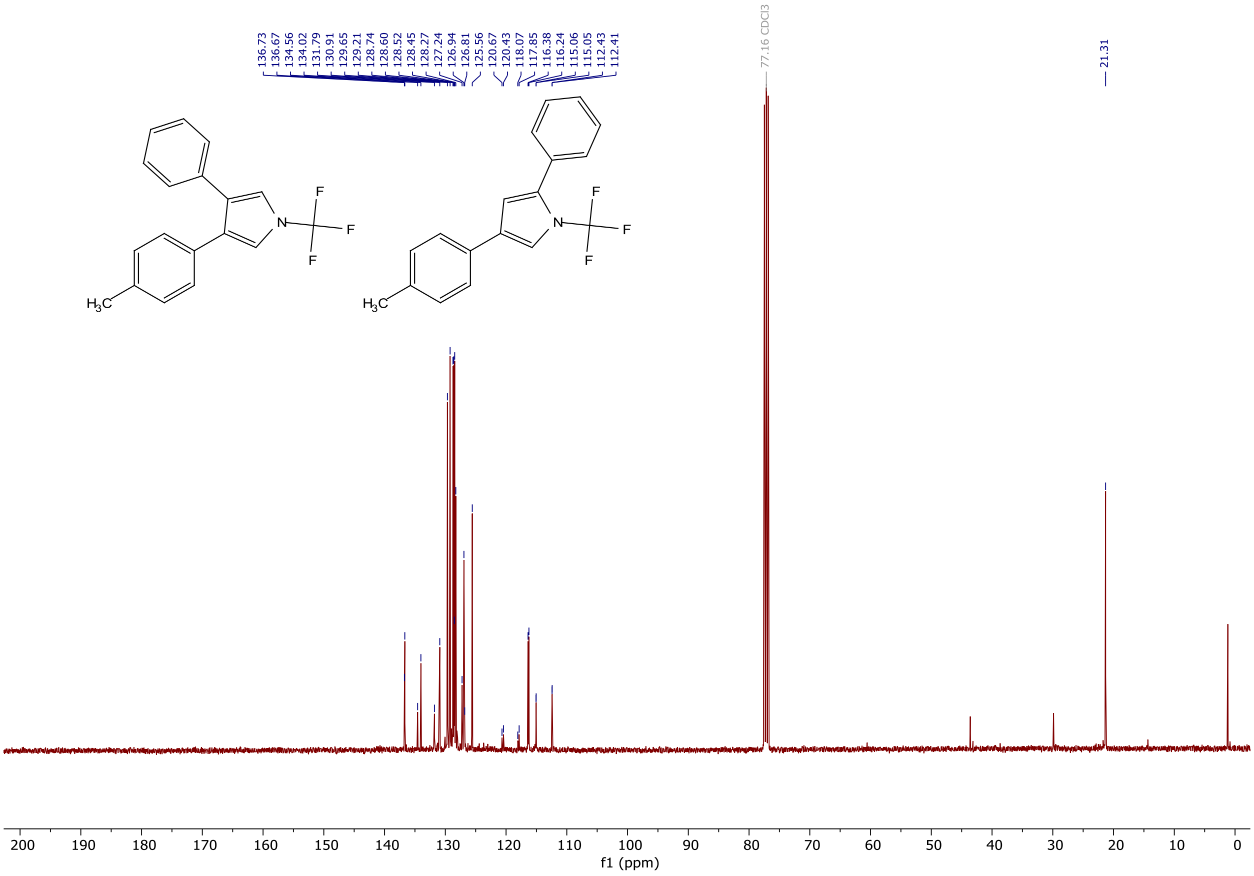

^{19}F NMR (377 MHz, $CDCl_3$) of **2b** + **2b'**

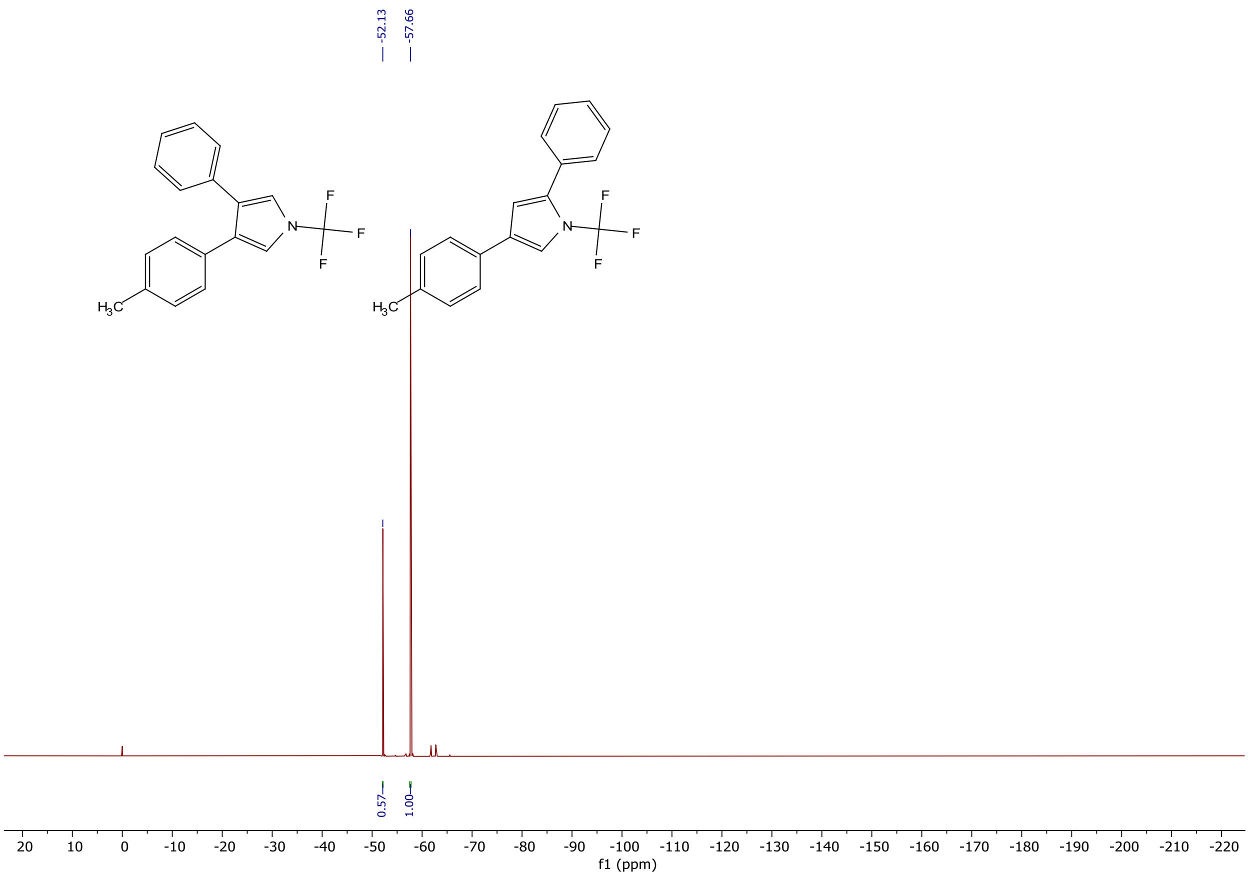

^{1}H NMR (401 MHz, $CDCl_3$) of **2c** + **2c’**

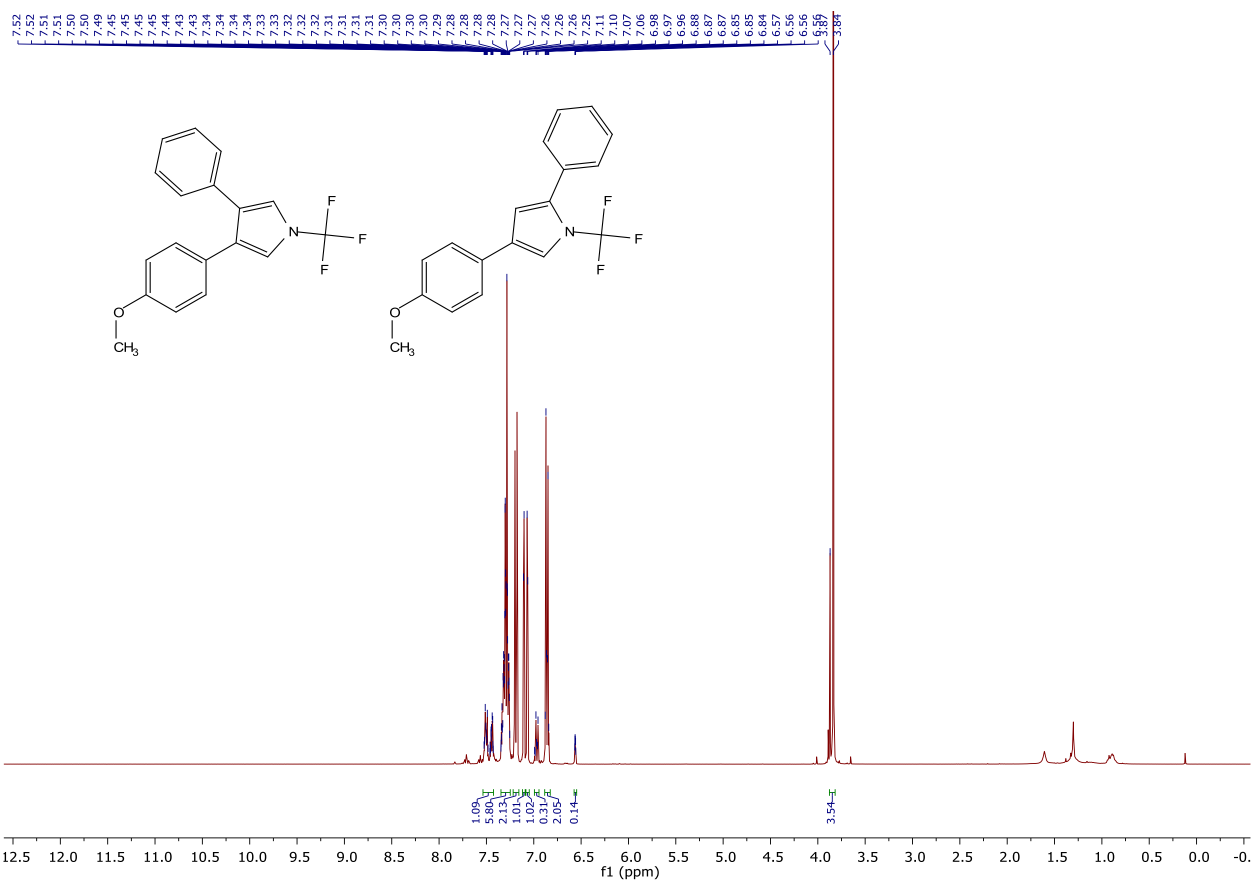

^{13}C NMR (101 MHz, $CDCl_3$) of **2c** + **2c'**

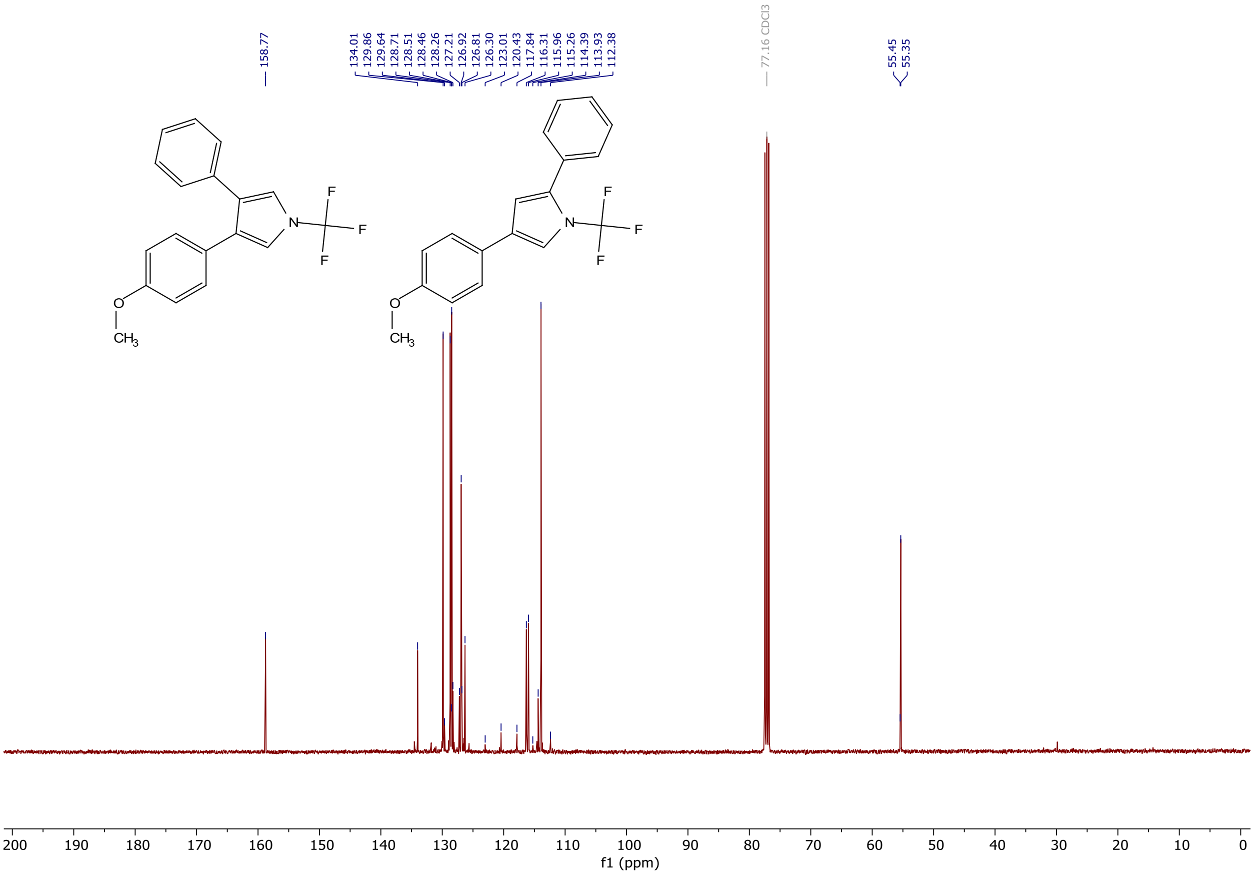

^{19}F NMR (377 MHz, $CDCl_3$) of **2c** + **2c'**

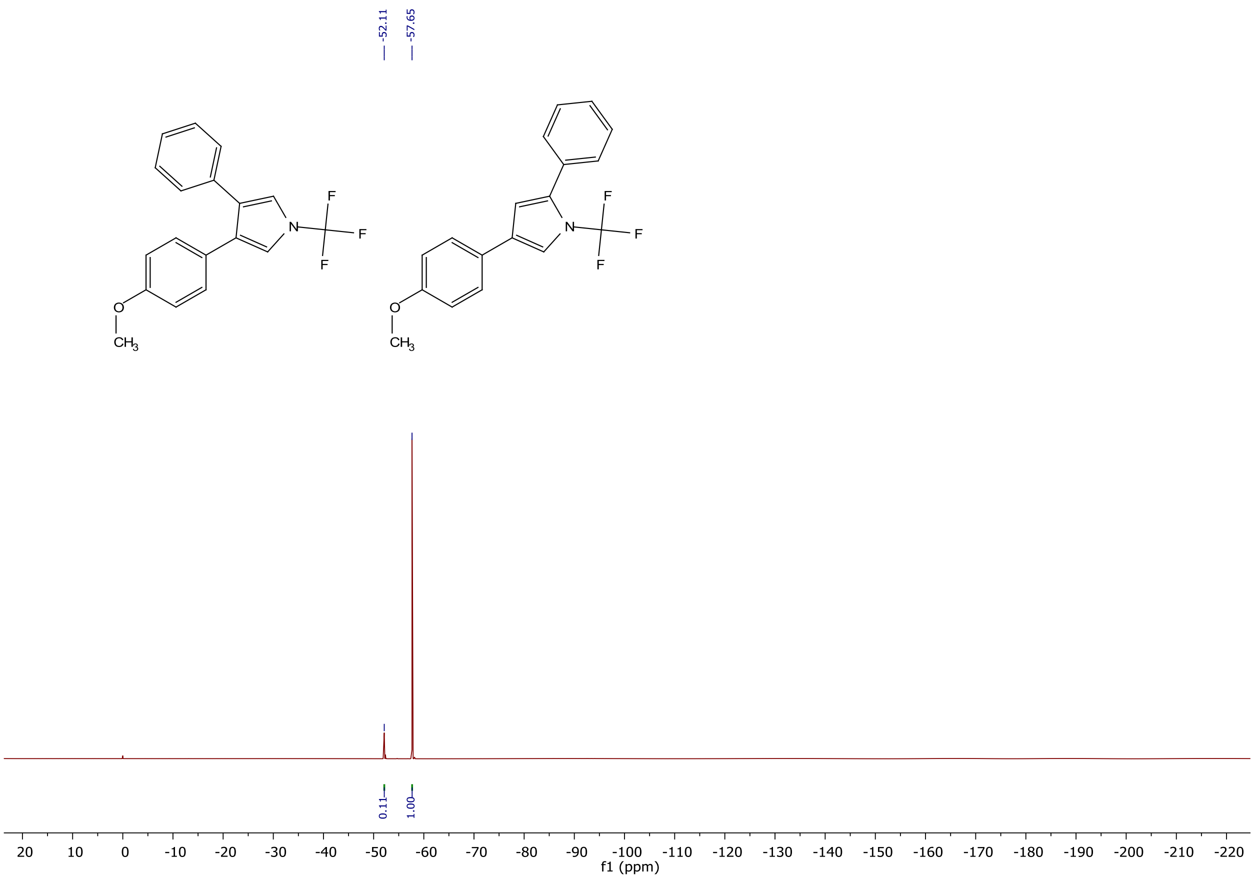

^{1}H NMR (401 MHz, $CDCl_3$) of **2d** + **2d'**

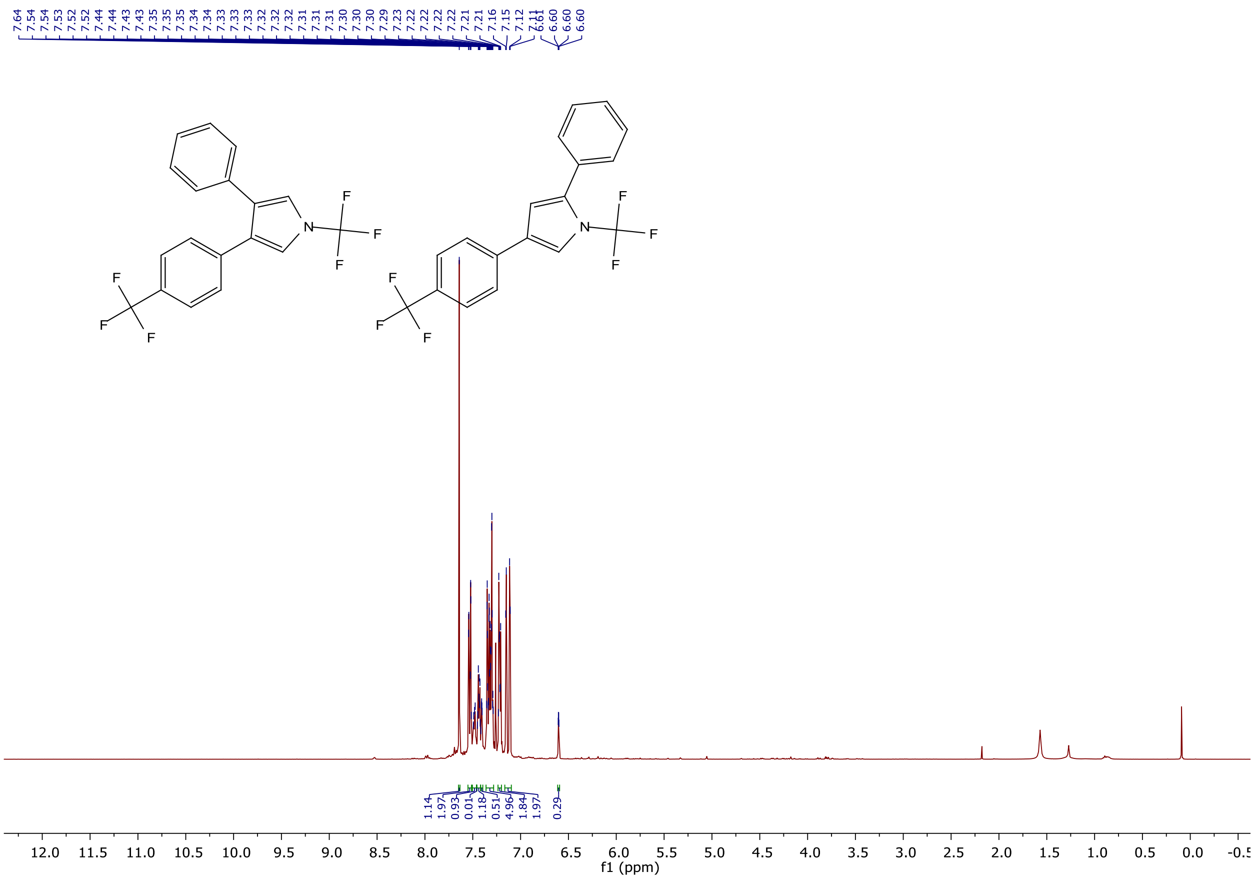

^{13}C NMR (101 MHz, $CDCl_3$) of **2d** + **2d'**

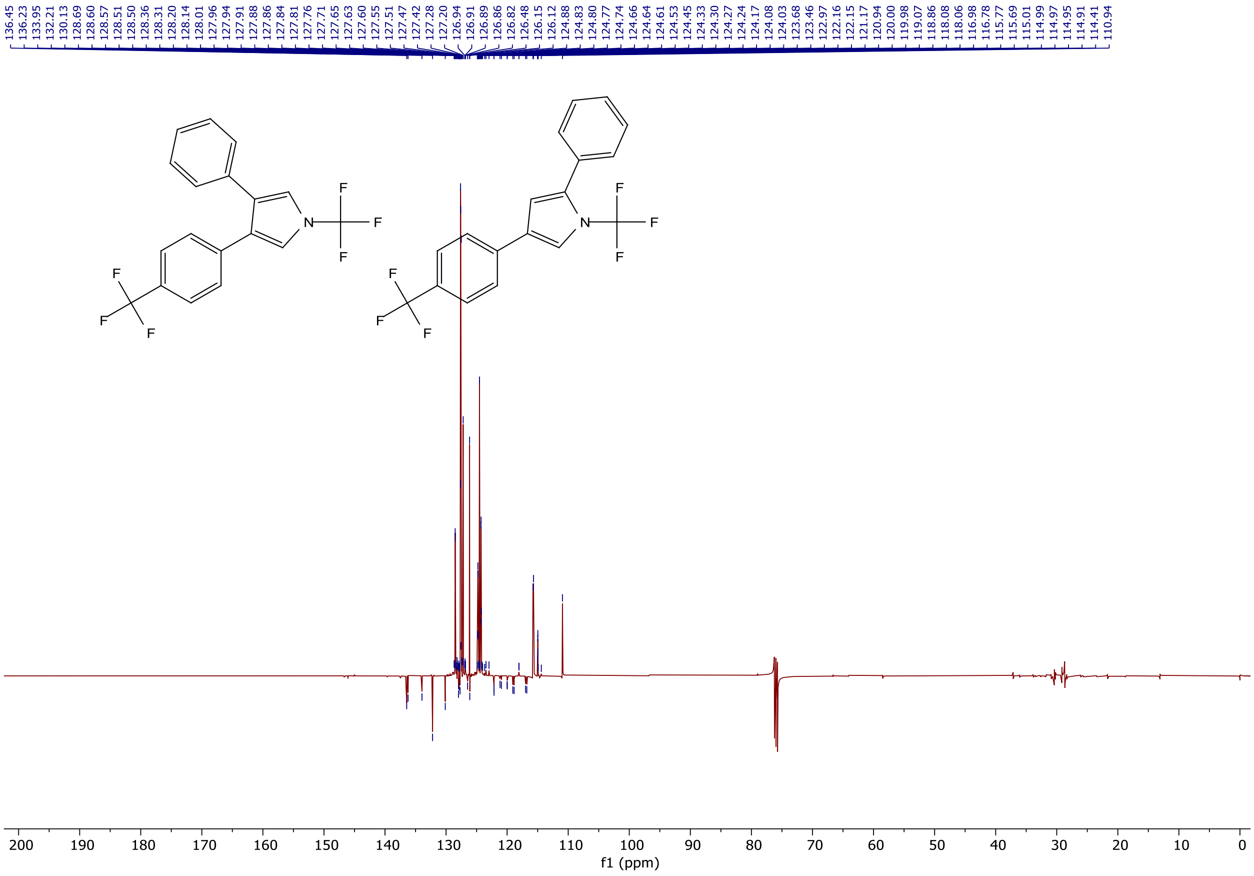

^{19}F NMR (377 MHz, $CDCl_3$) of **2d** + **2d’**

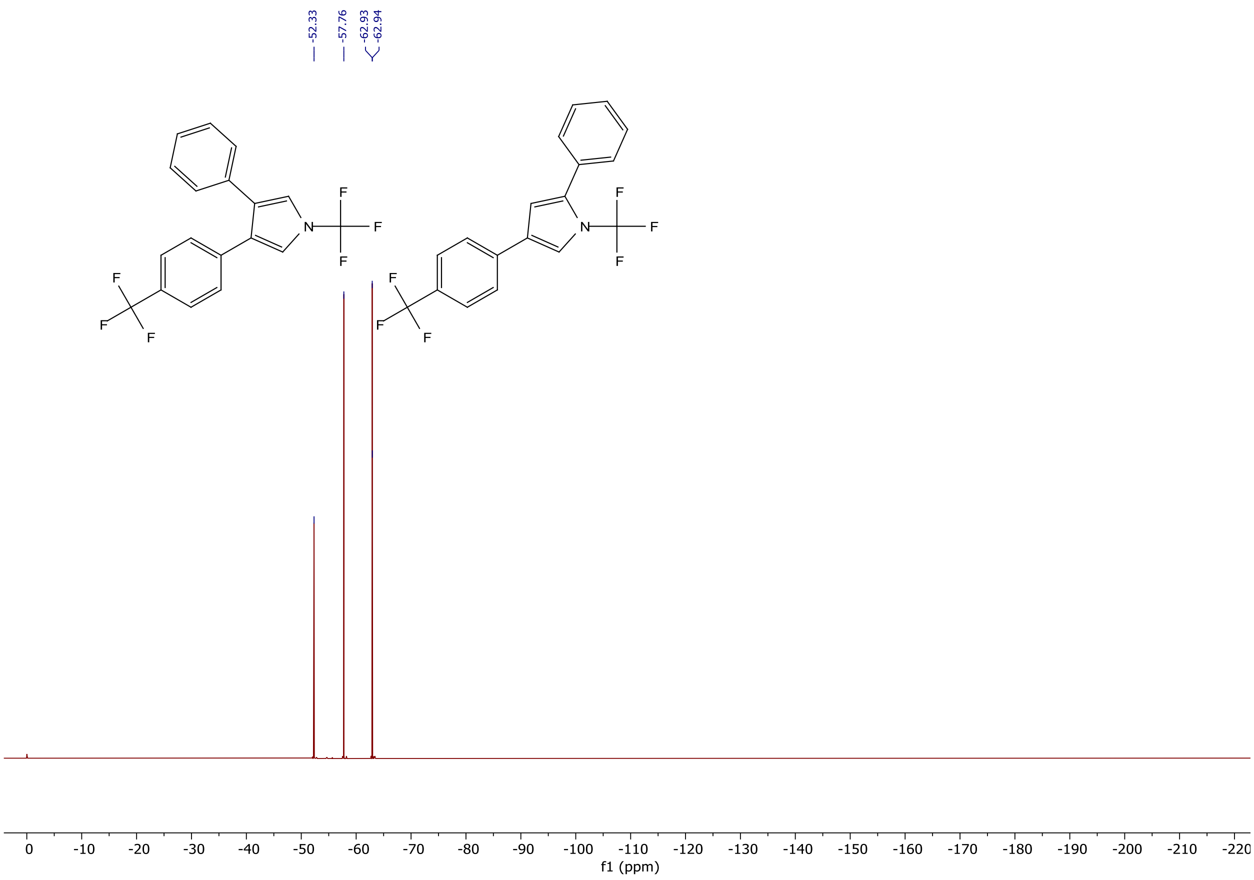

^{1}H NMR (401 MHz, $CDCl_3$) of **2e** + **2e'**

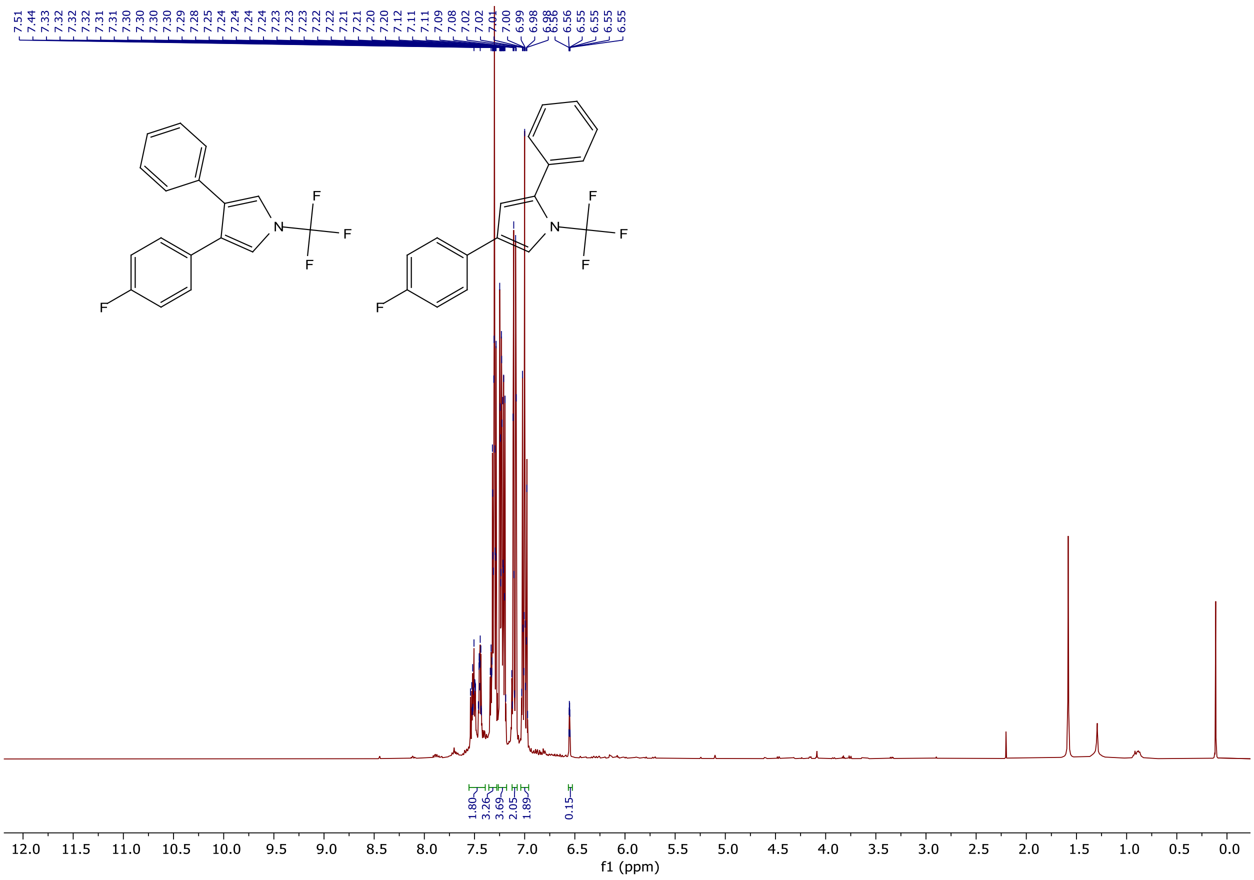

^{13}C NMR (101 MHz, $CDCl_3$) of **2e** + **2e’**

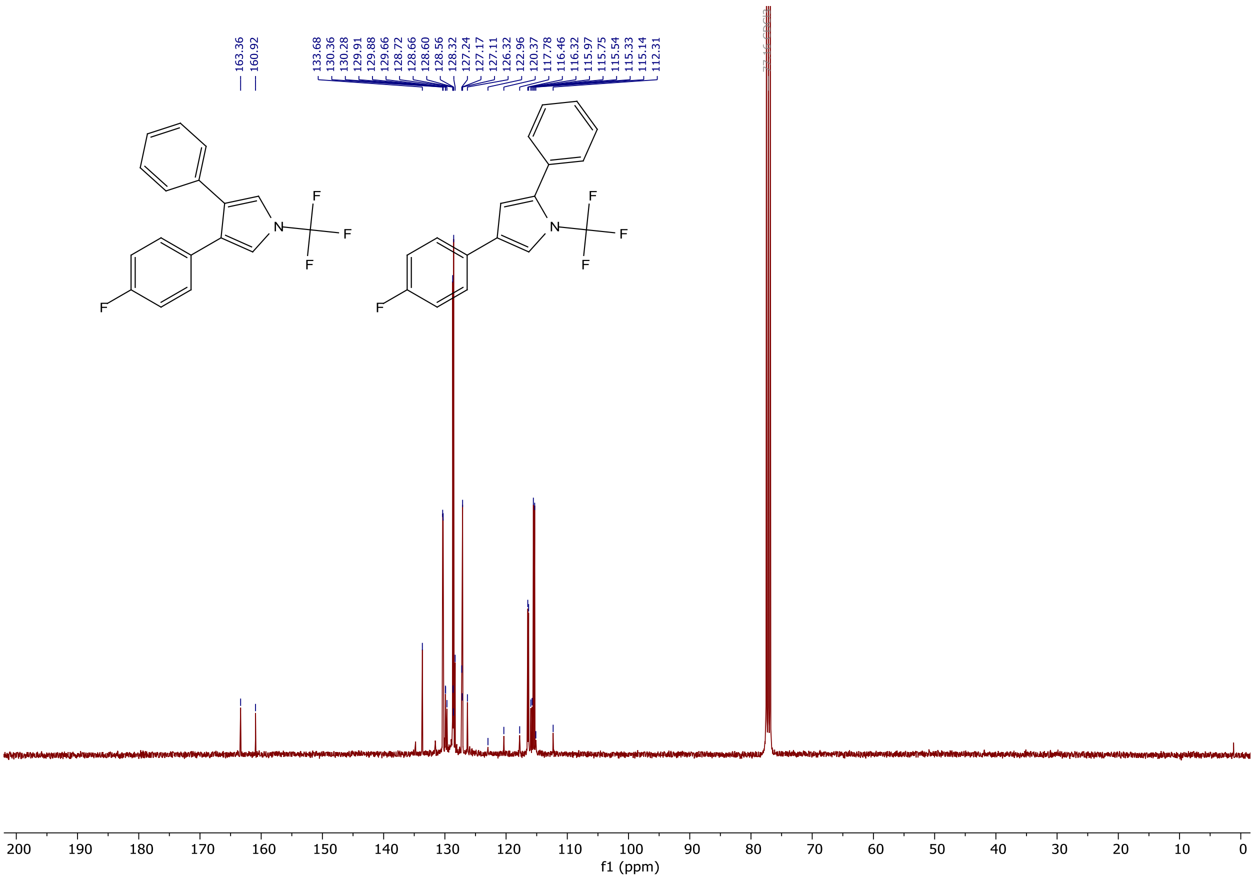

^{19}F NMR (377 MHz, $CDCl_3$) of **2e** + **2e’**

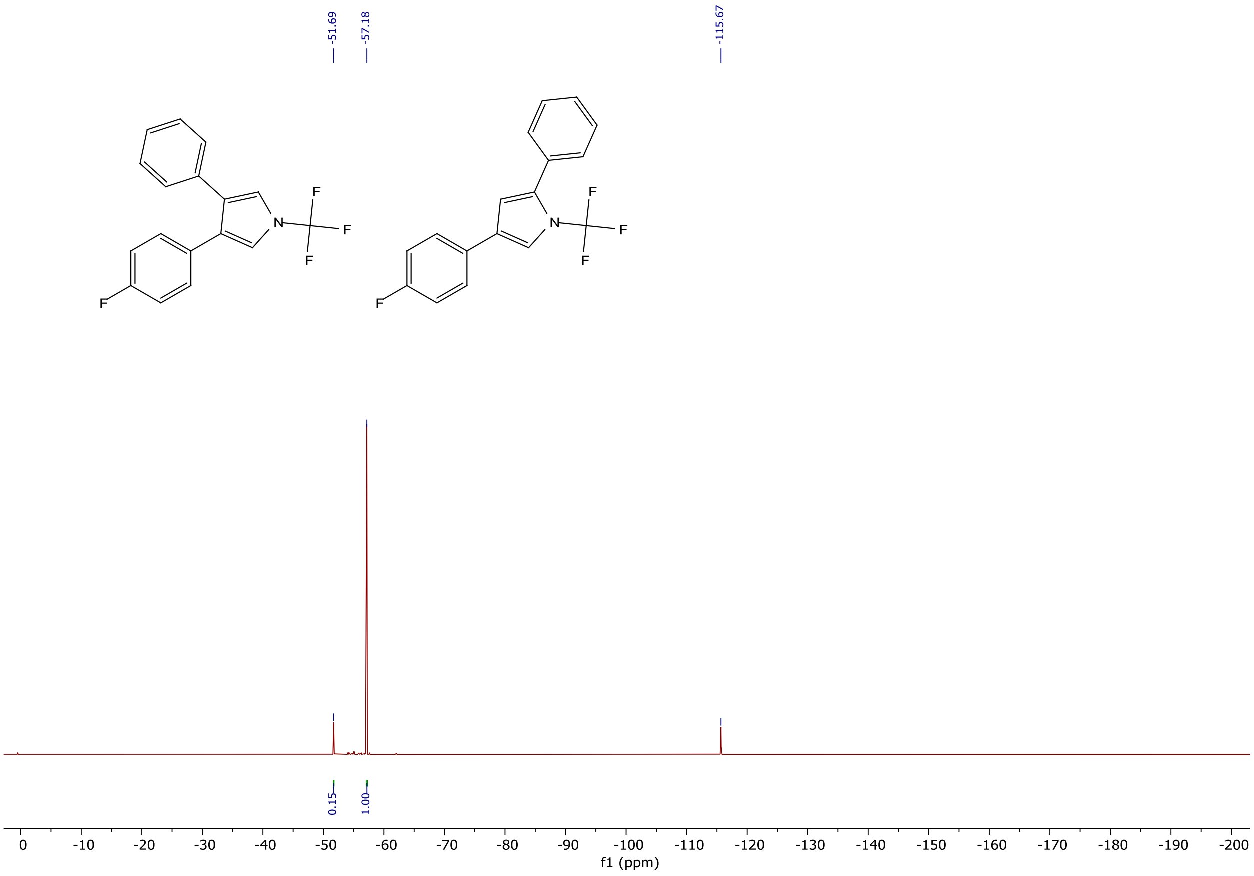

^{1}H NMR (401 MHz, $CDCl_3$) of **2f** + **2f'**

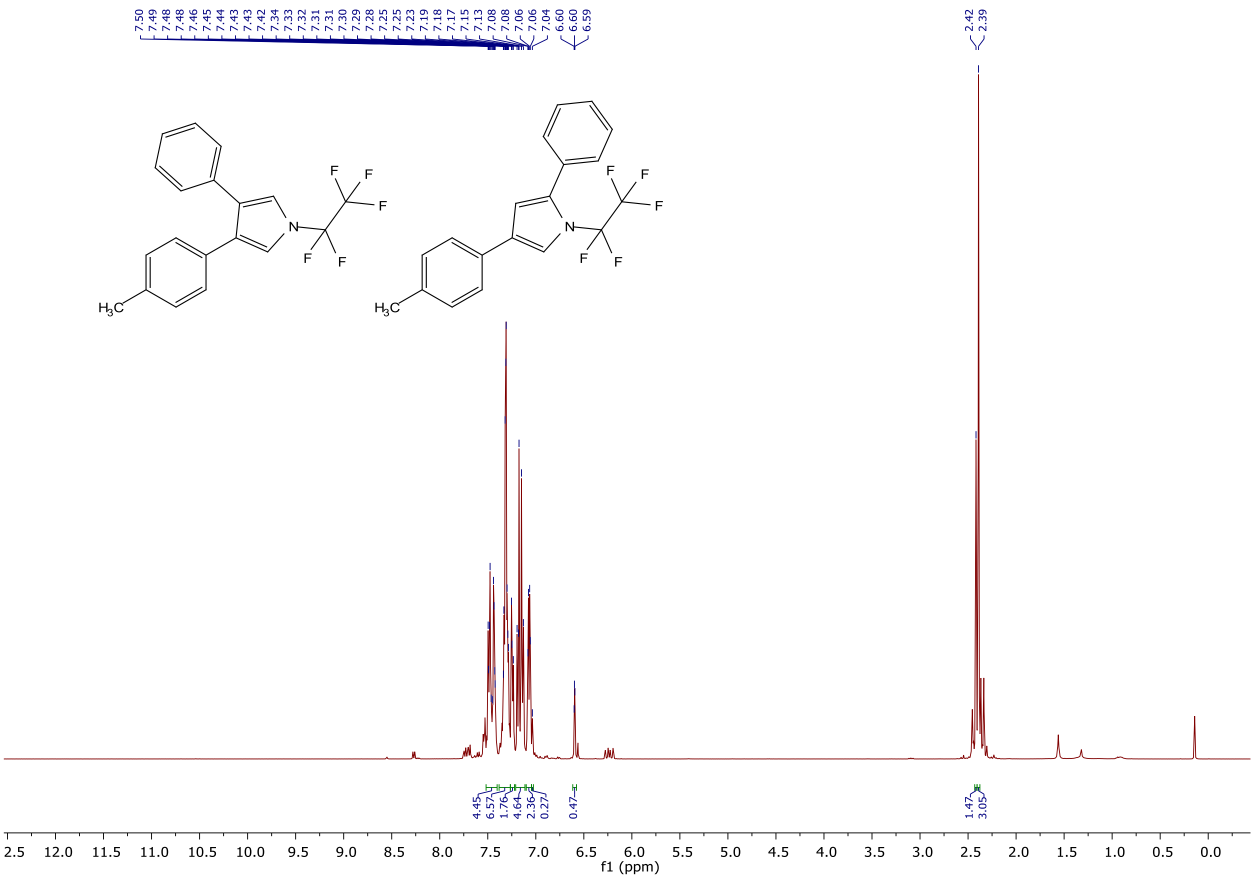

^{13}C NMR (101 MHz, $CDCl_3$) of **2f** + **2f'**

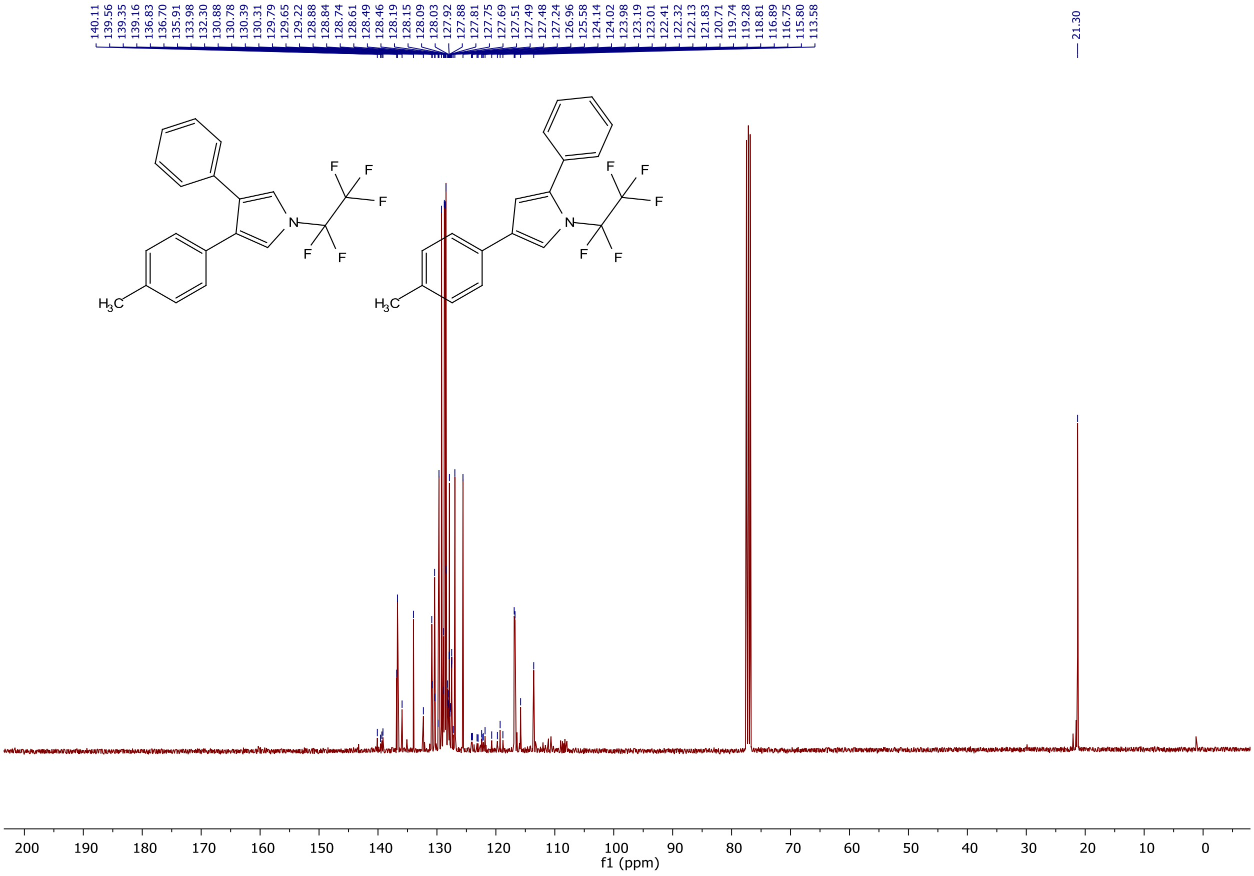

^{19}F NMR (377 MHz, $CDCl_3$) of **2f** + **2f'**

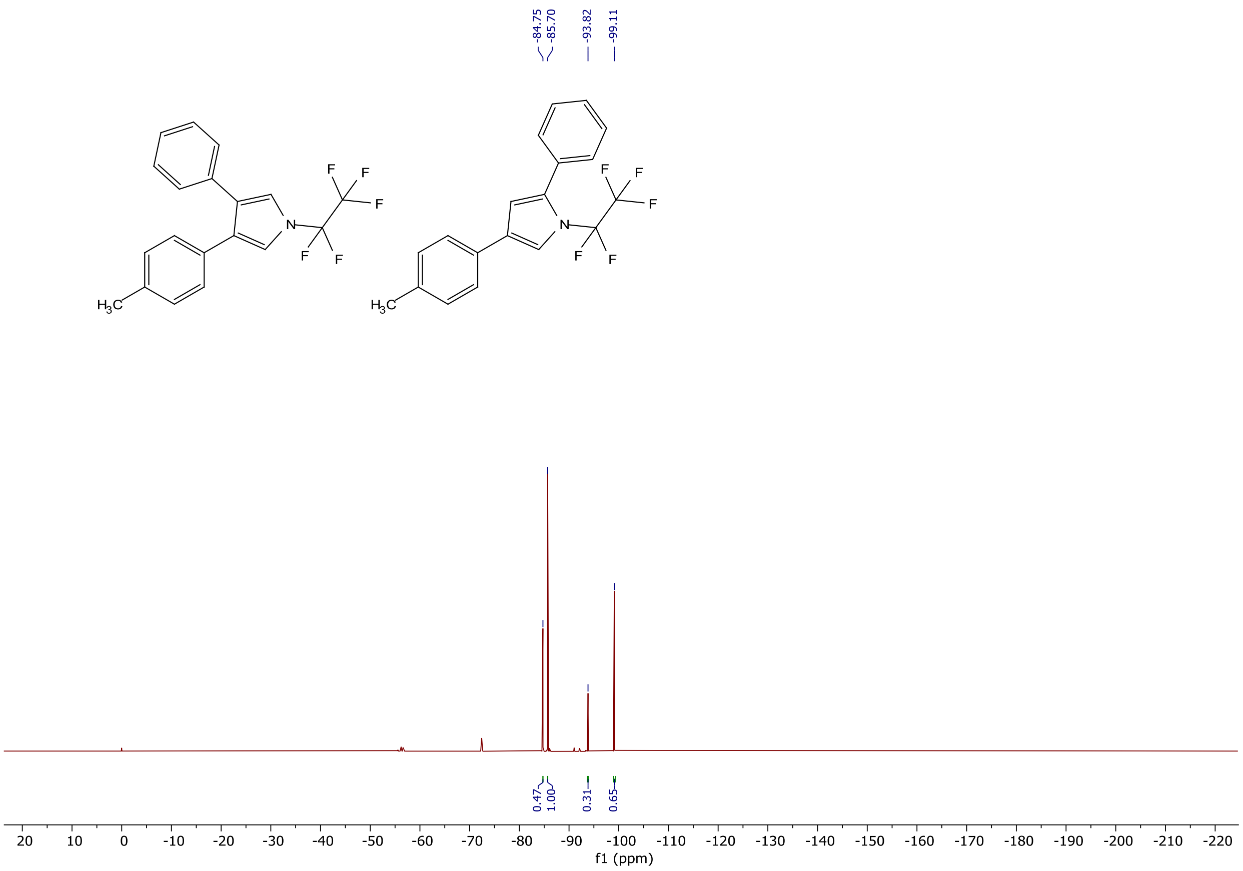

^{1}H NMR (401 MHz, $CDCl_3$) of **3a** + **3a'**

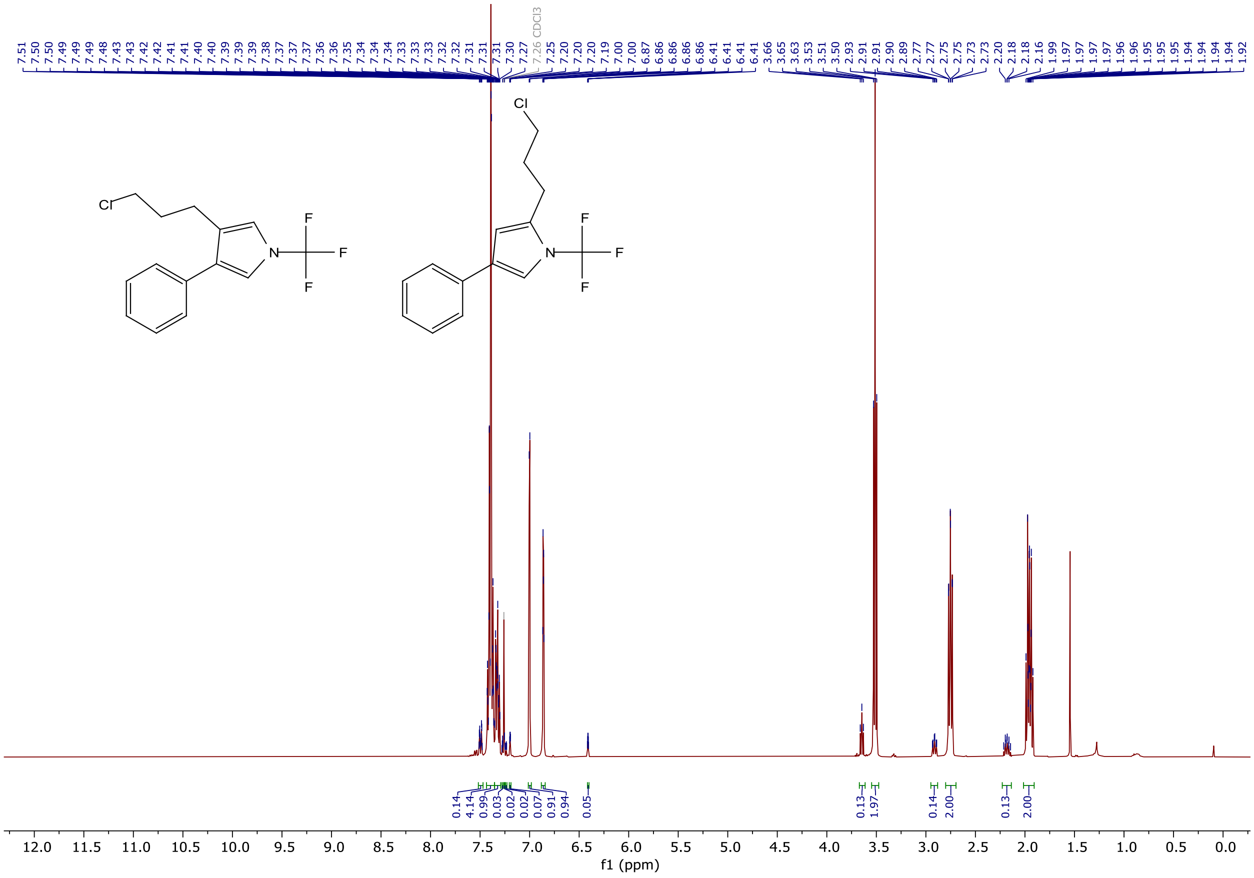

^{13}C NMR (101 MHz, $CDCl_3$) of **3a** + **3a'**

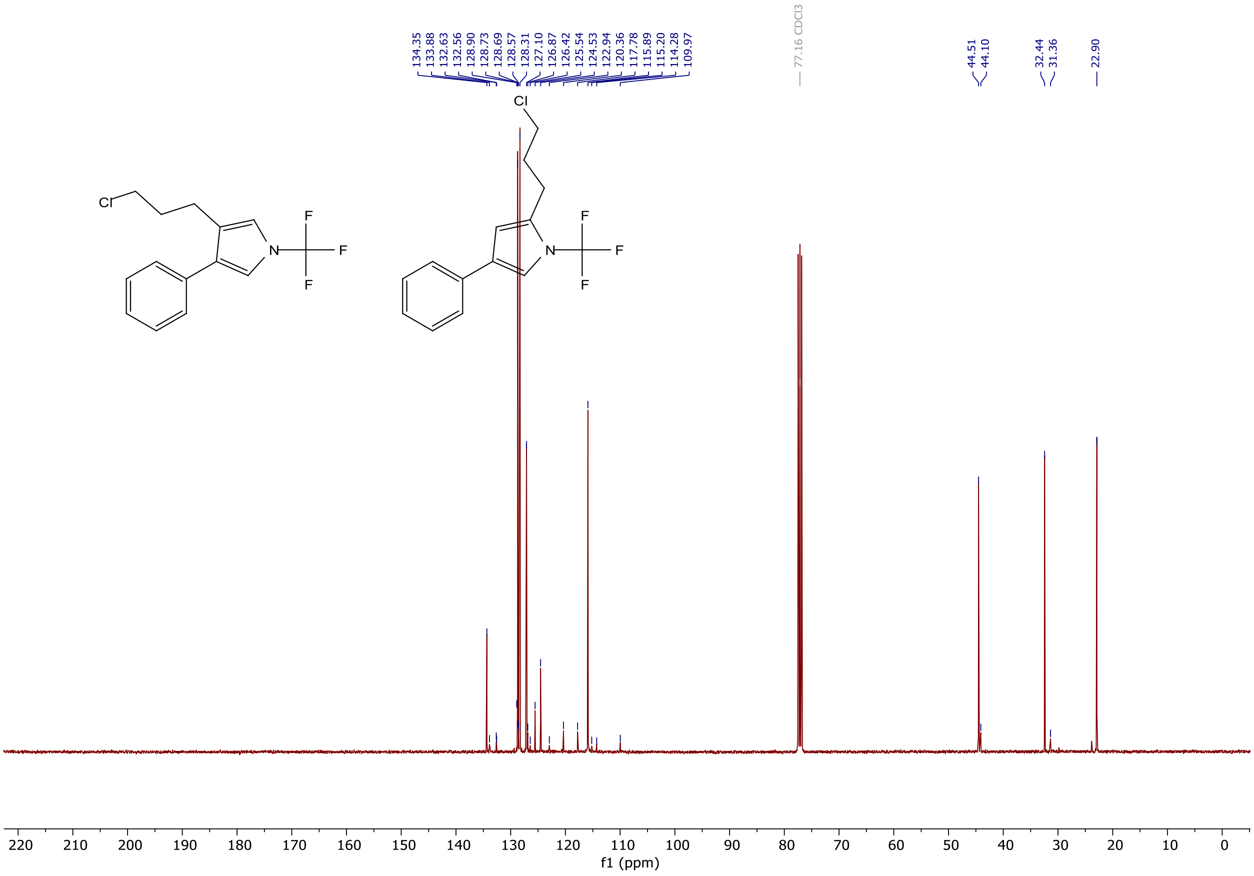

^{19}F NMR (377 MHz, $CDCl_3$) of **3a** + **3a'**

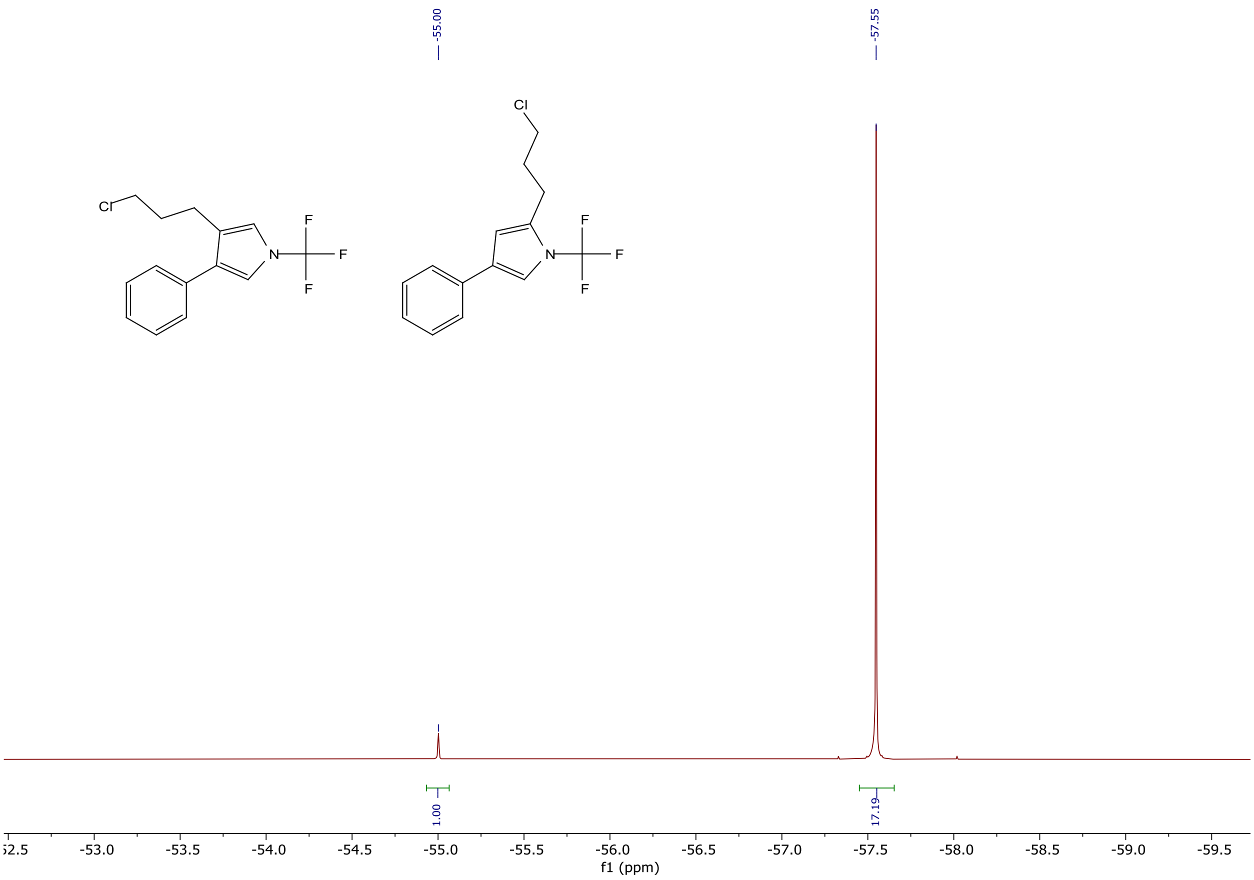

^{1}H NMR (401 MHz, $CDCl_3$) of **3a**

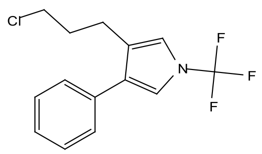

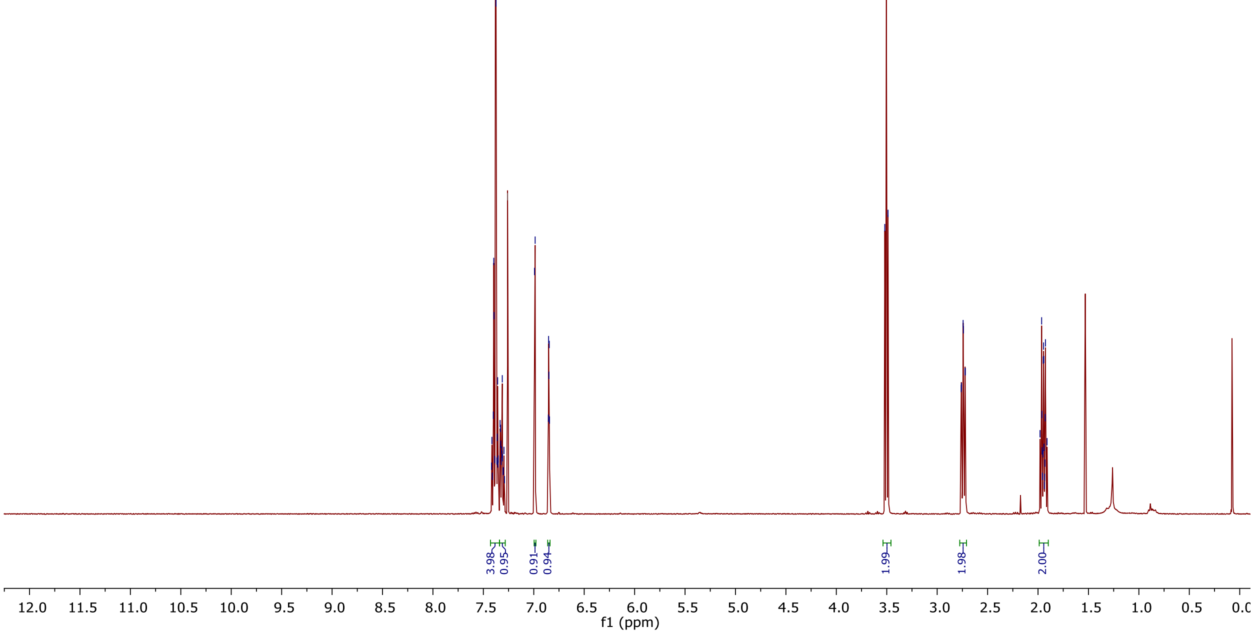

^{13}C NMR (101 MHz, $CDCl_3$) of **3a**

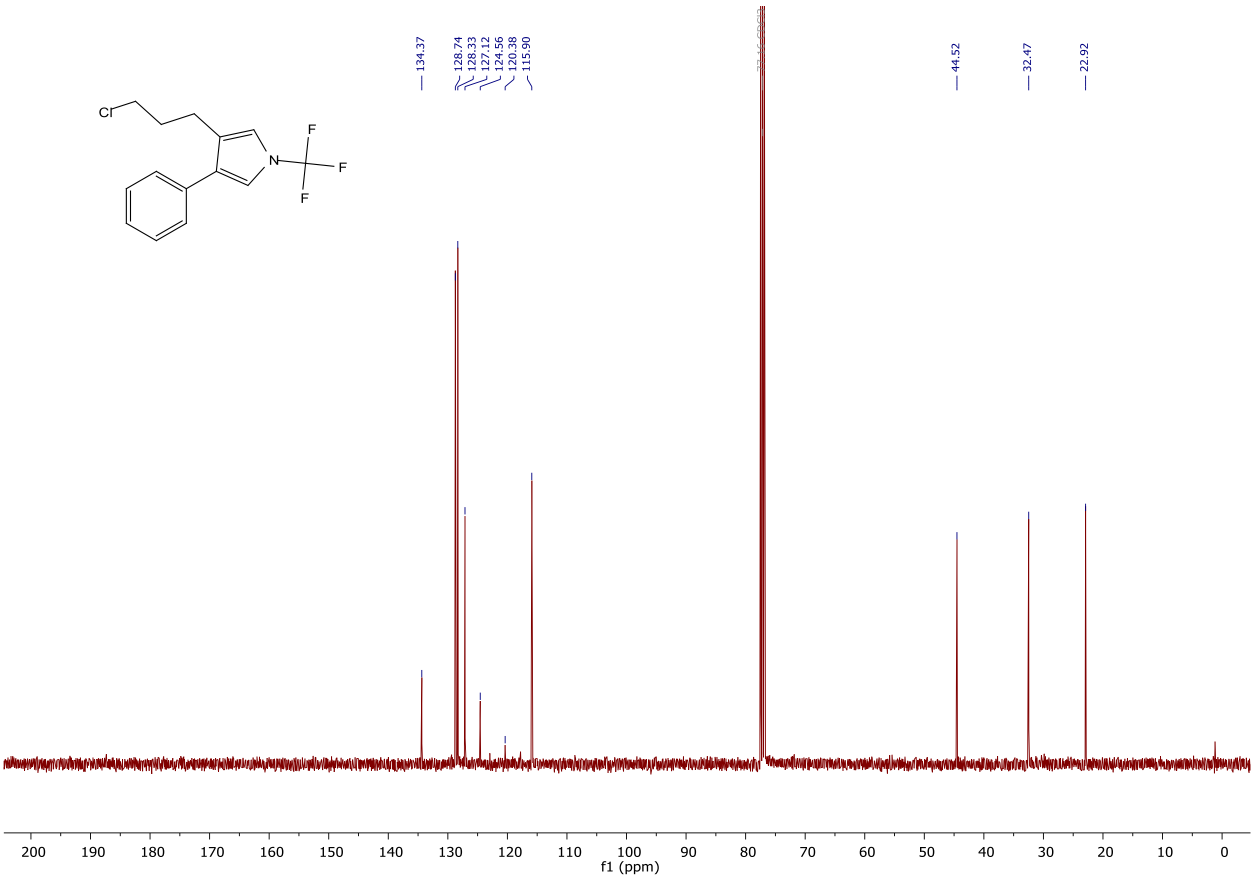

^{19}F NMR (377 MHz, $CDCl_3$) of **3a**

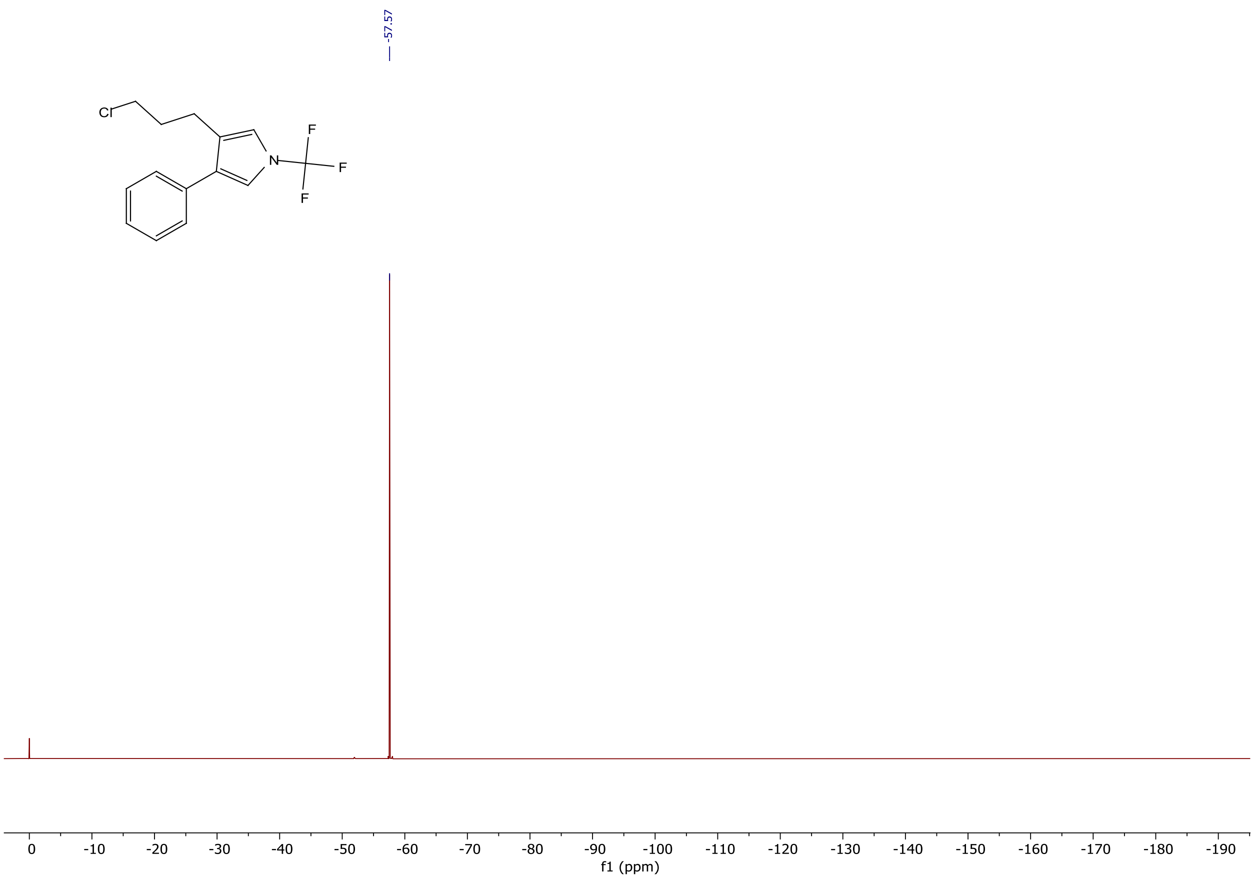

^{1}H NMR (401 MHz, $CDCl_3$) of **3b** + **3b'**

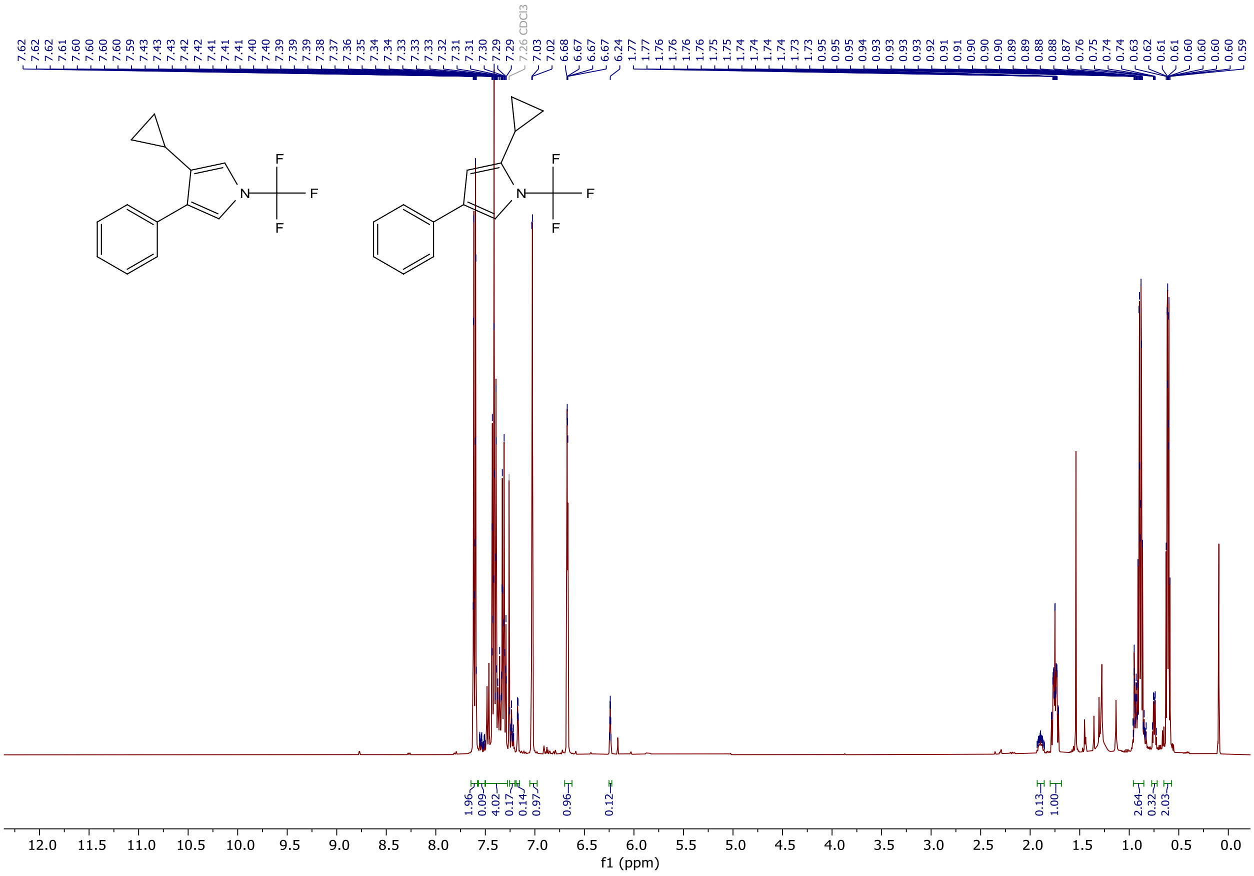

^{13}C NMR (101 MHz, $CDCl_3$) of **3b** + **3b'**

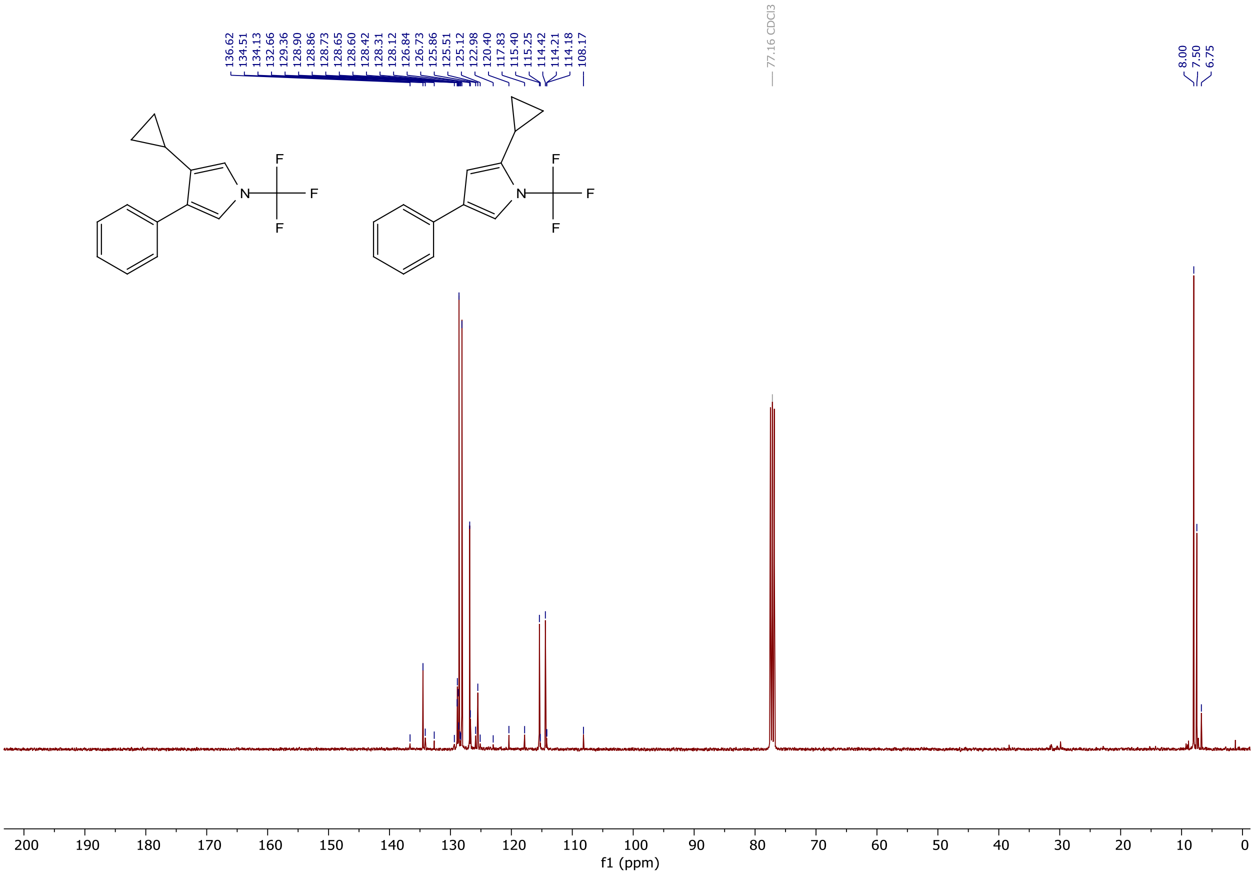

^{19}F NMR (377 MHz, $CDCl_3$) of **3b** + **3b’**

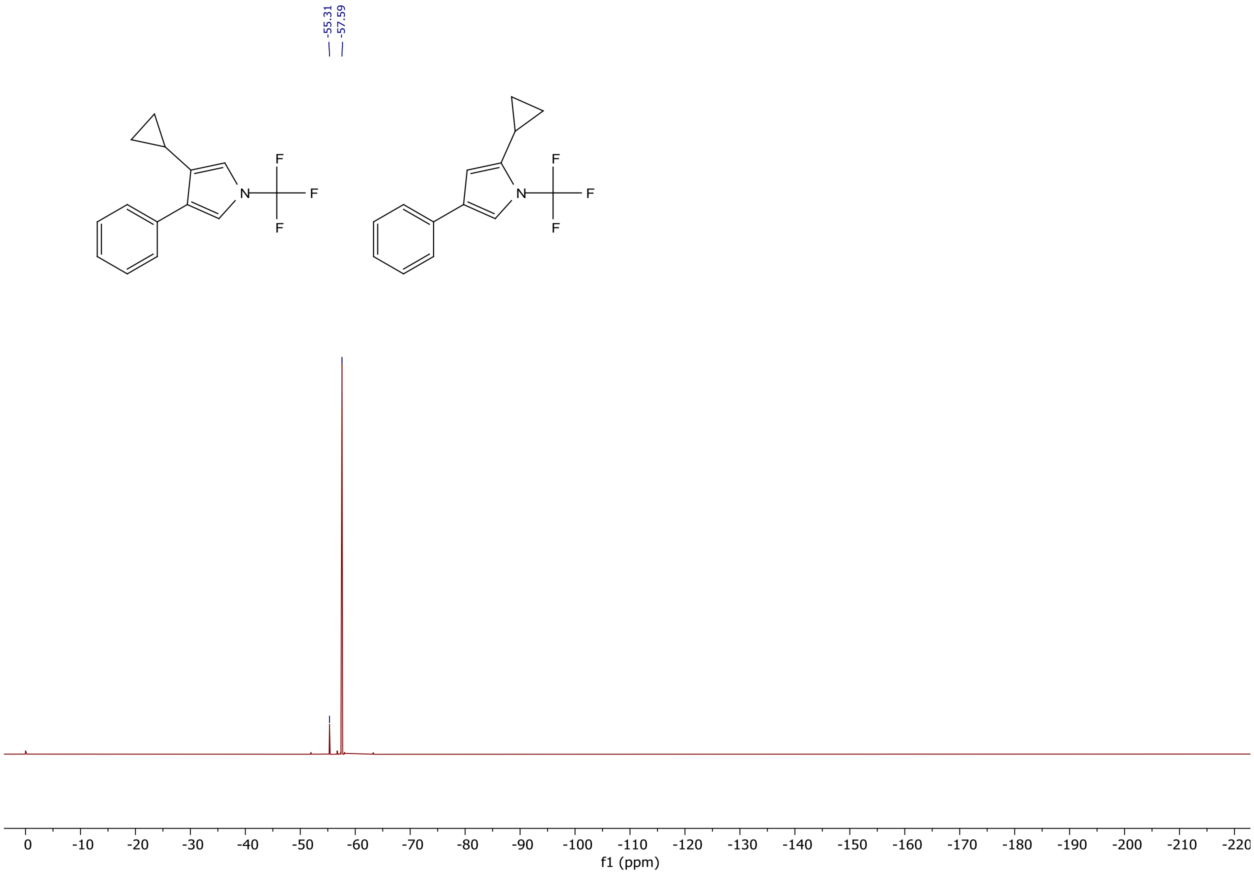

^{1}H NMR (401 MHz, $CDCl_3$) of **3b**

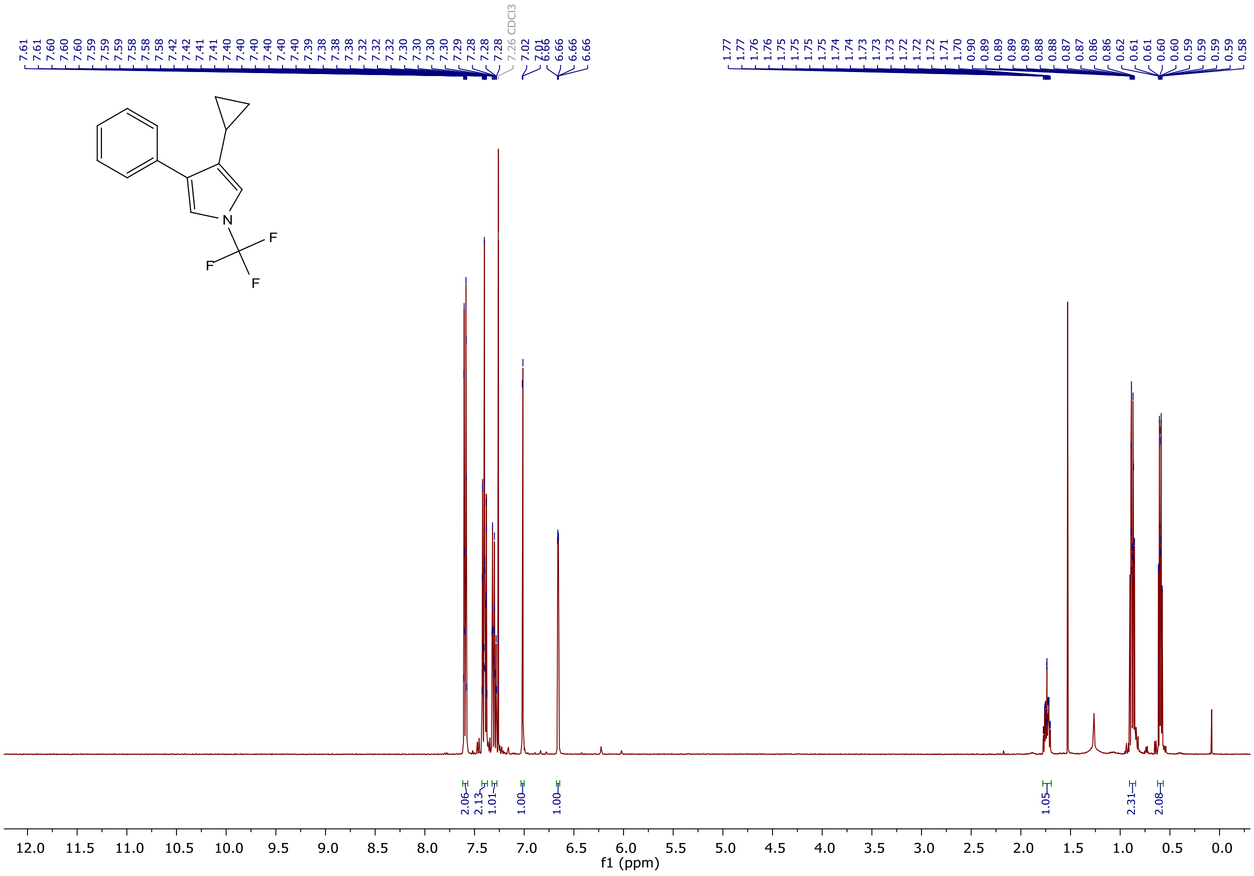

^{13}C NMR (101 MHz, $CDCl_3$) of **3b**

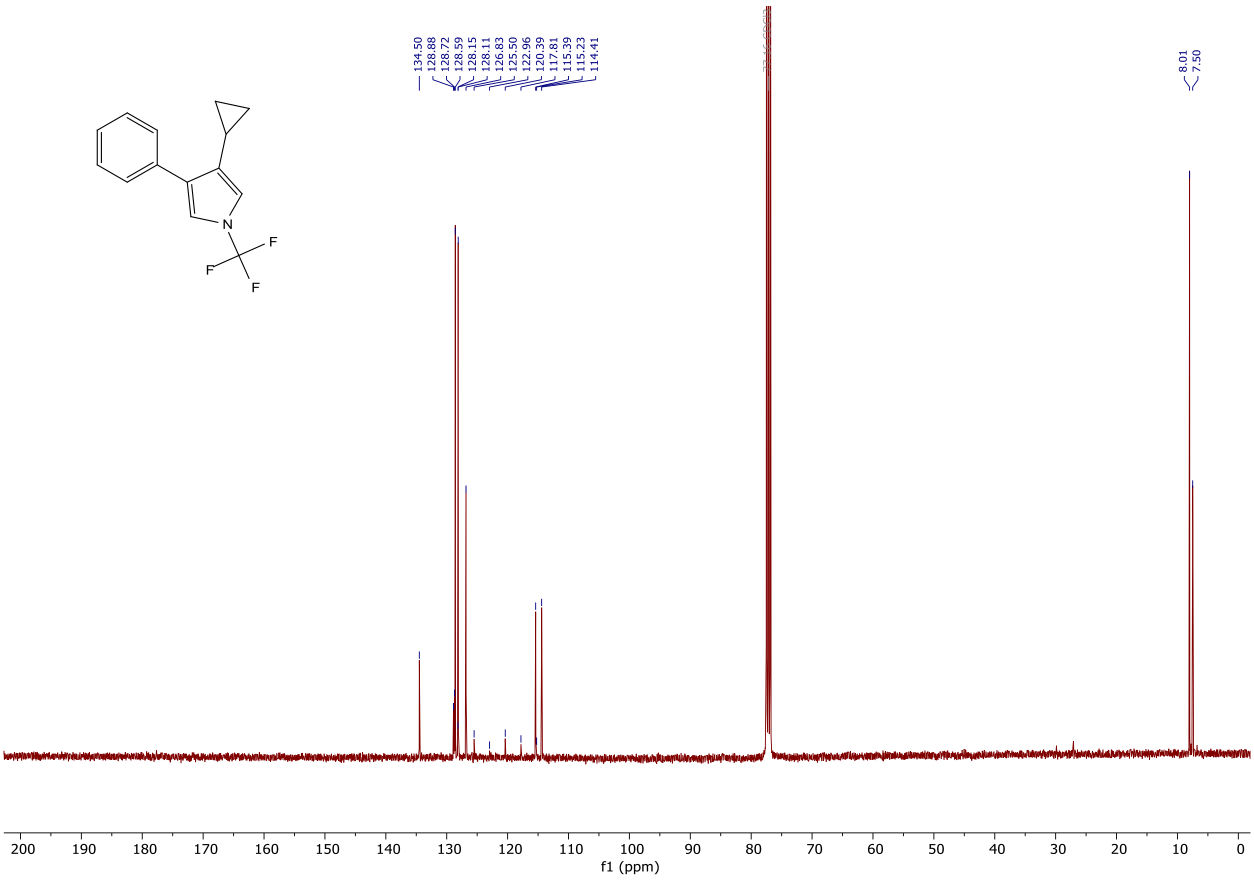

^{19}F NMR (377 MHz, $CDCl_3$) of **3b**

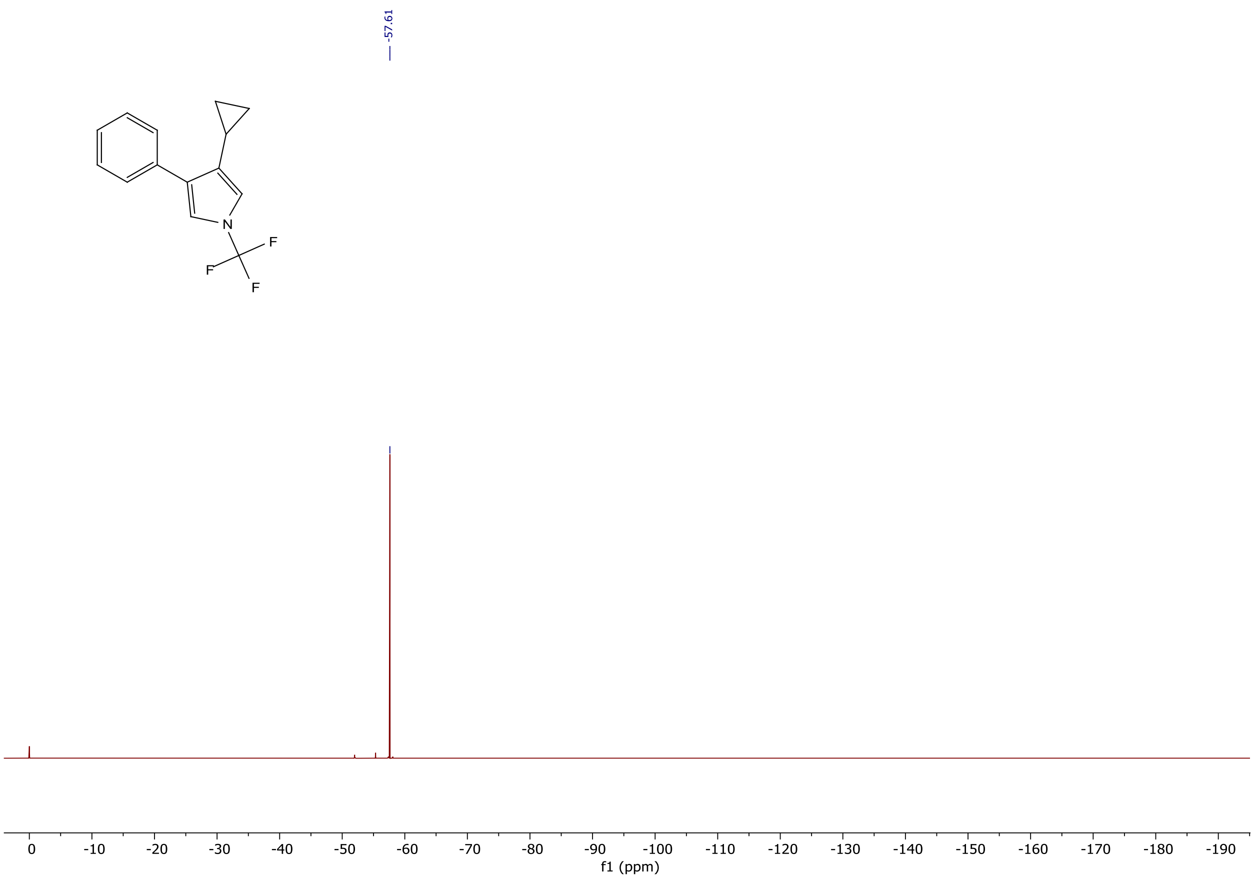

^{1}H NMR (401 MHz, $CDCl_3$) of **3c** + **3c’**

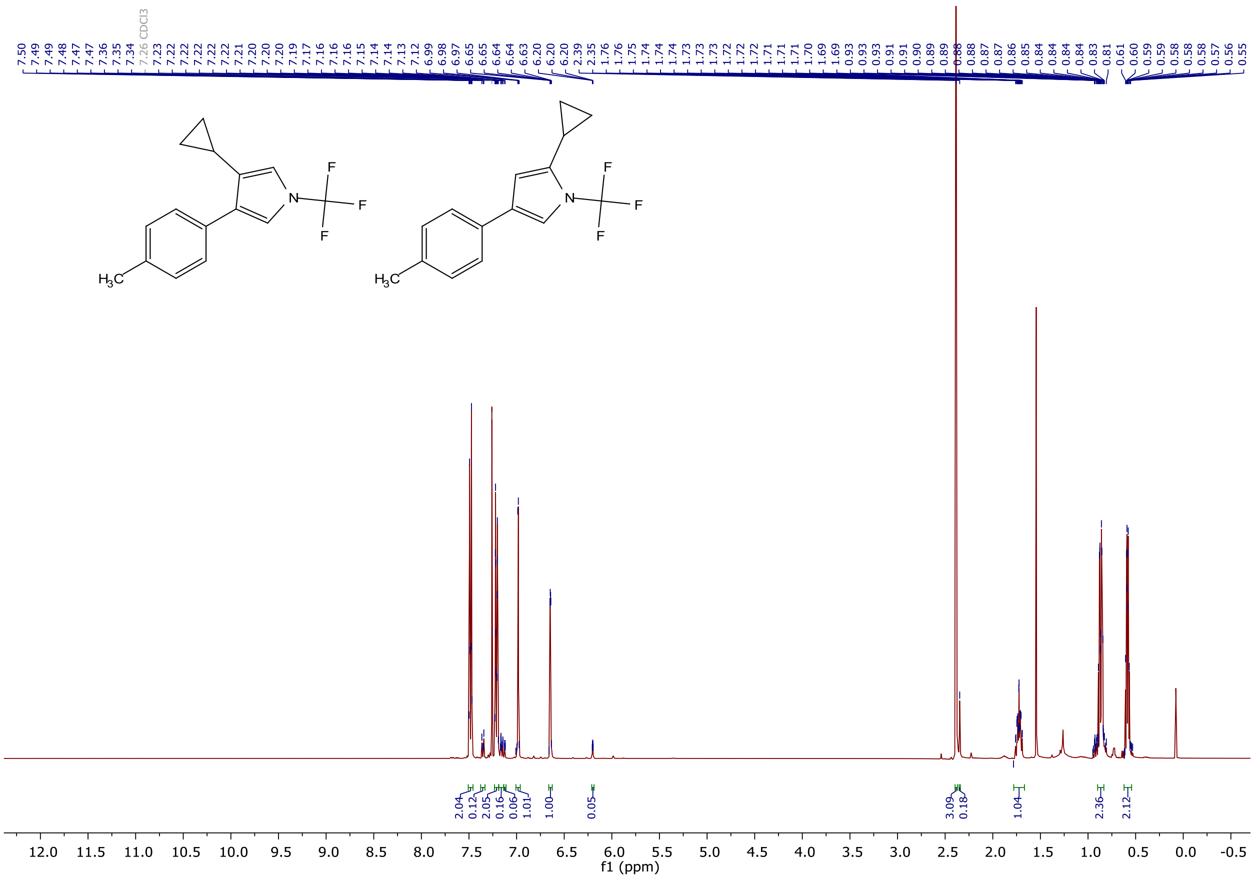

^{13}C NMR (101 MHz, $CDCl_3$) of **3c** + **3c’**

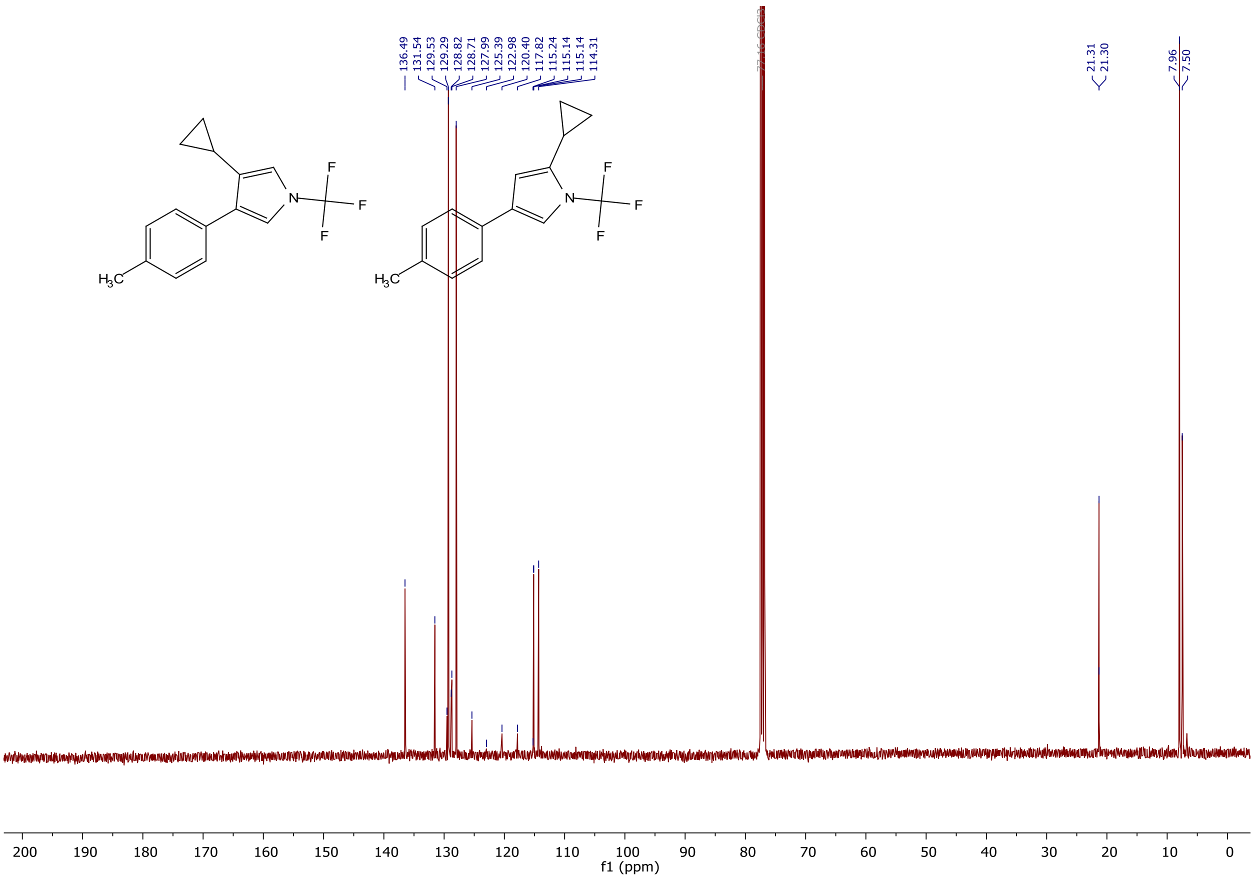

^{19}F NMR (377 MHz, $CDCl_3$) of **3c** + **3c’**

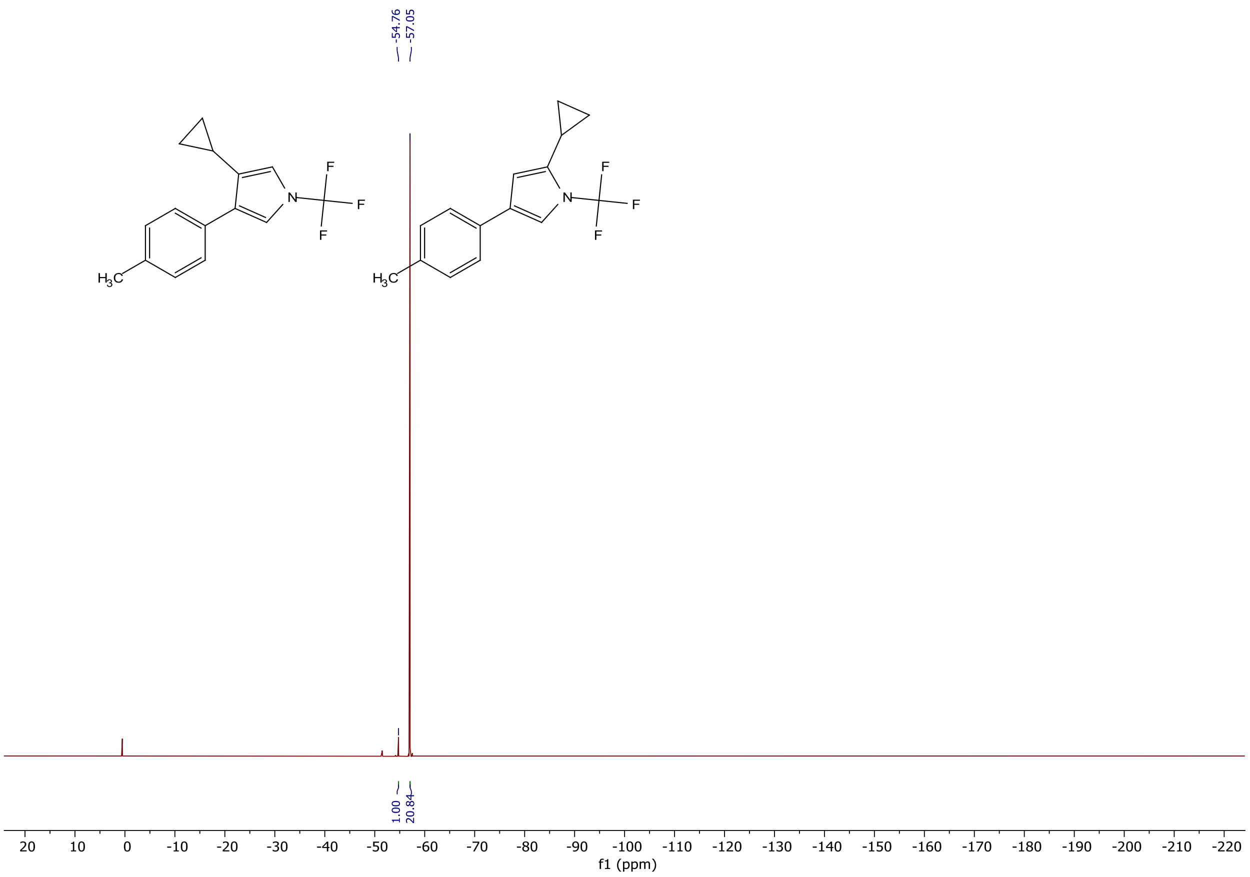

^1H NMR (401 MHz, $CDCl_3$) of **3d** + **3d'**

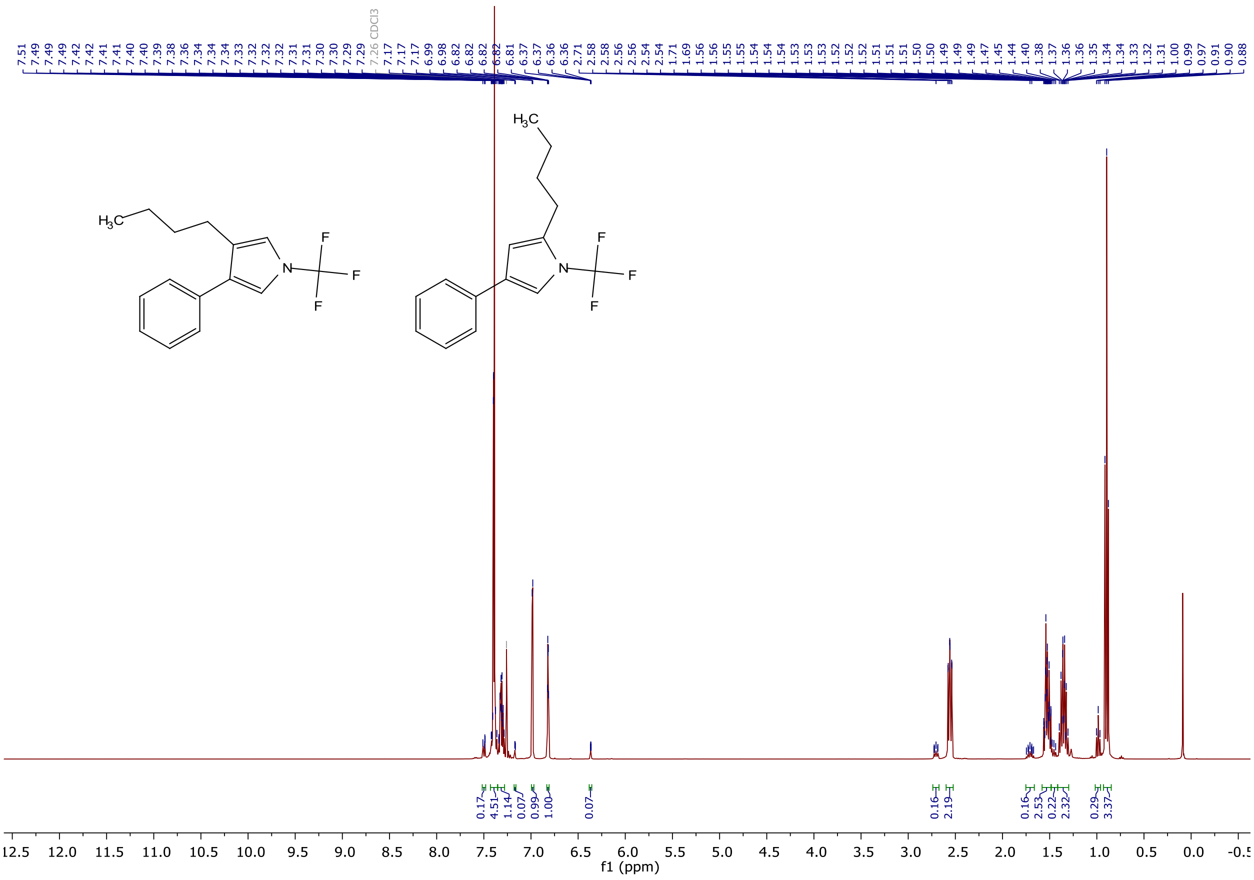

^{13}C NMR (101 MHz, $CDCl_3$) of **3d** + **3d'**

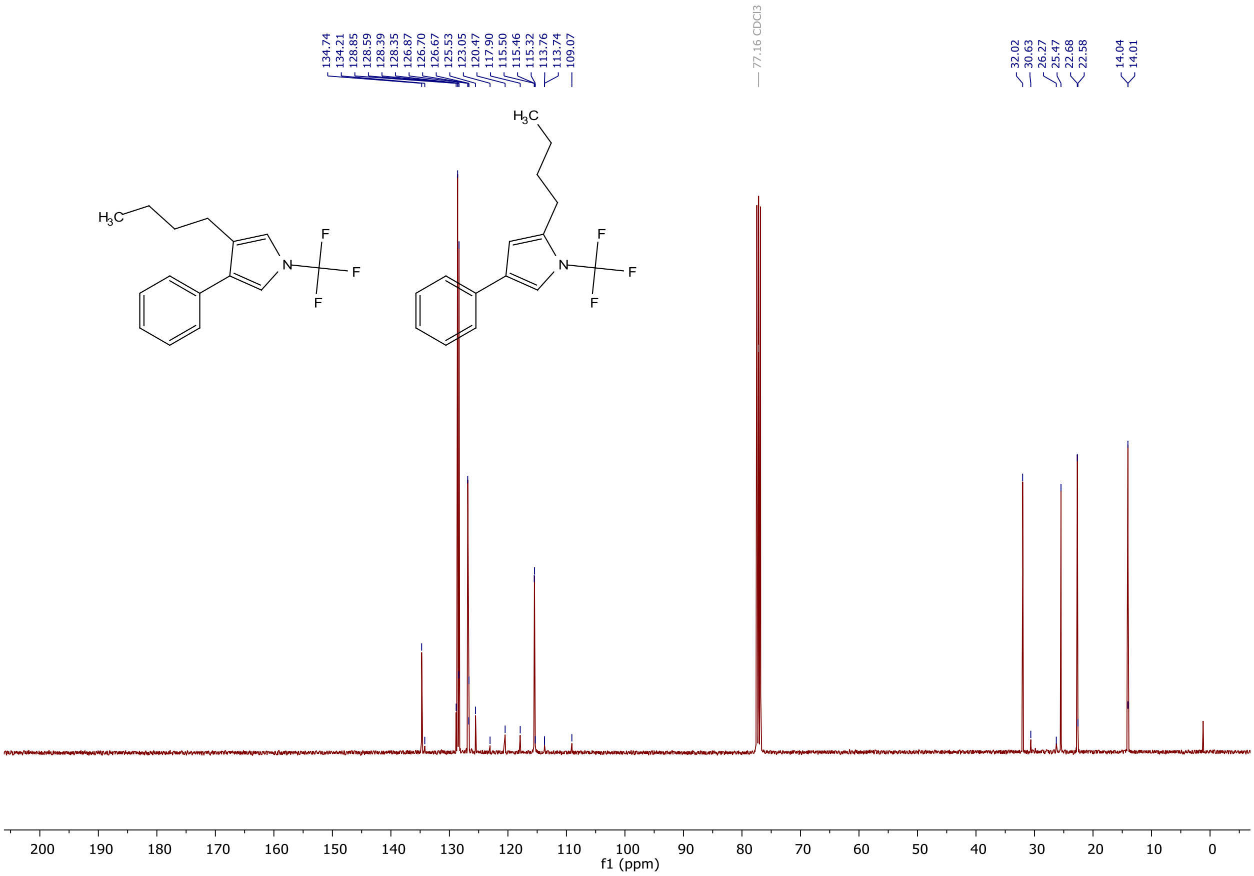

^{19}F NMR (377 MHz, $CDCl_3$) of **3d** + **3d’**

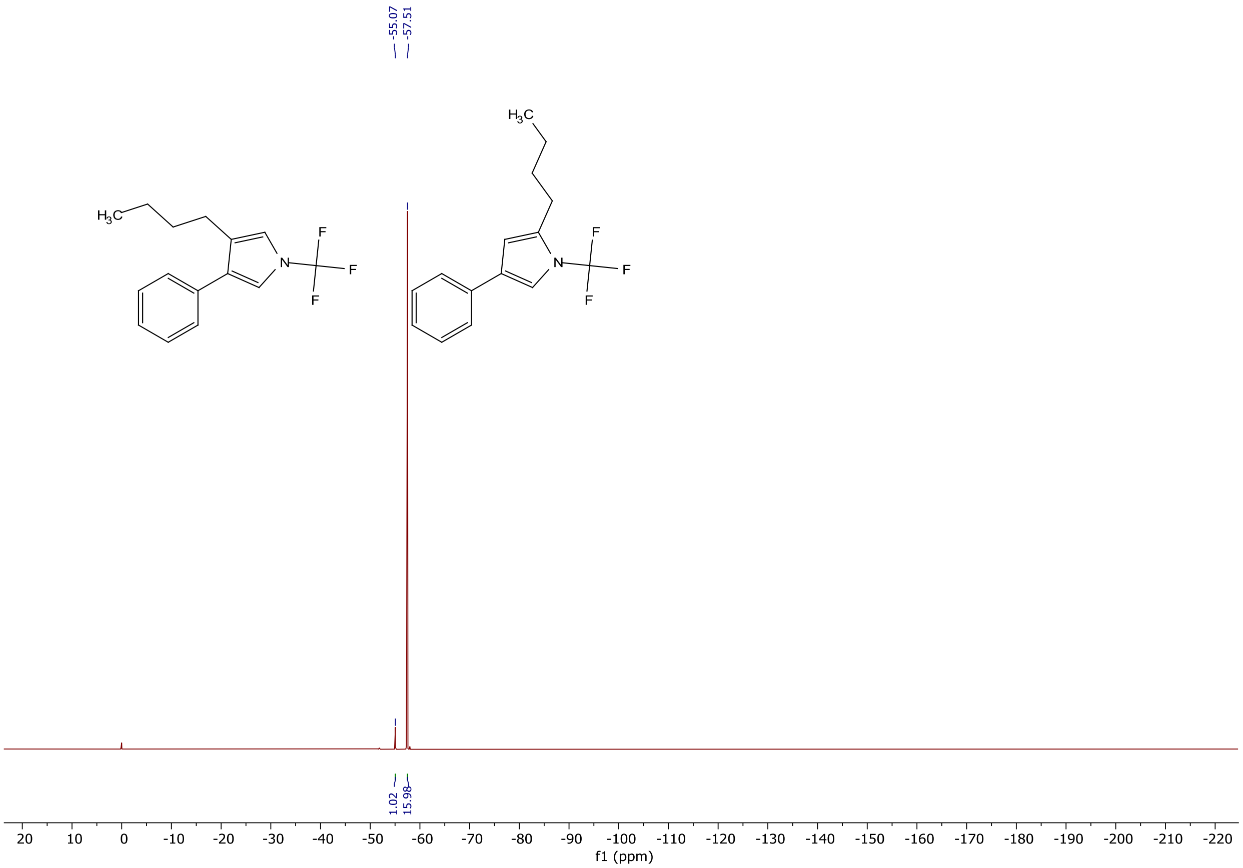

^{1}H NMR (401 MHz, $CDCl_3$) of **3d**

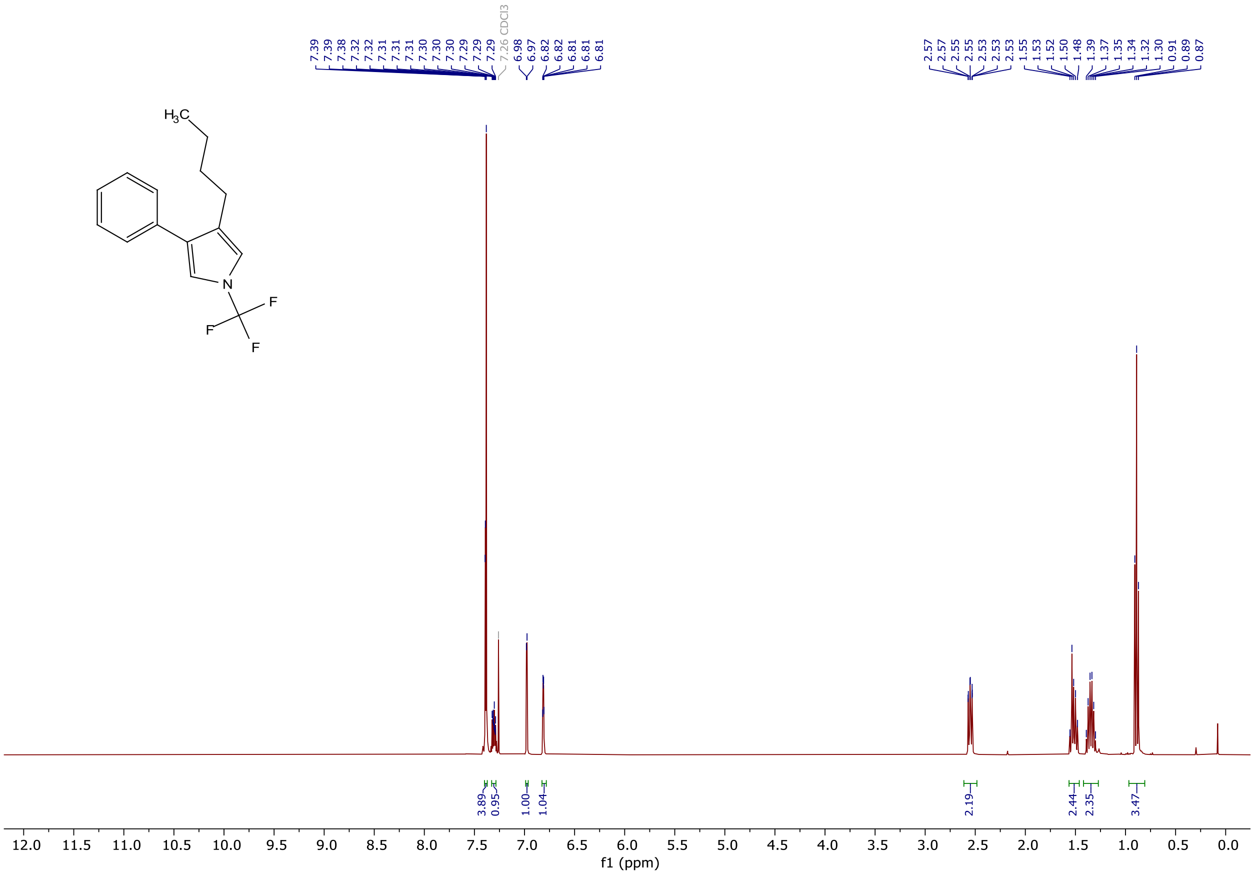

^{13}C NMR (101 MHz, $CDCl_3$) of **3d**

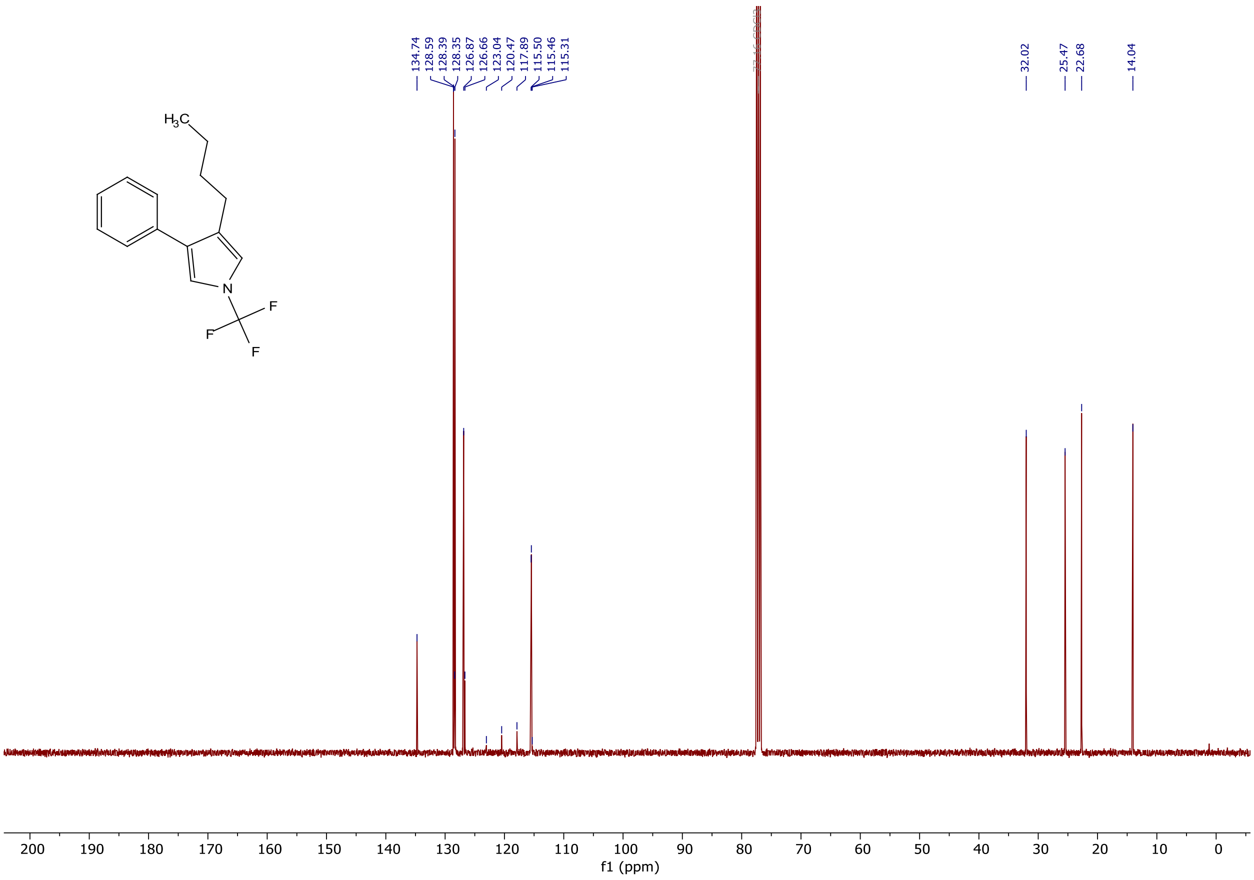

^{19}F NMR (377 MHz, $CDCl_3$) of **3d**

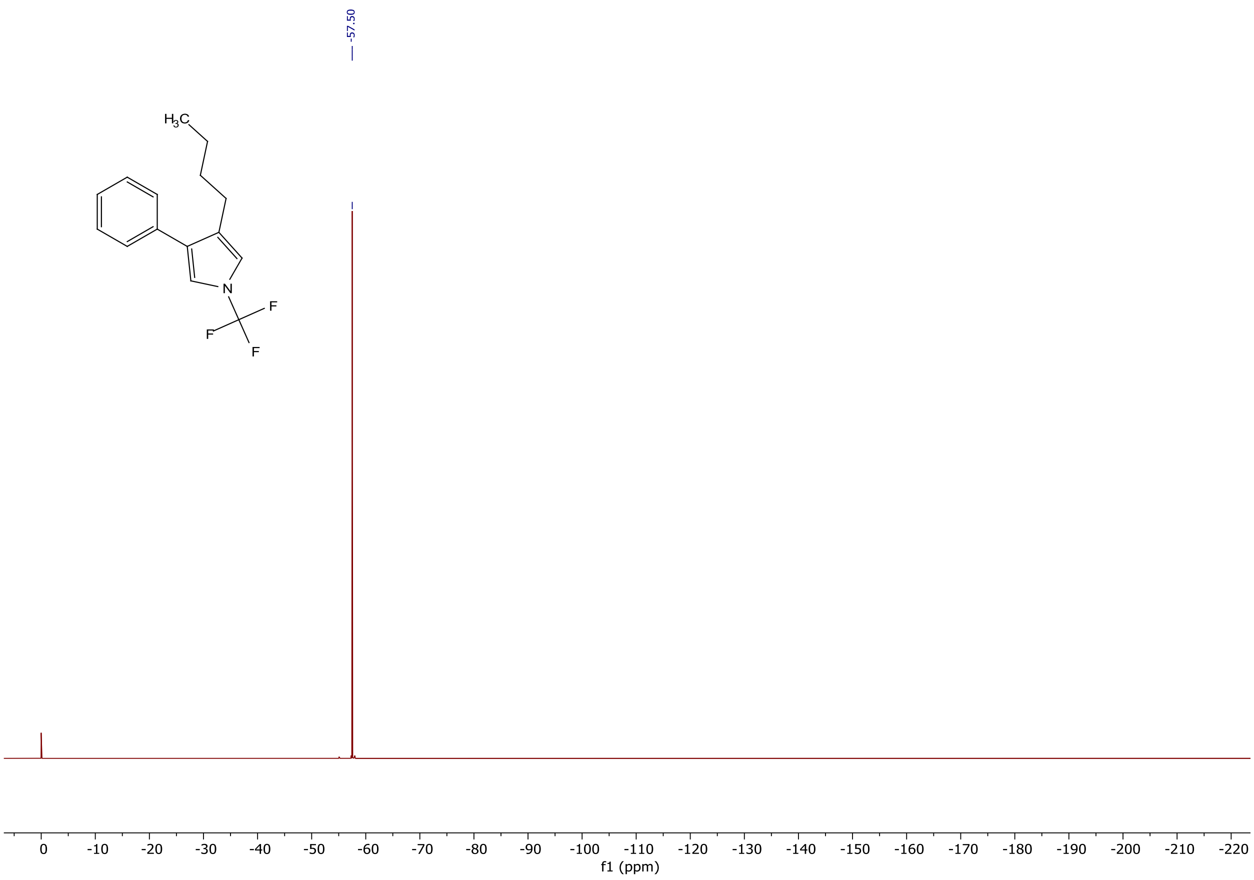

^{1}H NMR (401 MHz, $CDCl_3$) of **3e** + **3e'**

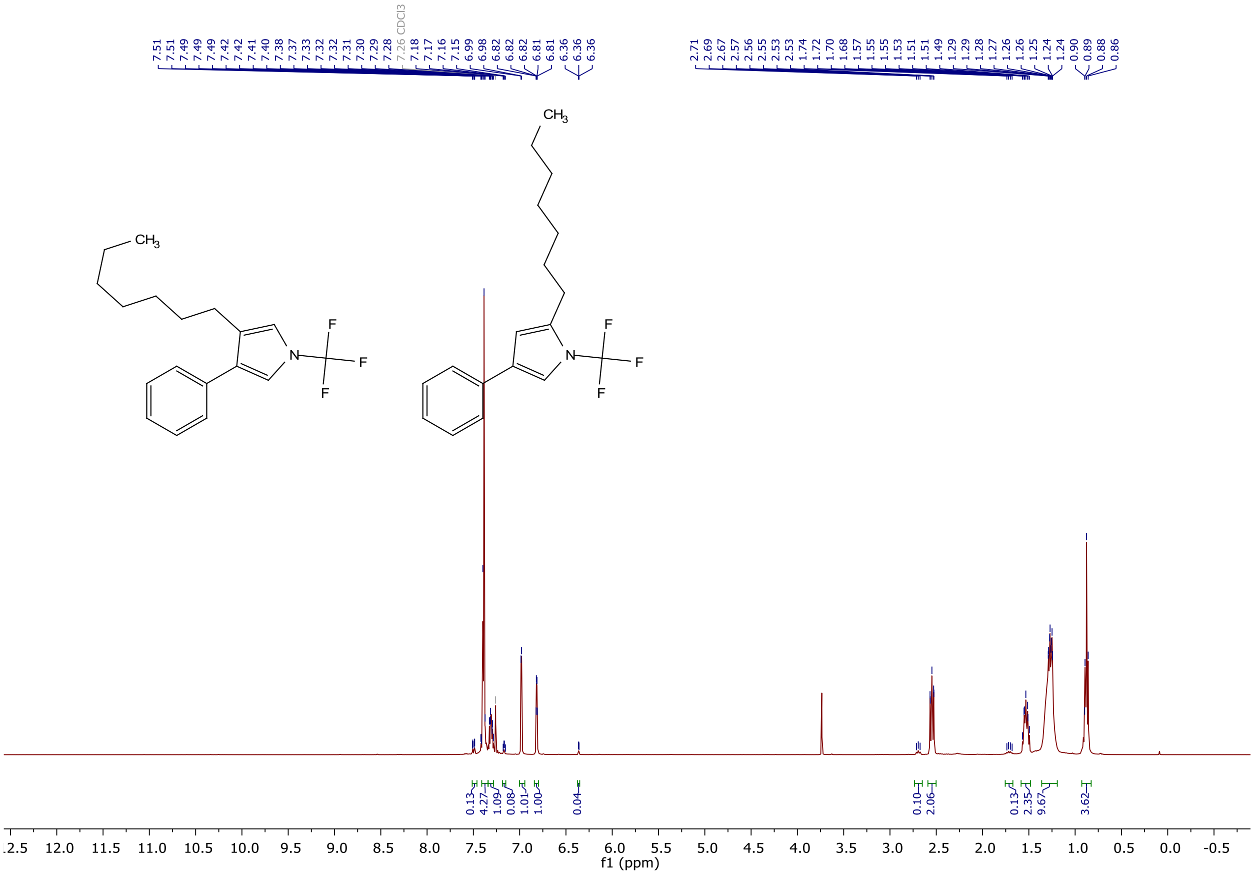

^{13}C NMR (101 MHz, $CDCl_3$) of **3e** + **3e'**

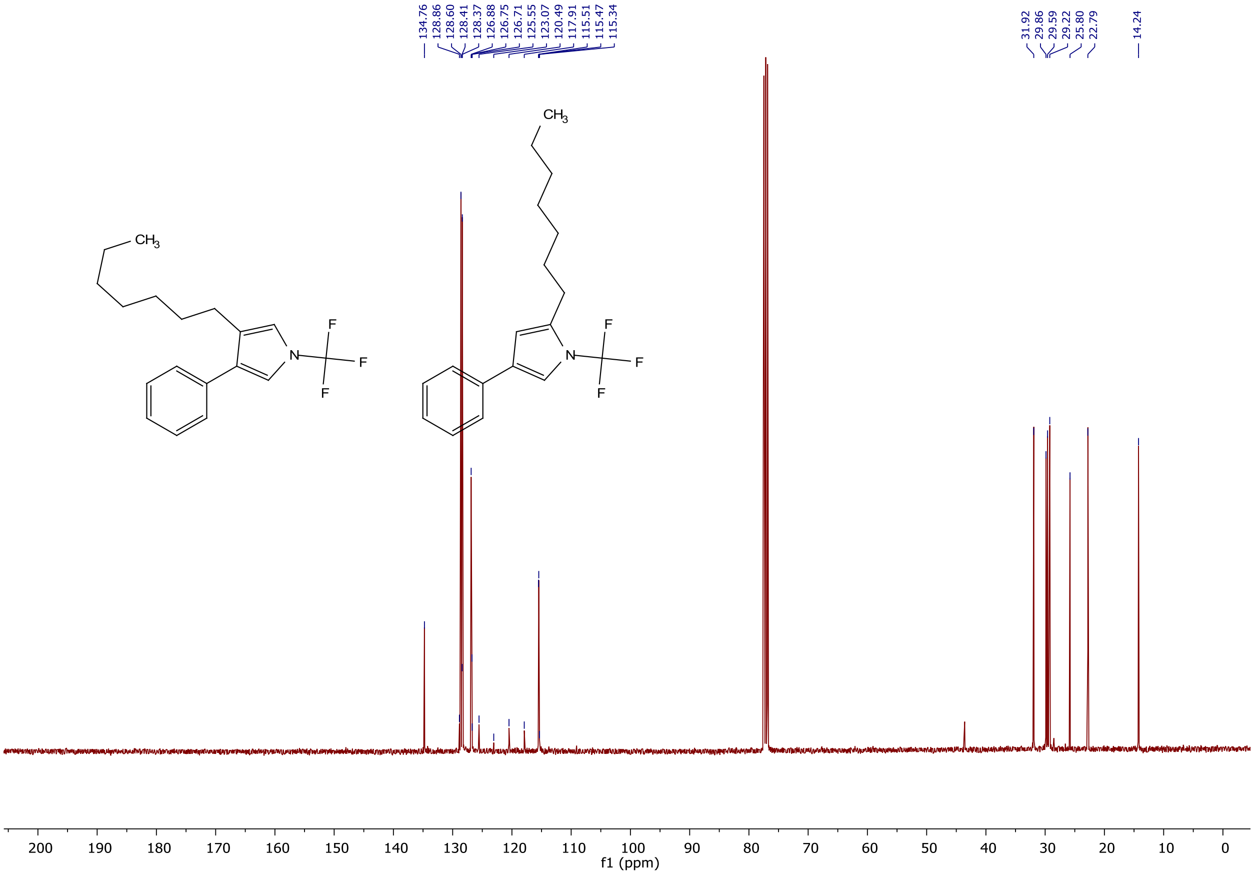

^{19}F NMR (377 MHz, $CDCl_3$) of **3e** + **3e'**

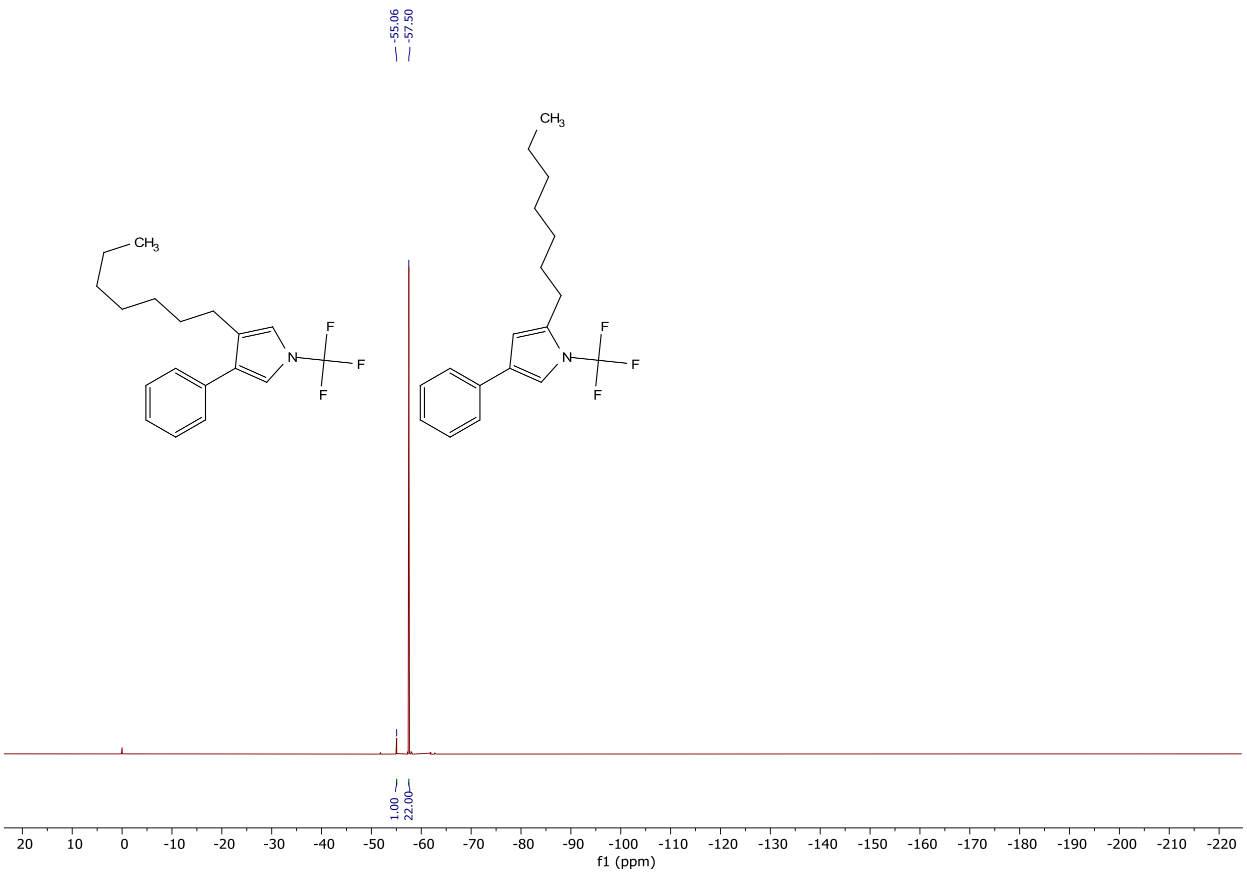

^{1}H NMR (401 MHz, $CDCl_3$) of **3f** + **3f’**

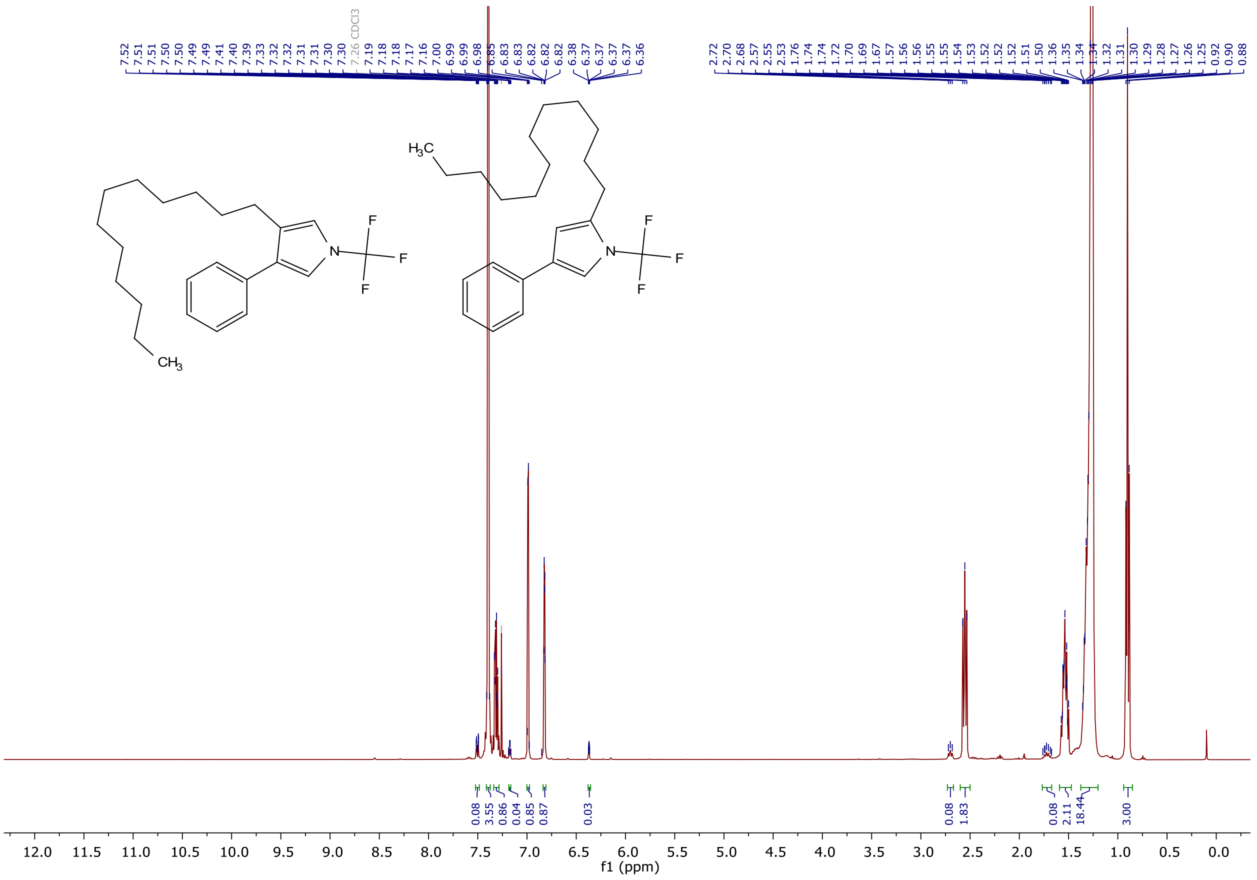

^{13}C NMR (101 MHz, $CDCl_3$) of **3f** + **3f’**

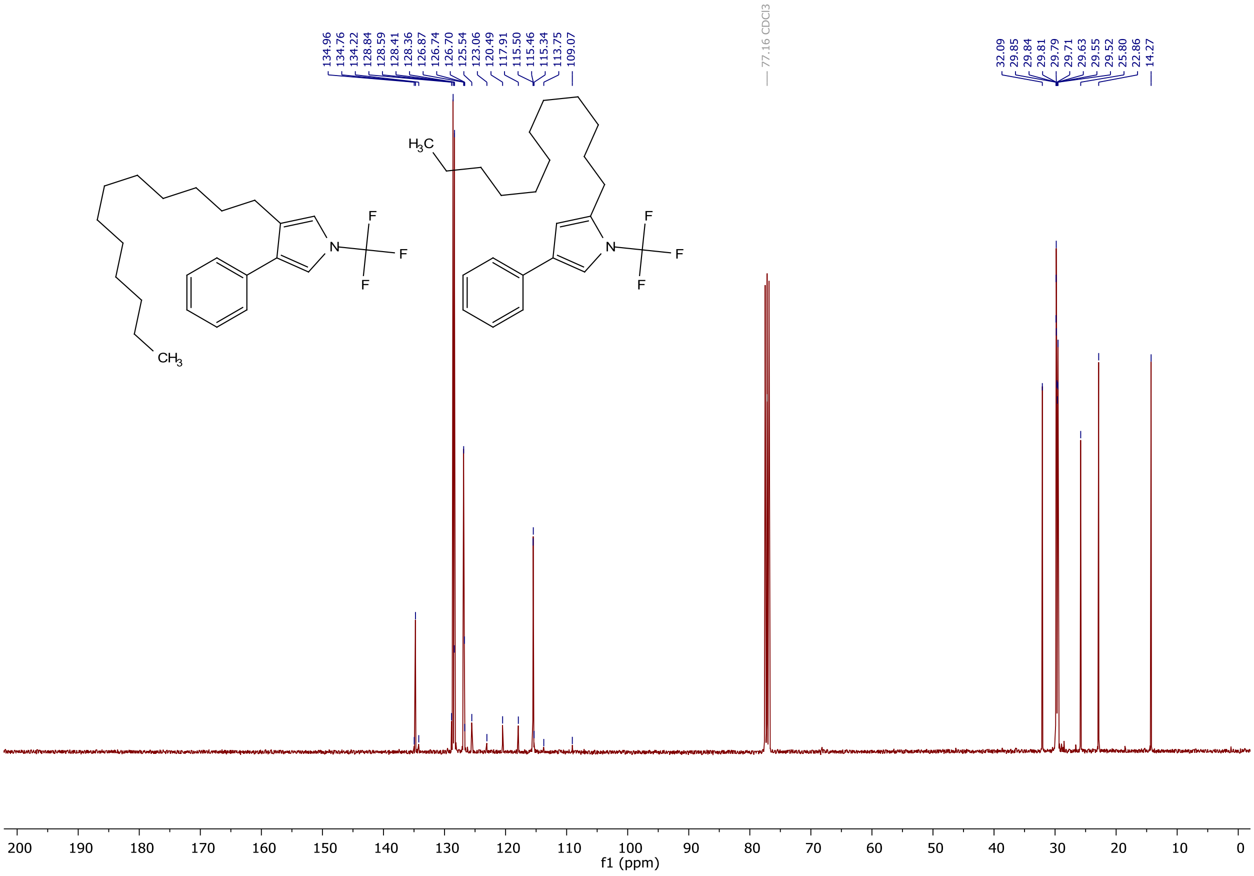

^{19}F NMR (377 MHz, $CDCl_3$) of **3f** + **3f'**

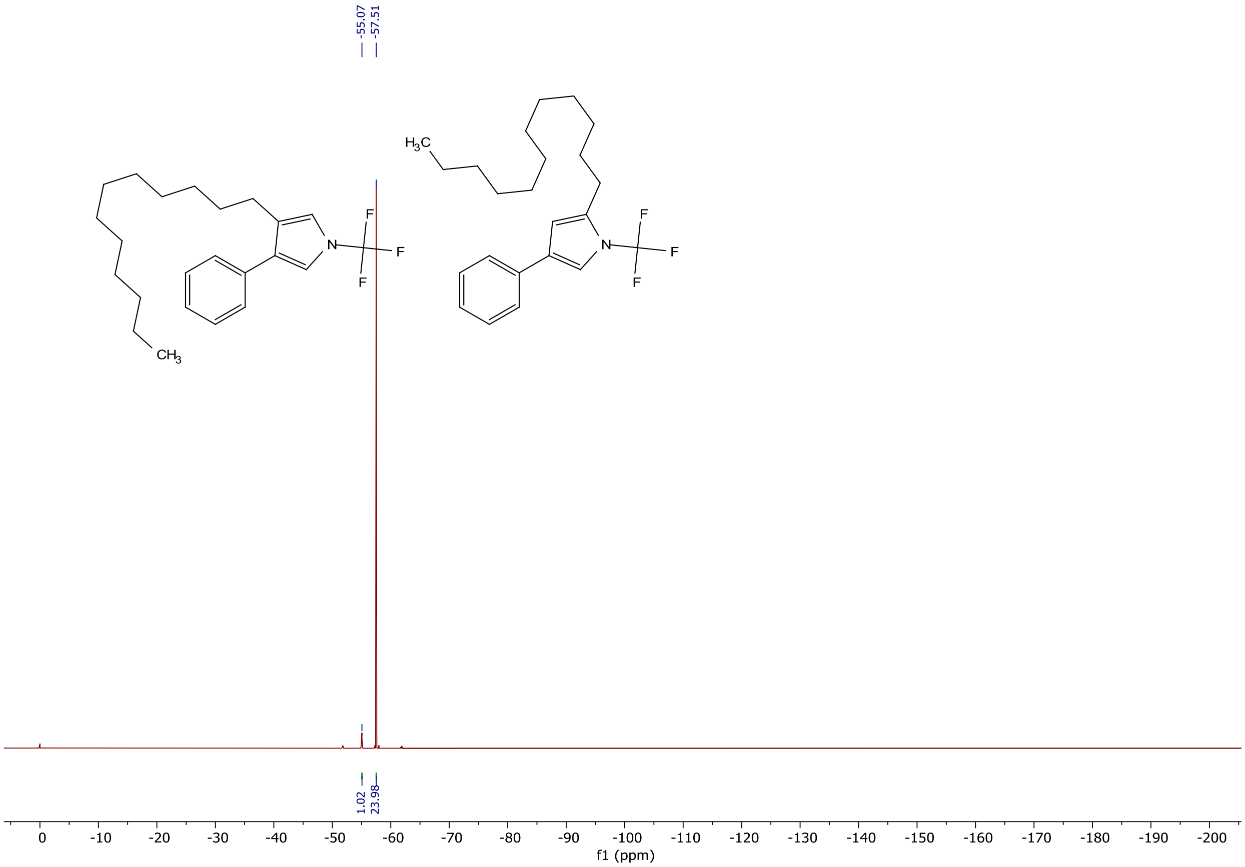

^{1}H NMR (401 MHz, $CDCl_3$) of **3g** + **3g'**

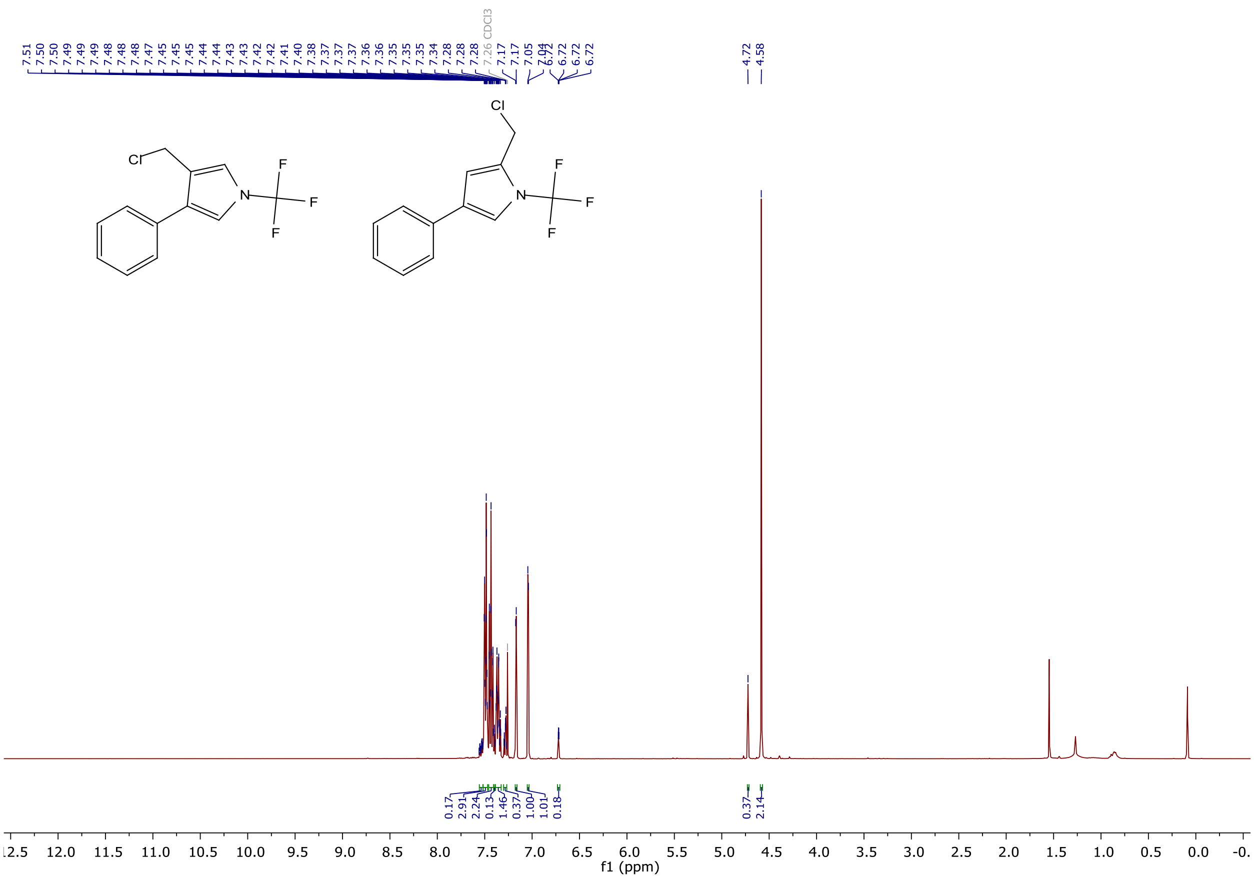

^{13}C NMR (101 MHz, $CDCl_3$) of **3g** + **3g'**

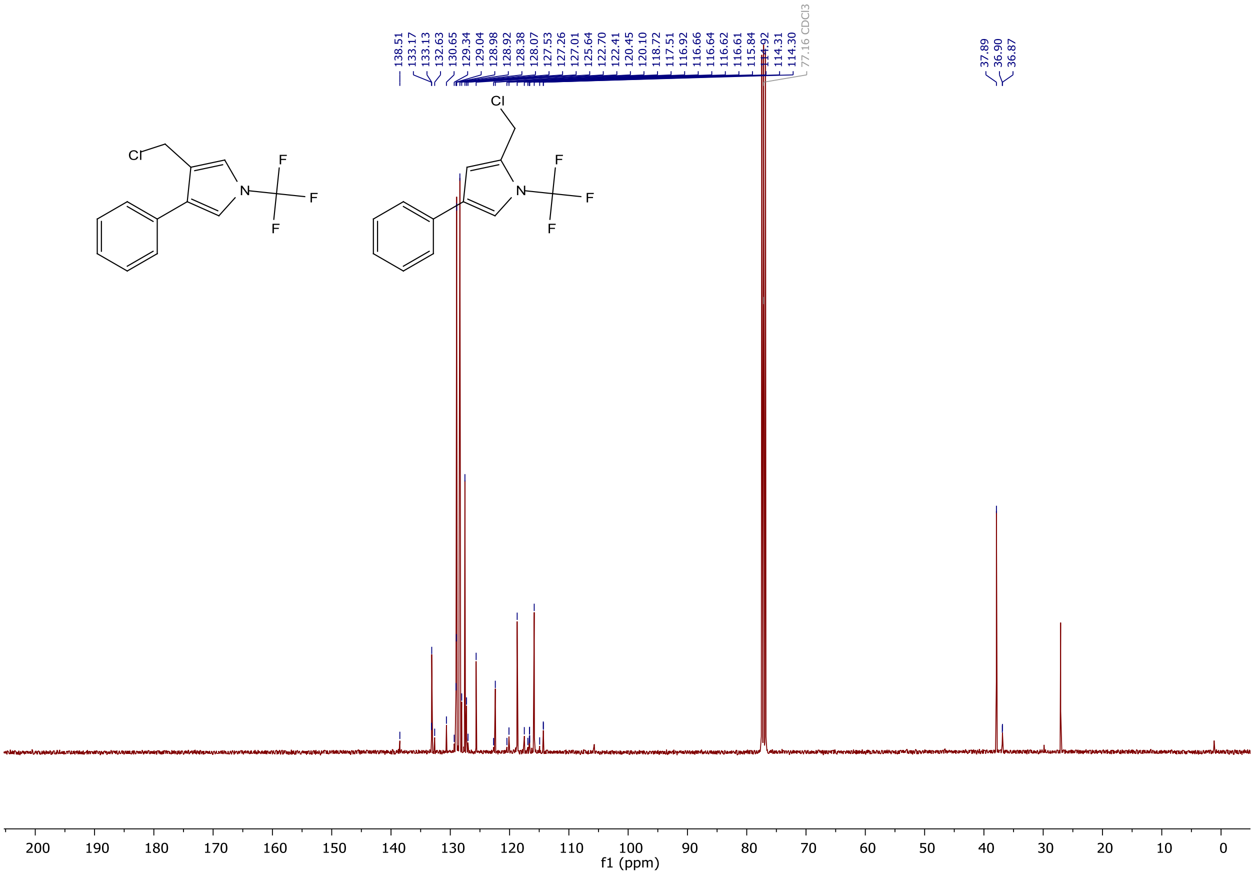

^{19}F NMR (377 MHz, $CDCl_3$) of **3g** + **3g’**

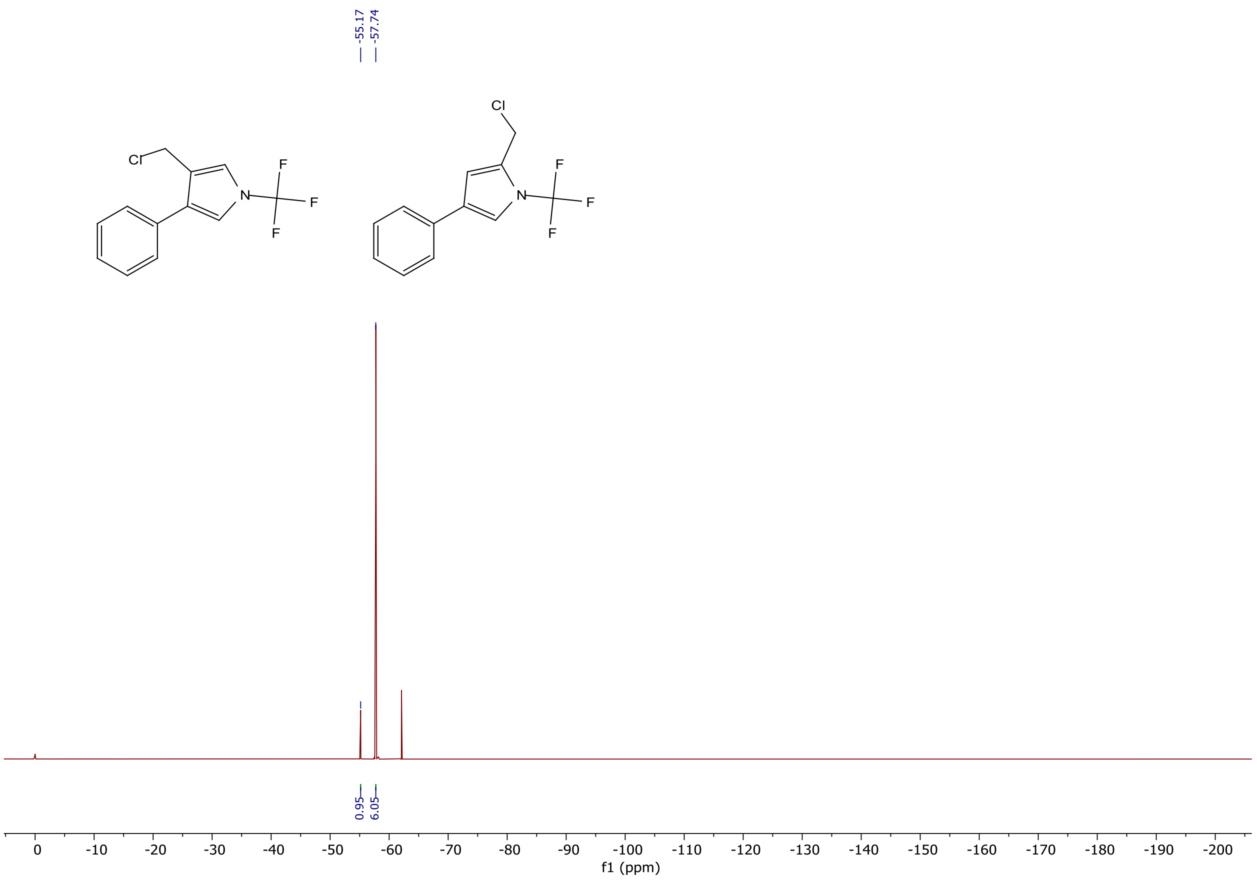

^{1}H NMR (401 MHz, $CDCl_3$) of **3h**

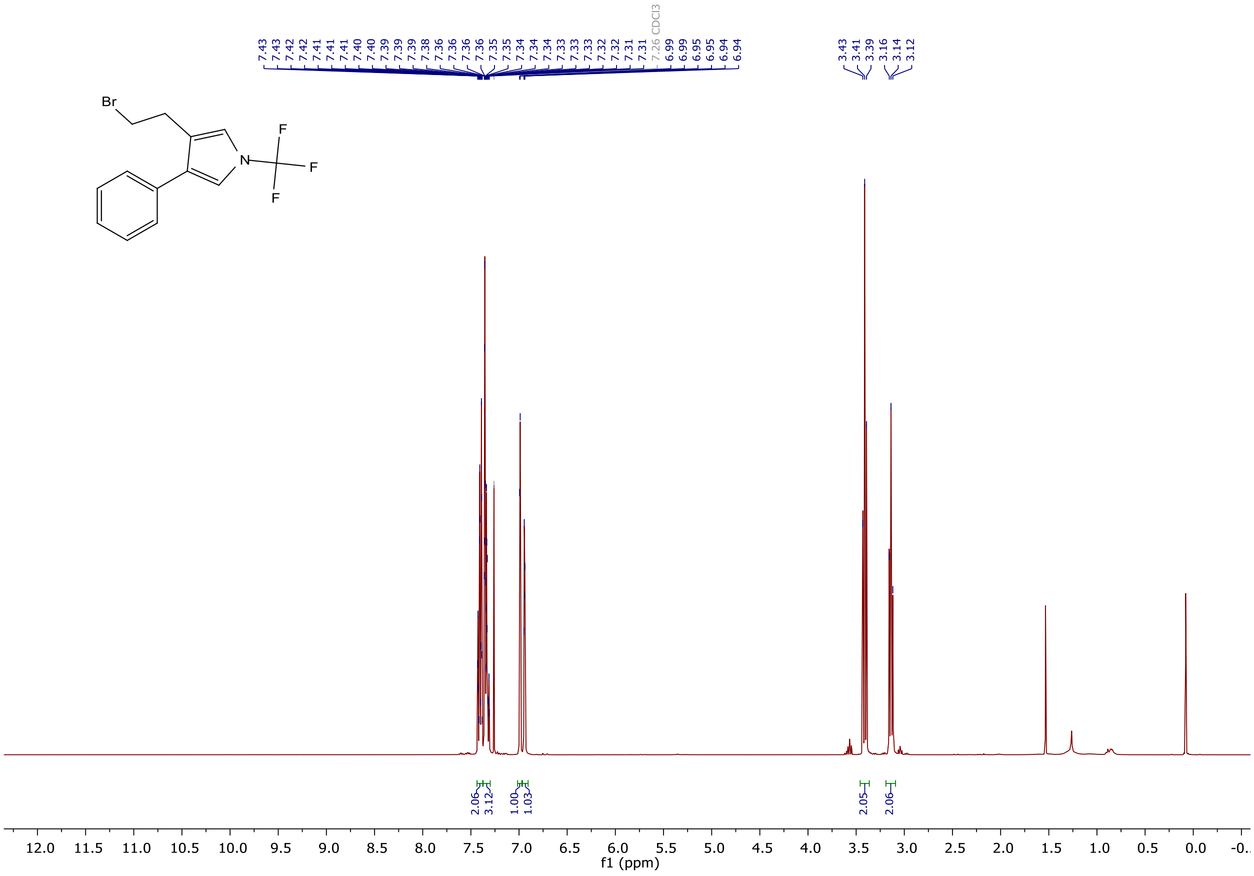

^{13}C NMR (101 MHz, $CDCl_3$) of **3h**

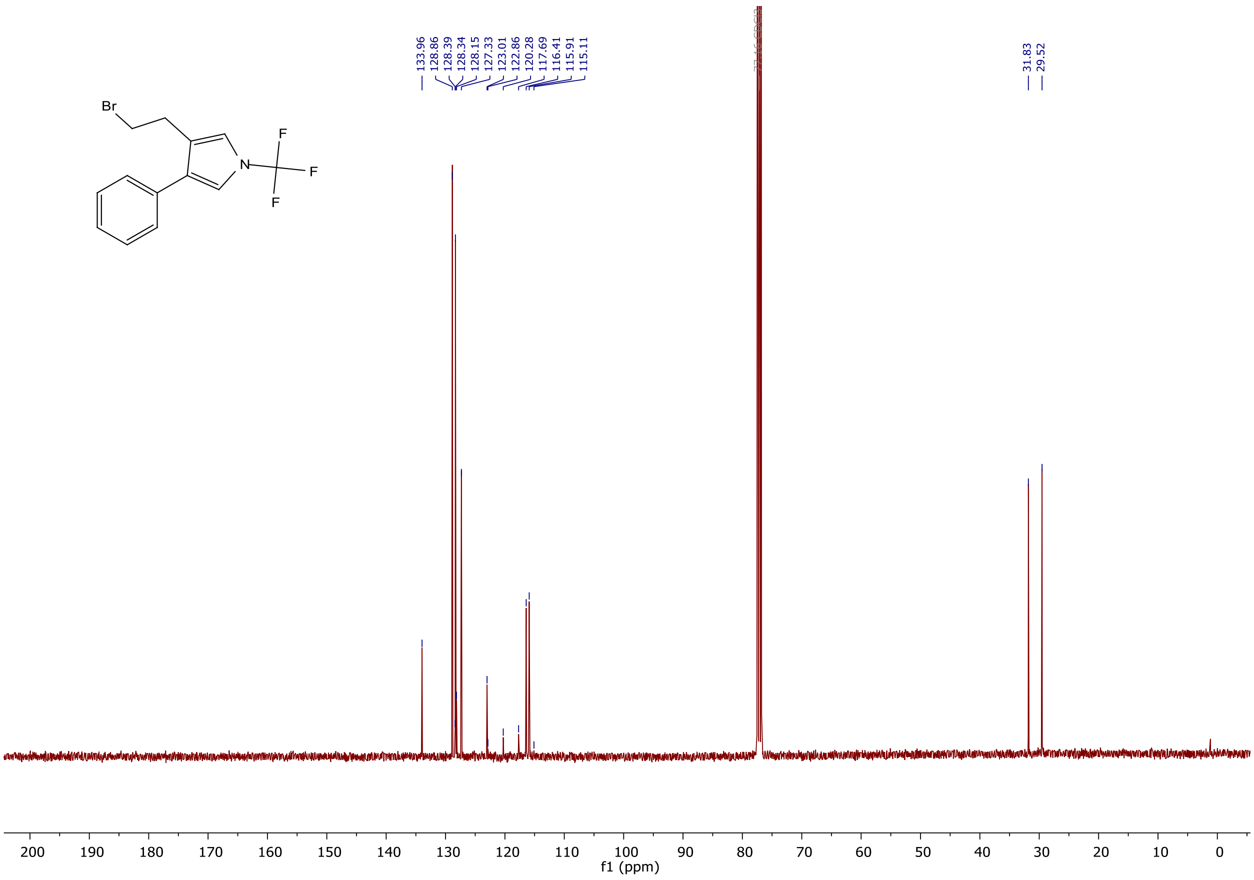

^{19}F NMR (377 MHz, $CDCl_3$) of **3h**

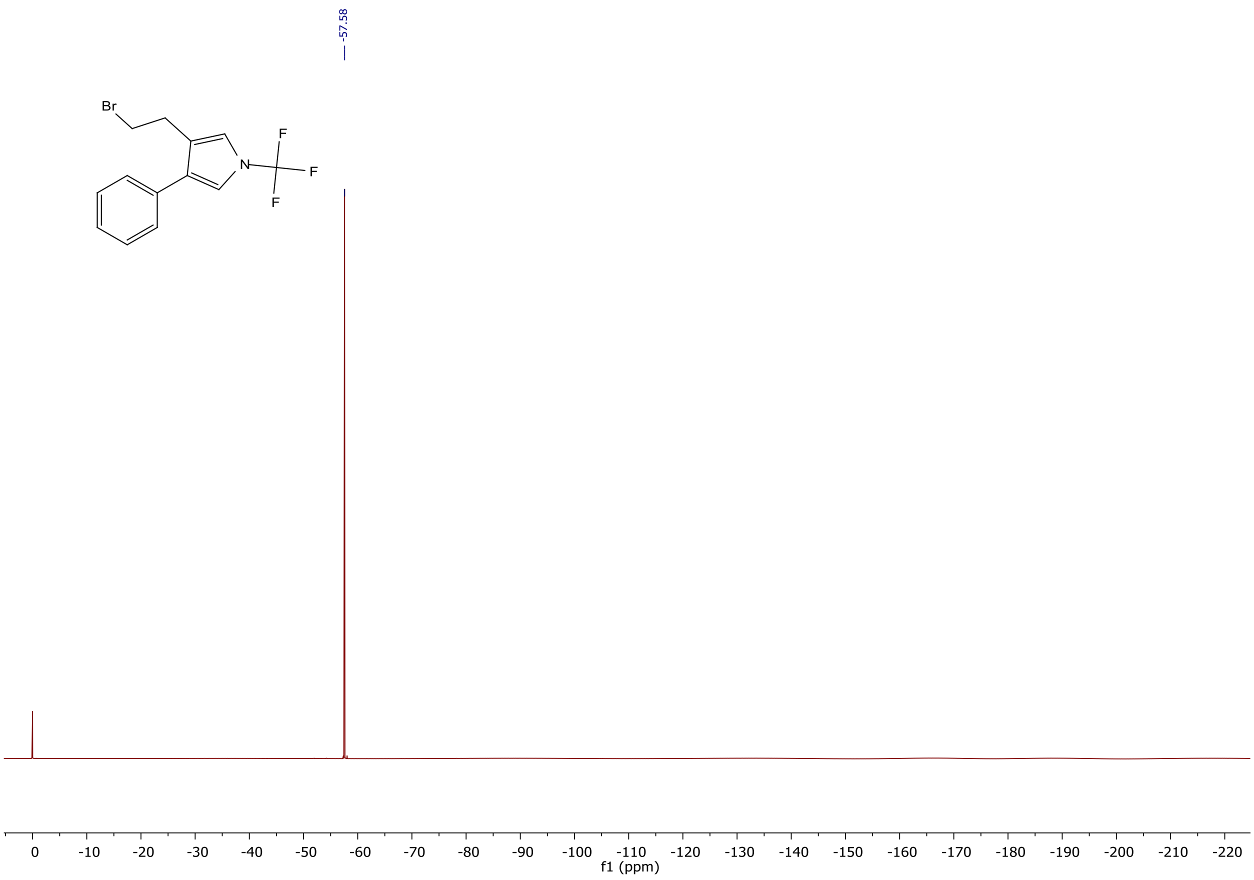

^1H NMR (401 MHz, $CDCl_3$) of **3i** + **3i'**

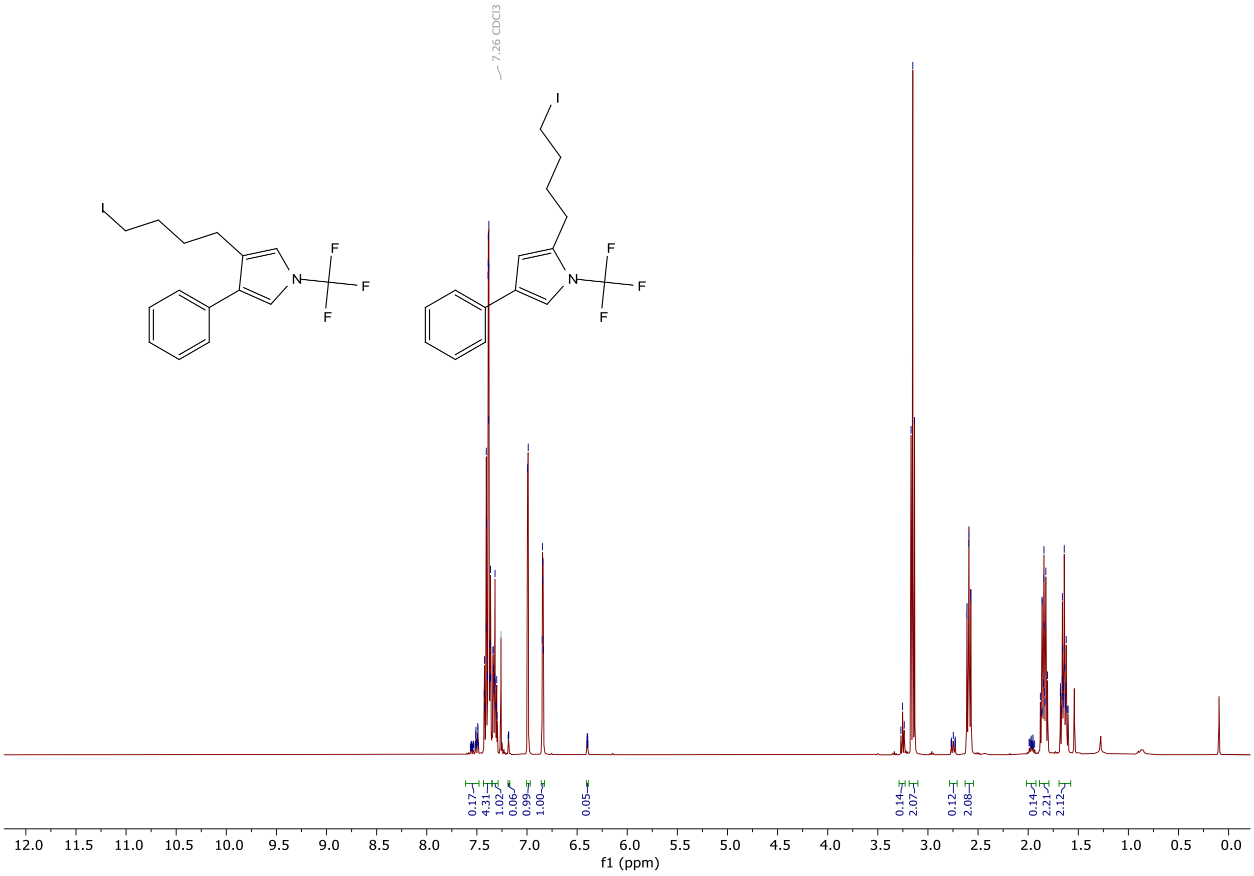

^{13}C NMR (101 MHz, $CDCl_3$) of **3i** + **3i'**

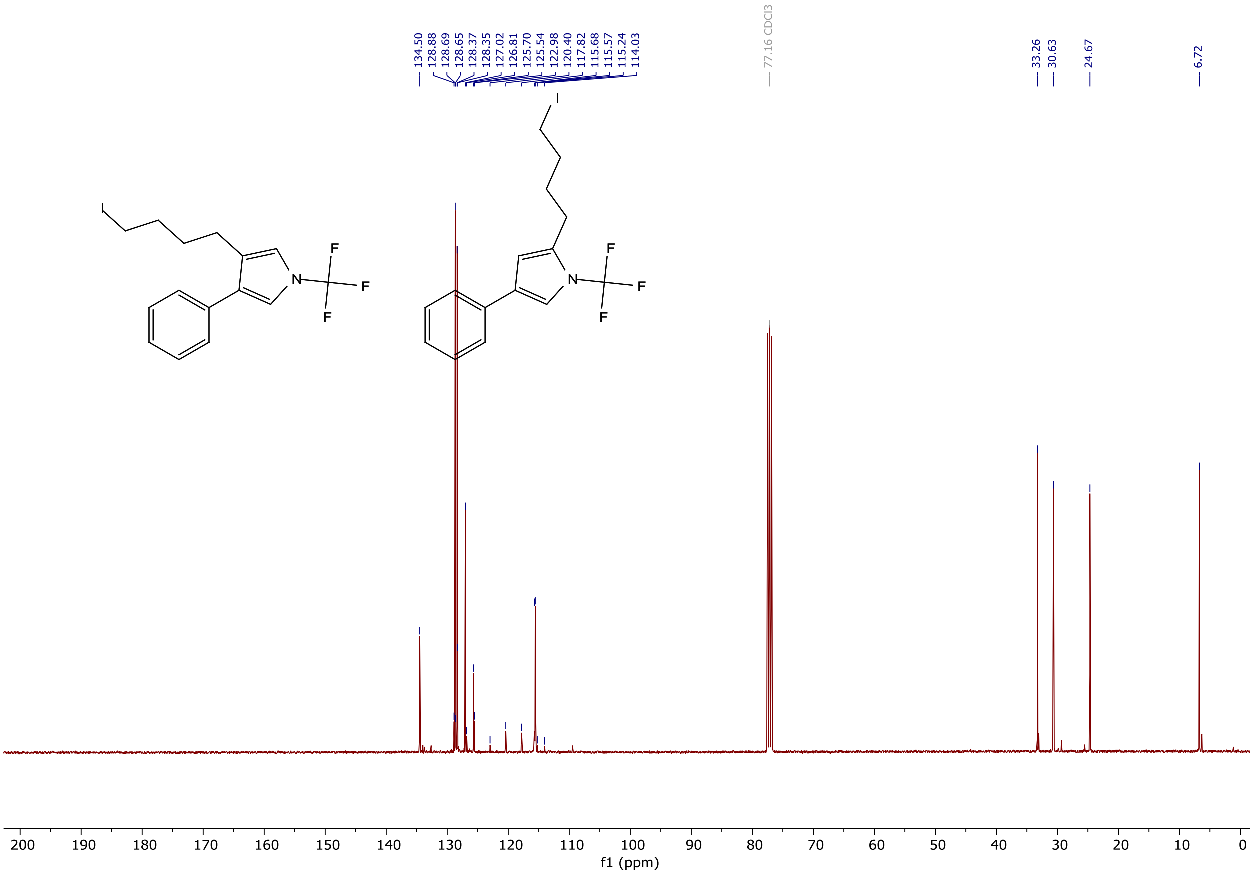

^{19}F NMR (377 MHz, $CDCl_3$) of **3i** + **3i'**

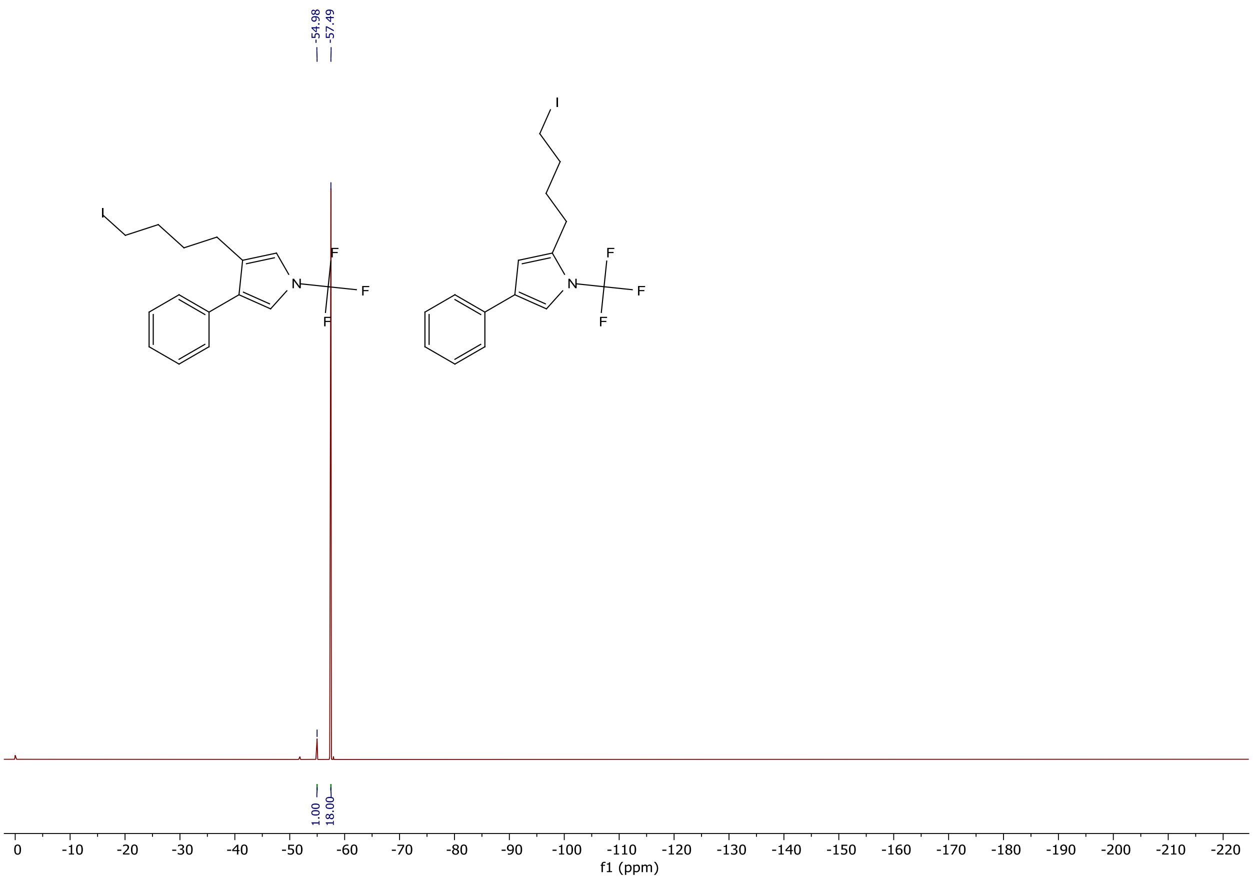

^{1}H NMR (401 MHz, $CDCl_3$) of **3j** + **3j’**

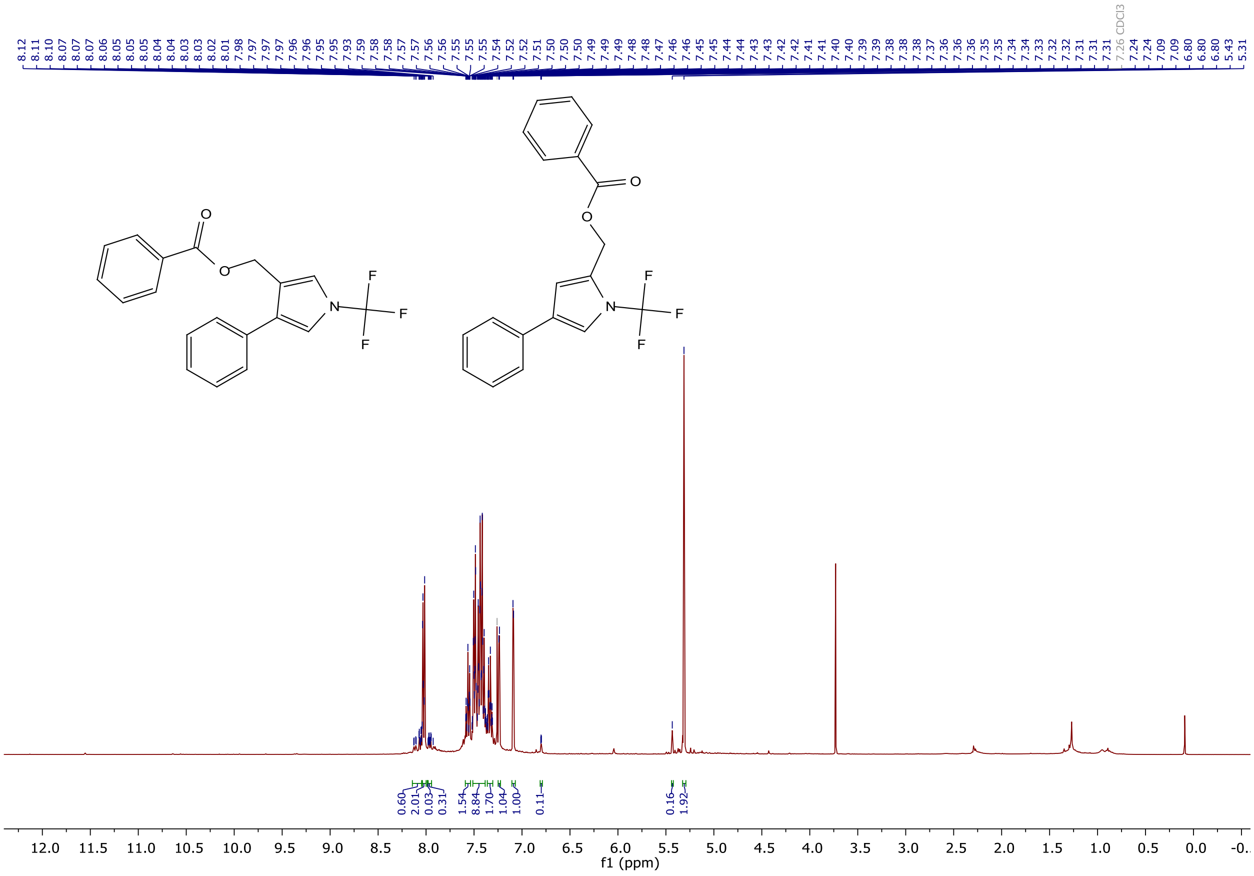

^{13}C NMR (101 MHz, $CDCl_3$) of **3j** + **3j'**

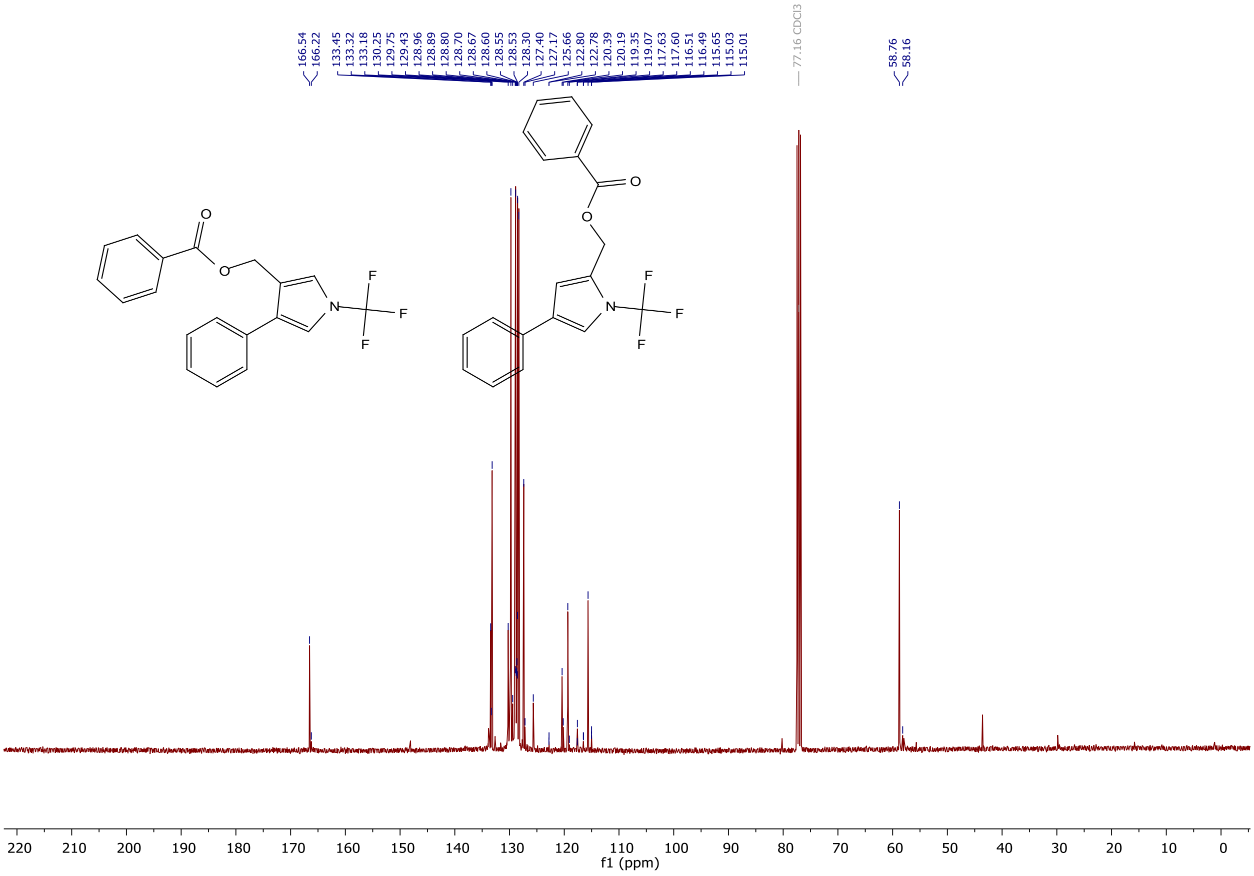

^{19}F NMR (377 MHz, $CDCl_3$) of **3j** + **3j'**

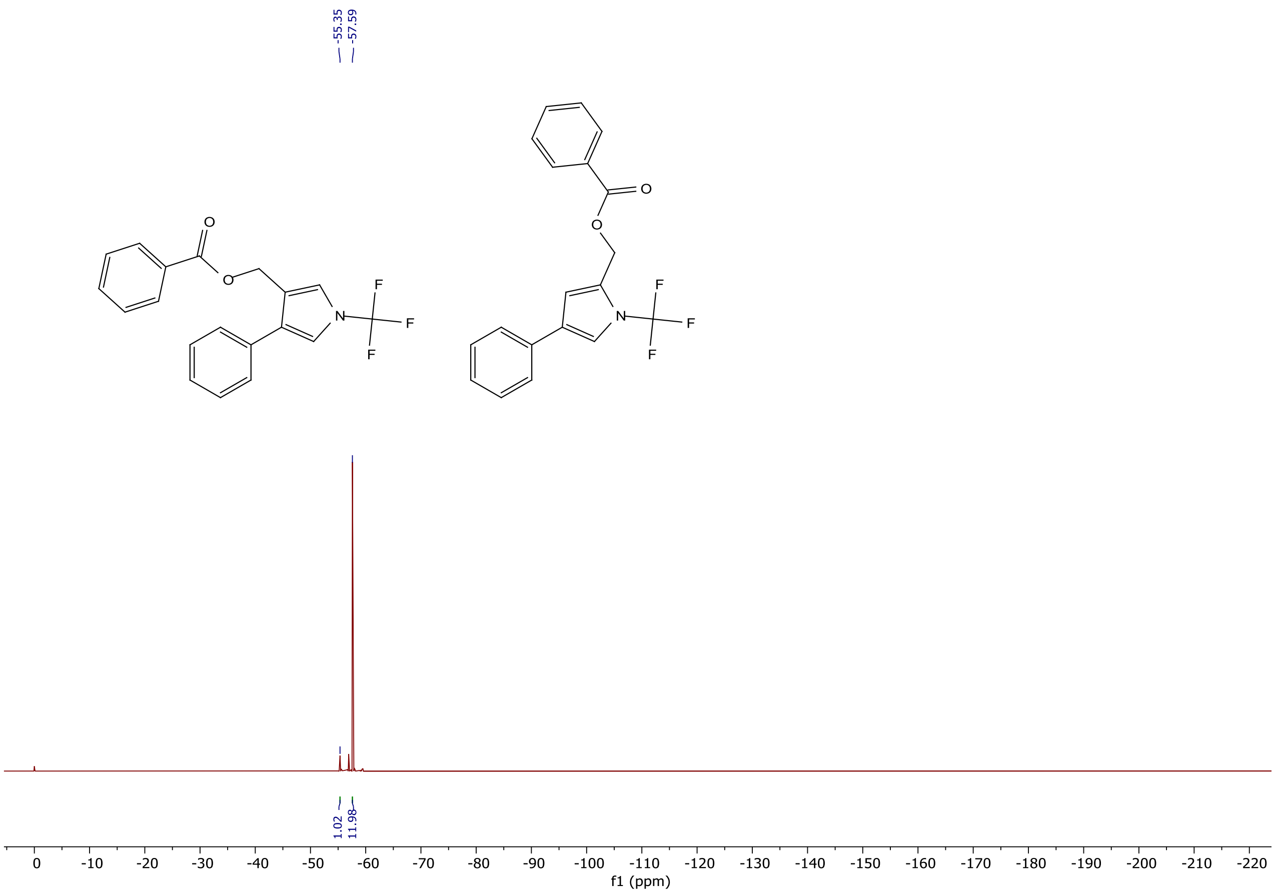

^{1}H NMR (401 MHz, $CDCl_3$) of **3k** + **3k’**

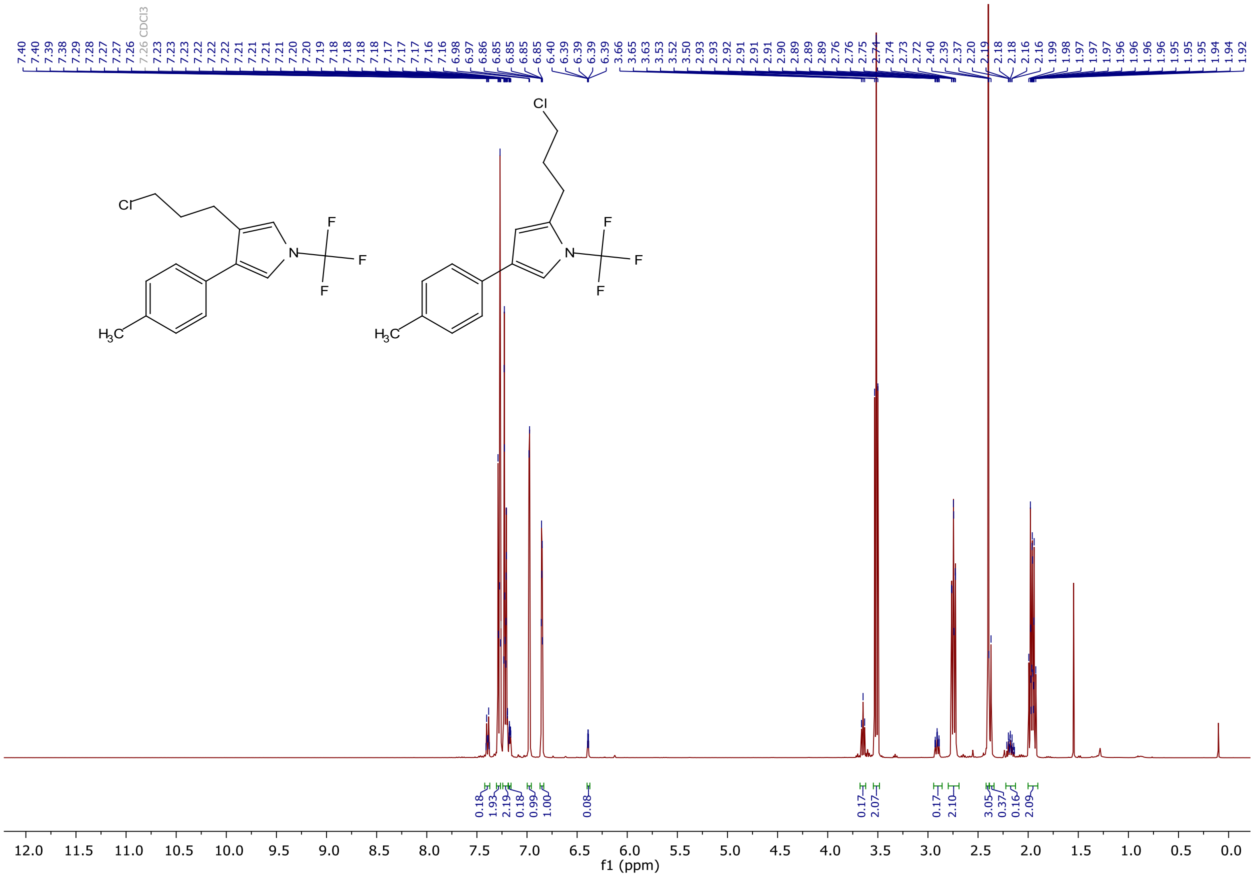

^{13}C NMR (101 MHz, CDCl$_3$) of **3k** + **3k’**

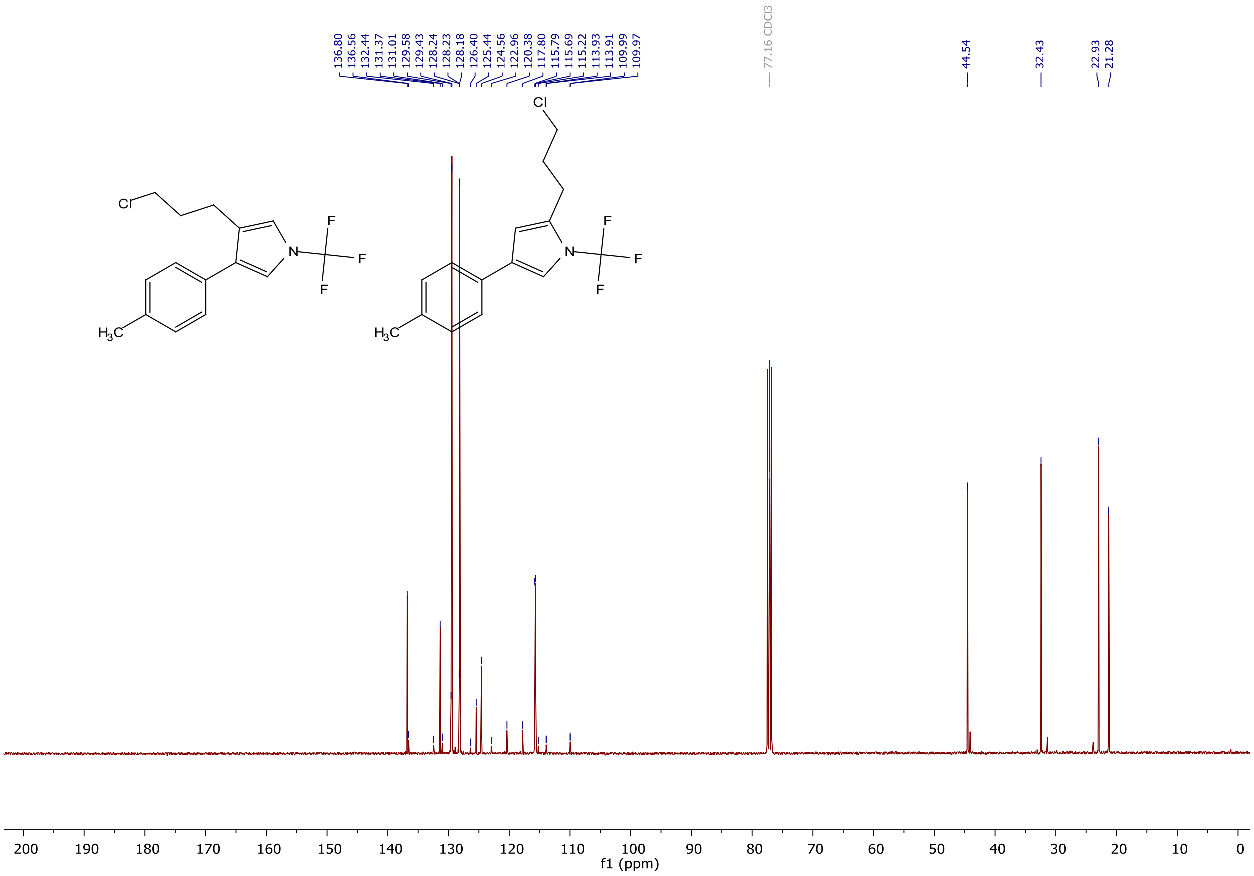

^{19}F NMR (377 MHz, $CDCl_3$) of **3k** + **3k’**

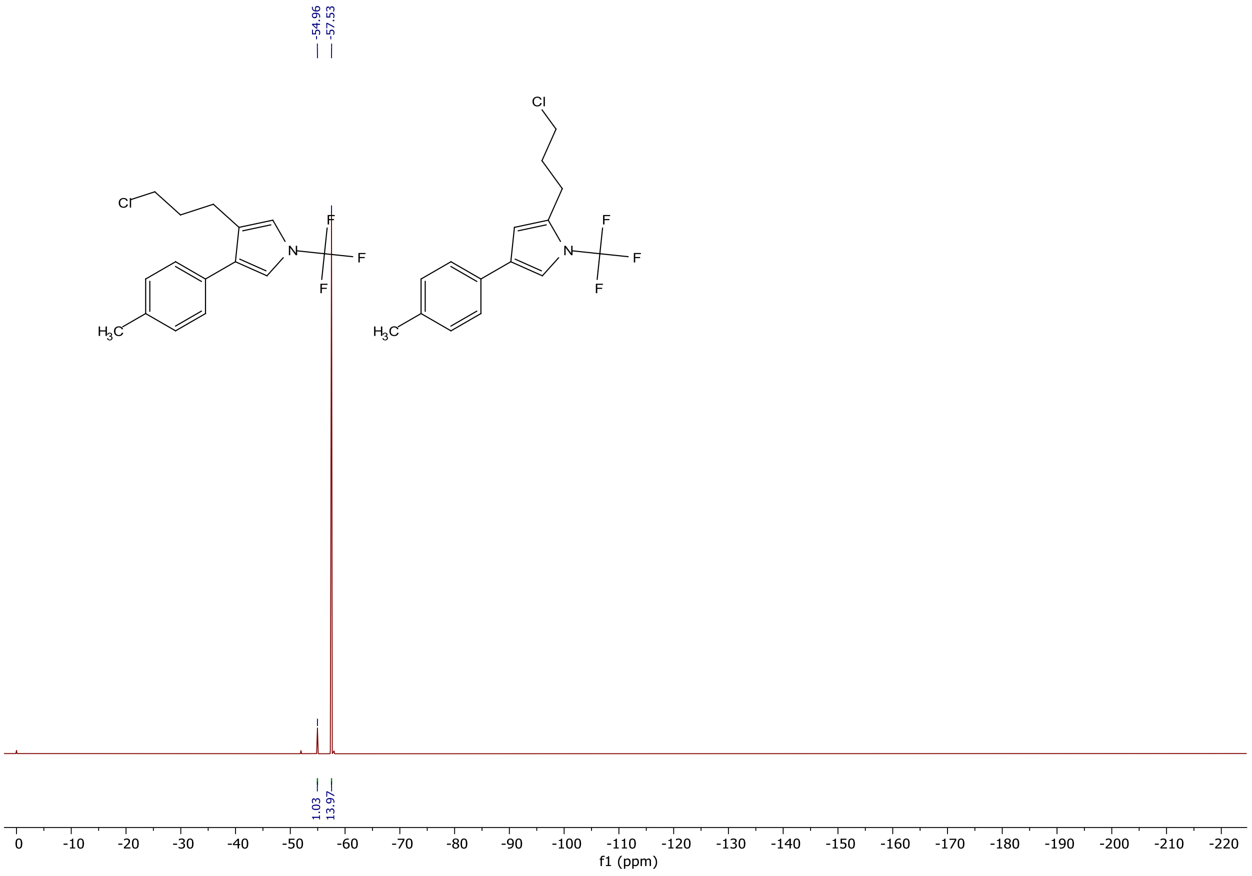

^{1}H NMR (401 MHz, $CDCl_3$) of **3l**

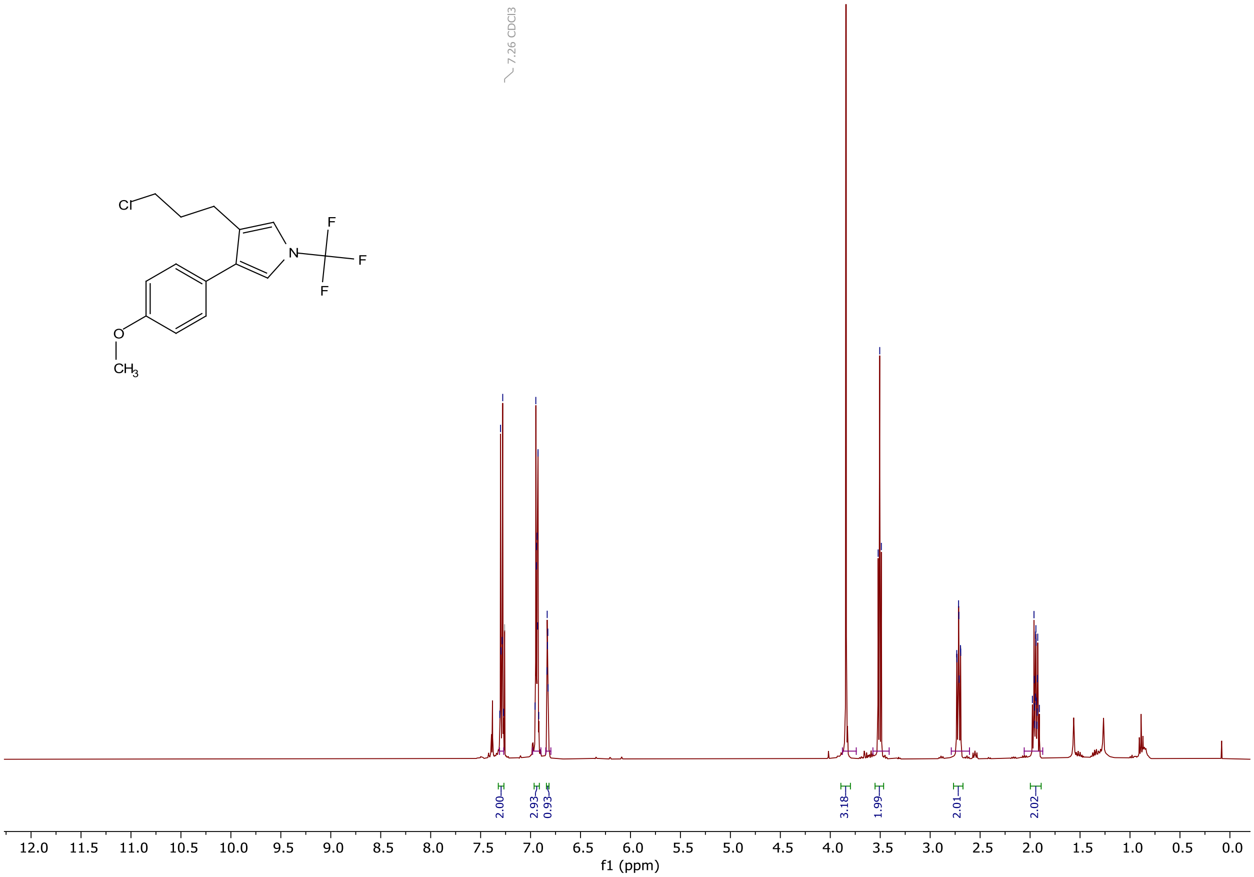

^{13}C NMR (101 MHz, $CDCl_3$) of **3l**

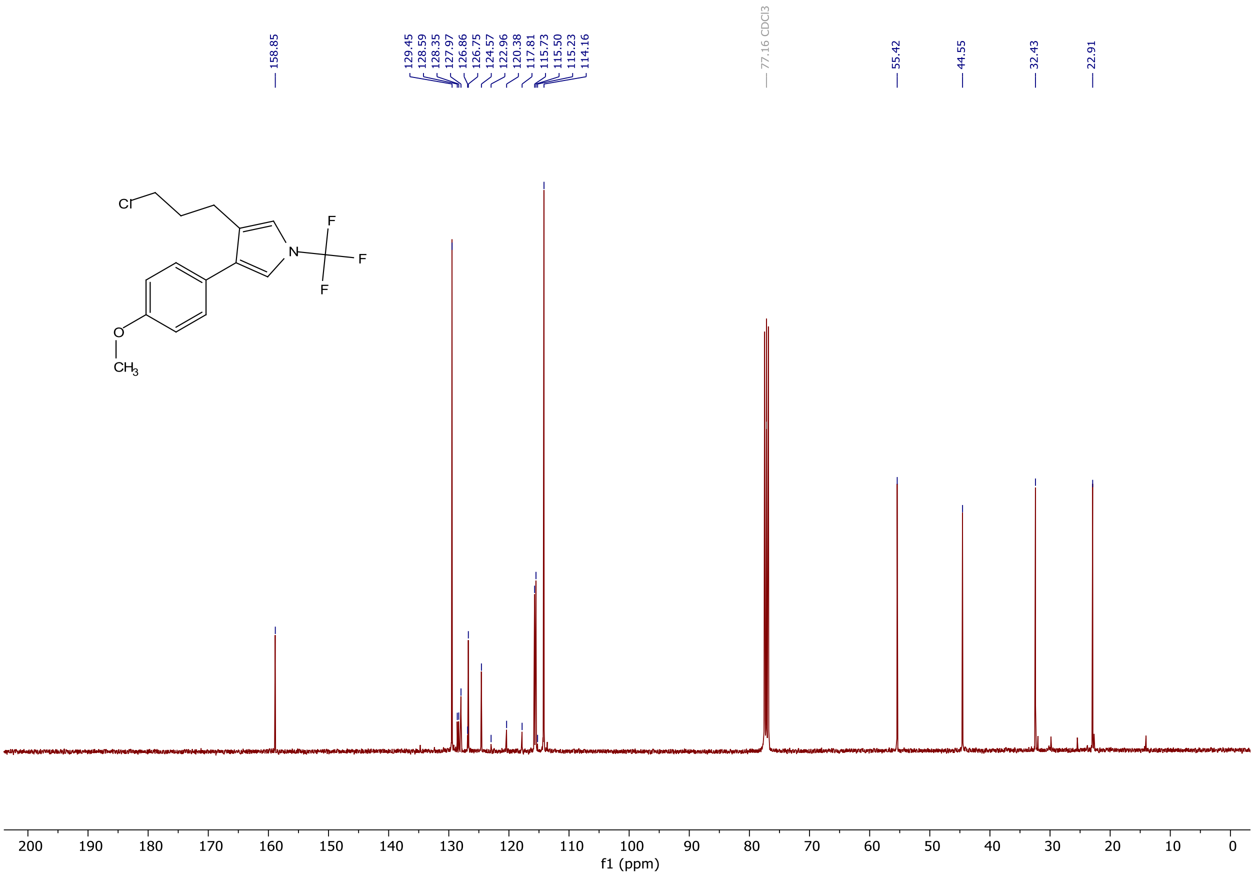

^{19}F NMR (377 MHz, $CDCl_3$) of **3l**

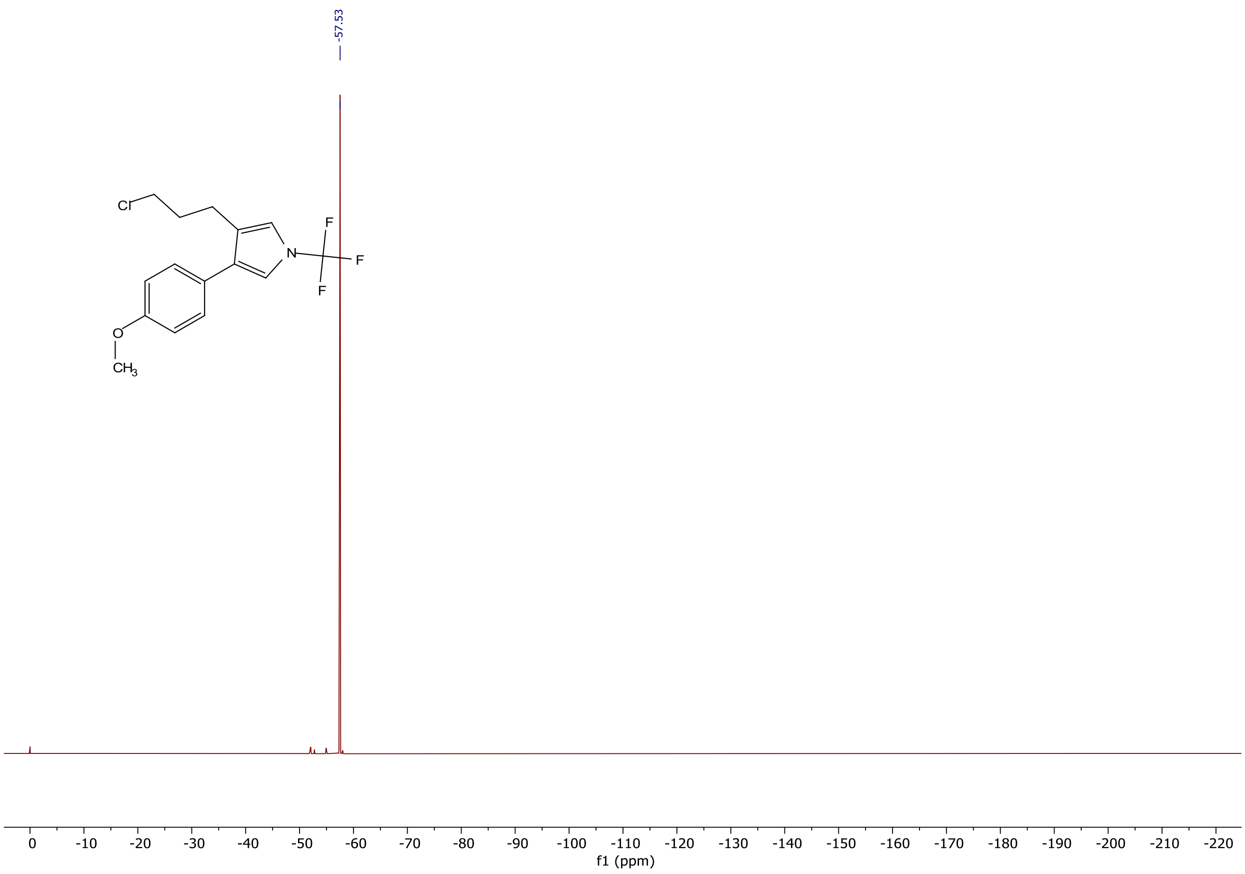

^{1}H NMR (401 MHz, $CDCl_3$) of **3m** + **3m’**

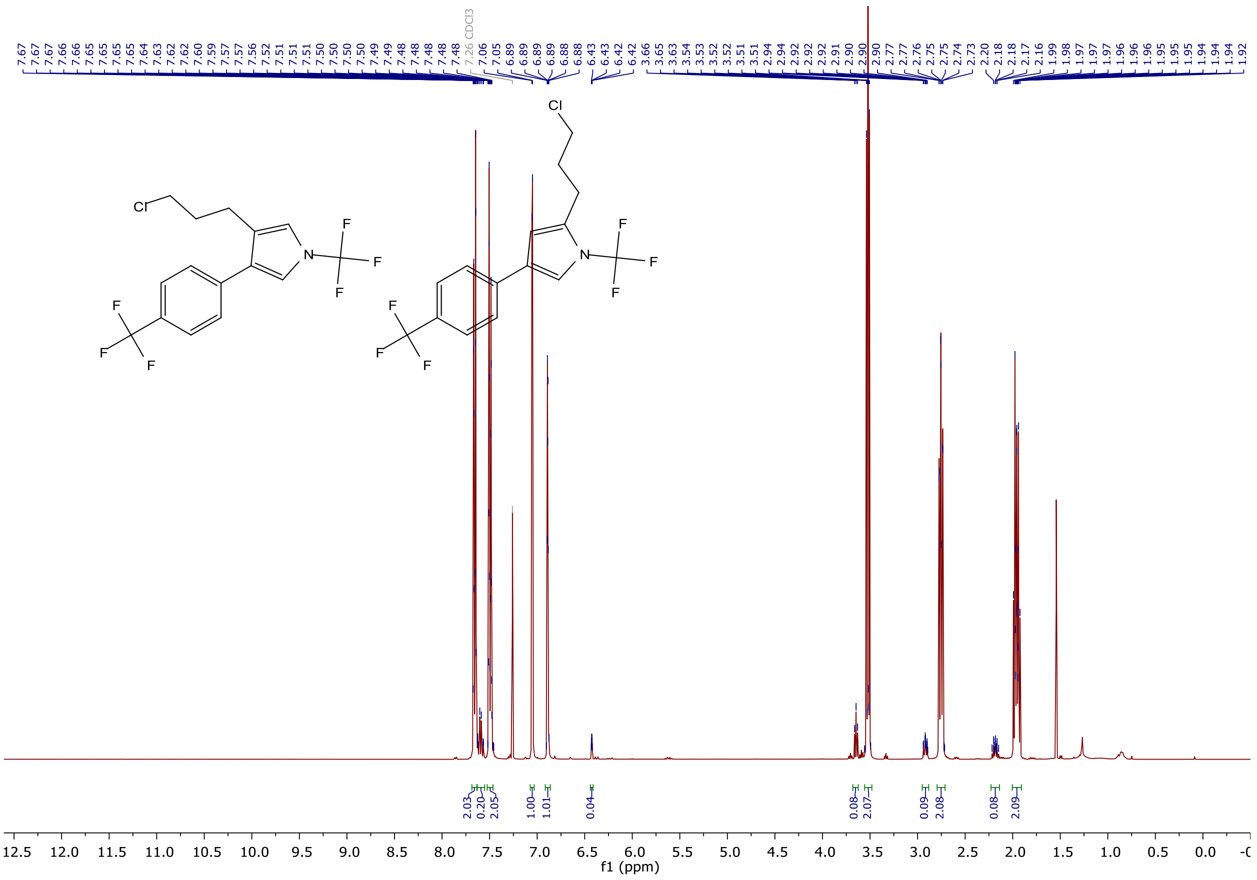

^{13}C NMR (101 MHz, $CDCl_3$) of **3m** + **3m'**

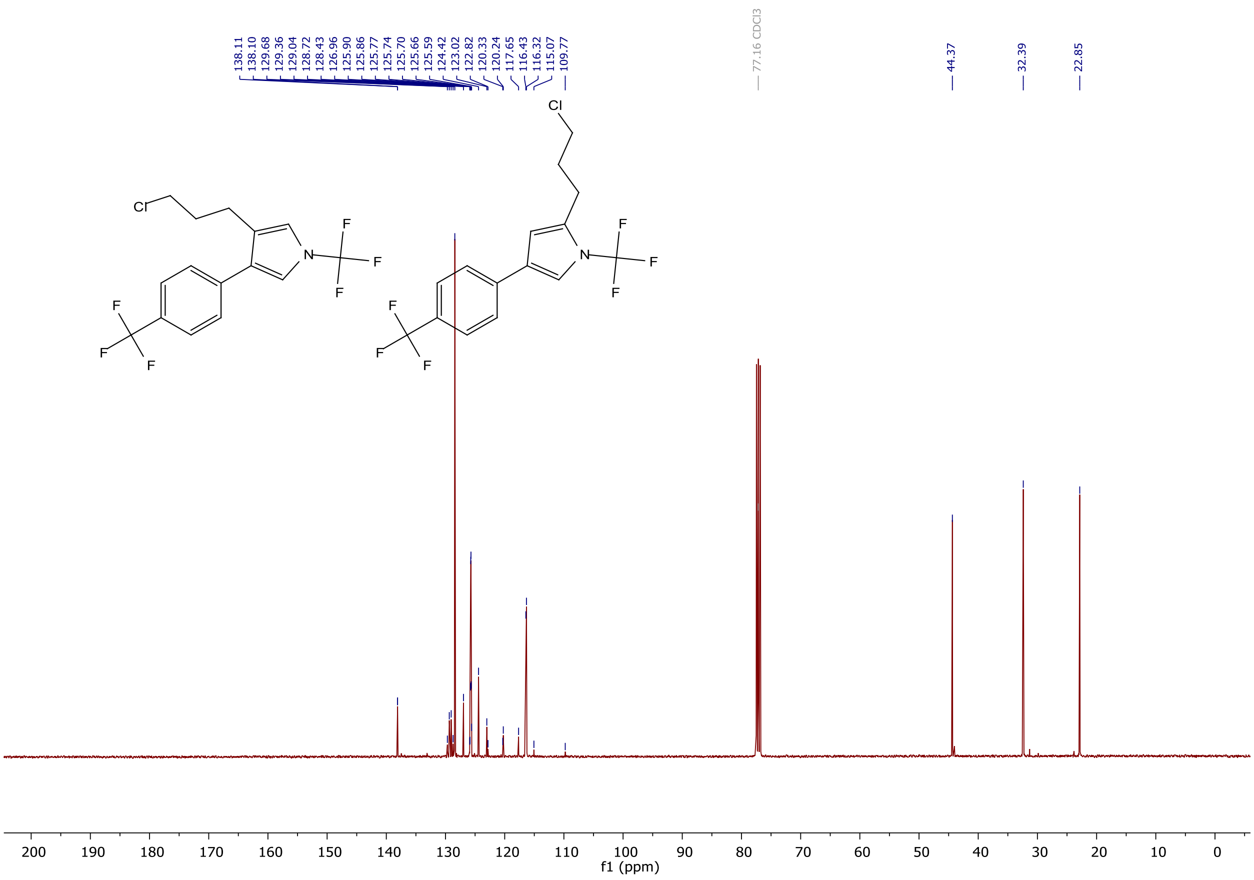

^{19}F NMR (377 MHz, $CDCl_3$) of **3m** + **3m'**

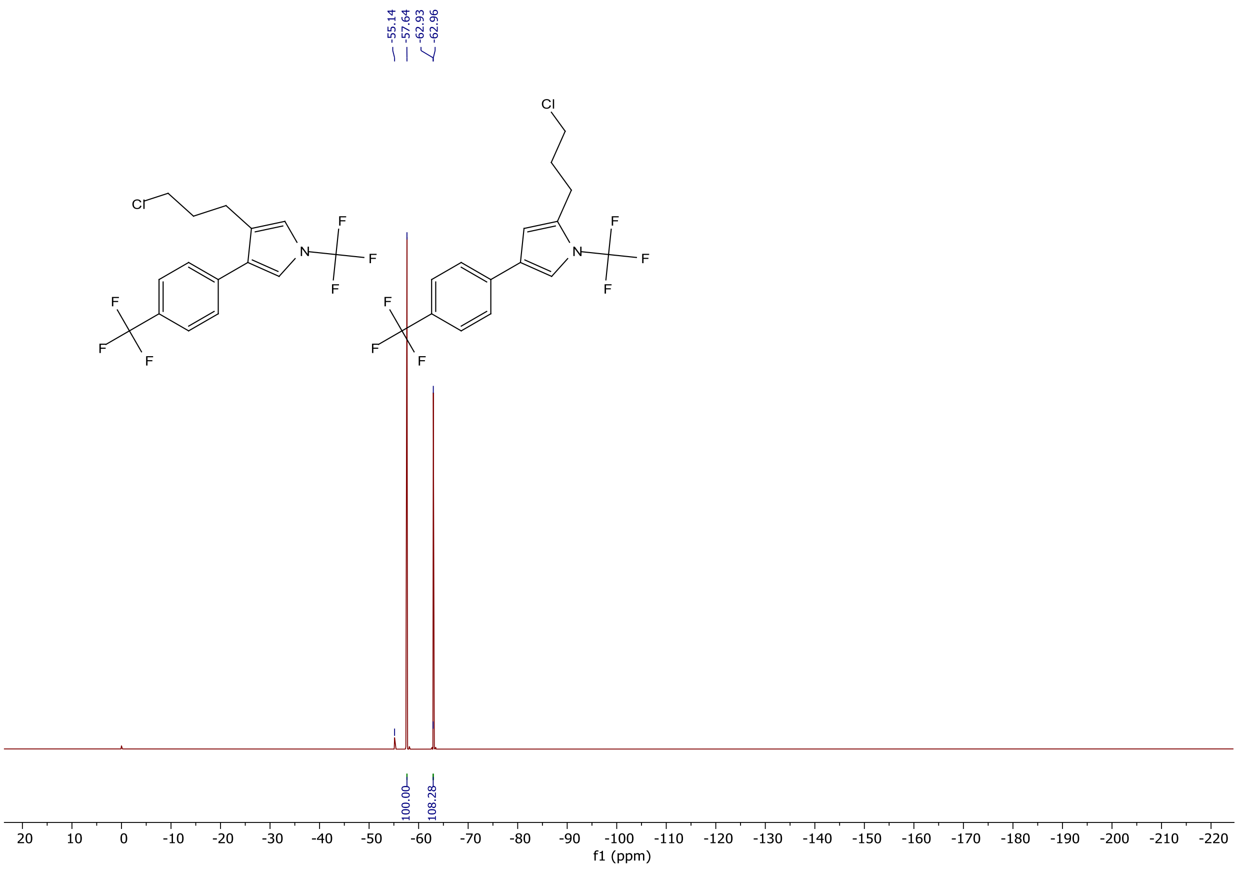

^{1}H NMR (401 MHz, $CDCl_3$) of **3m**

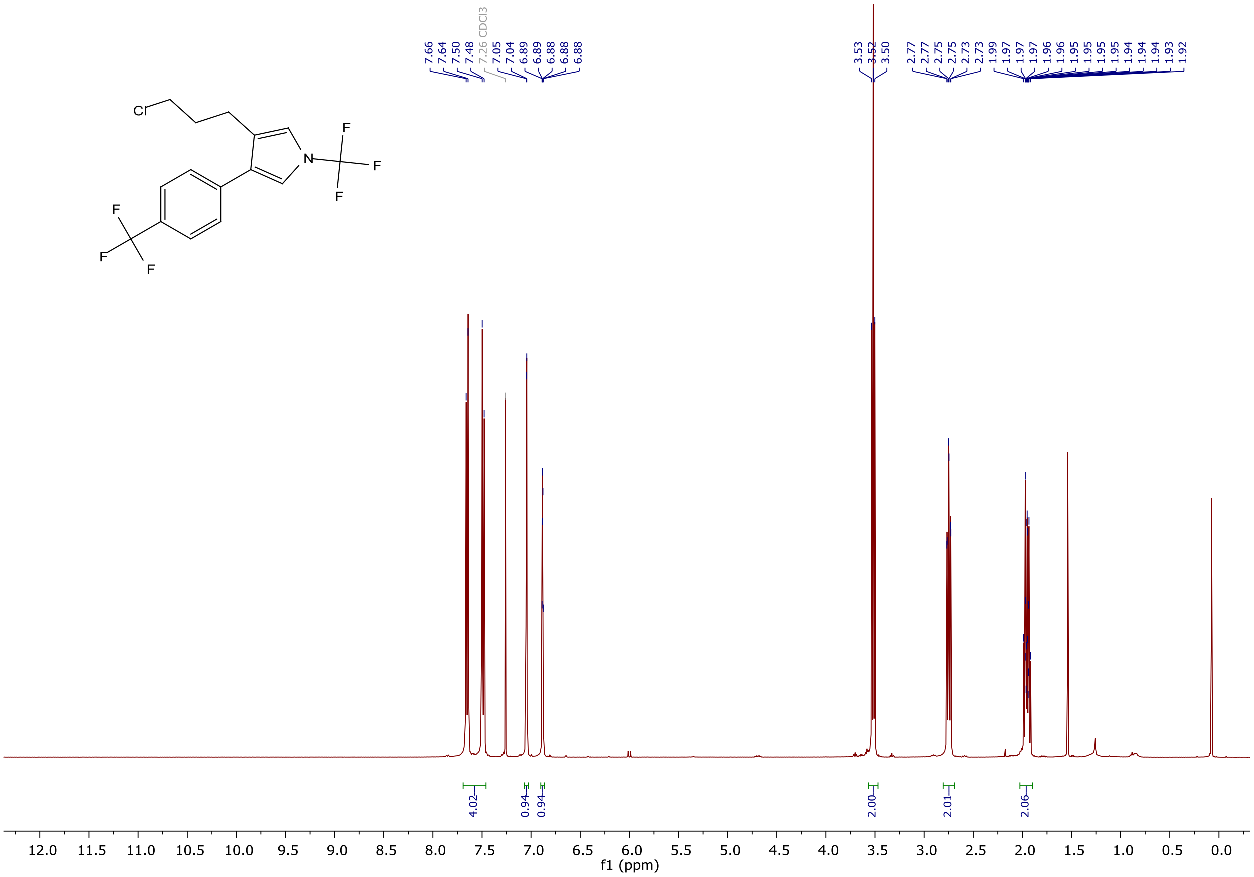

^{13}C NMR (101 MHz, $CDCl_3$) of **3m**

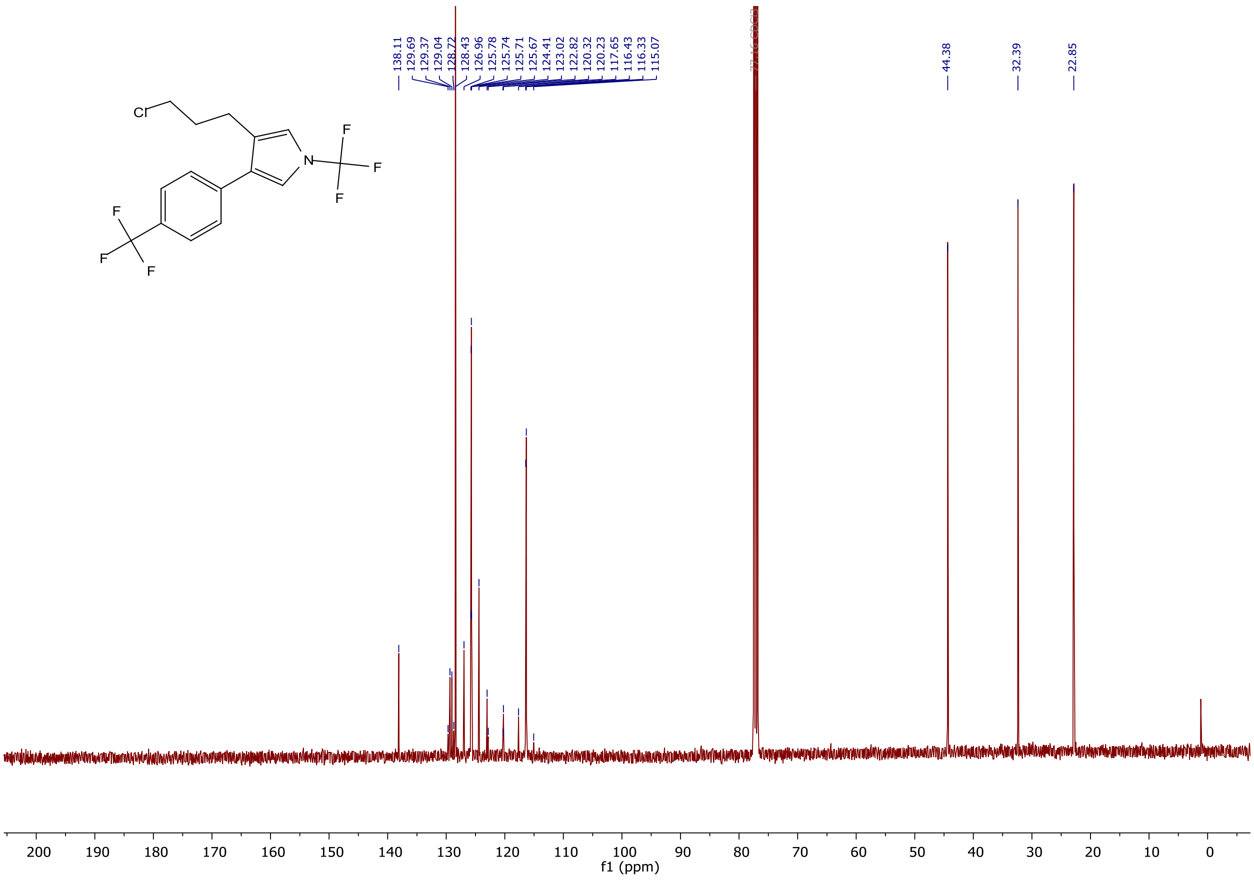

^{19}F NMR (377 MHz, $CDCl_3$) of **3m**

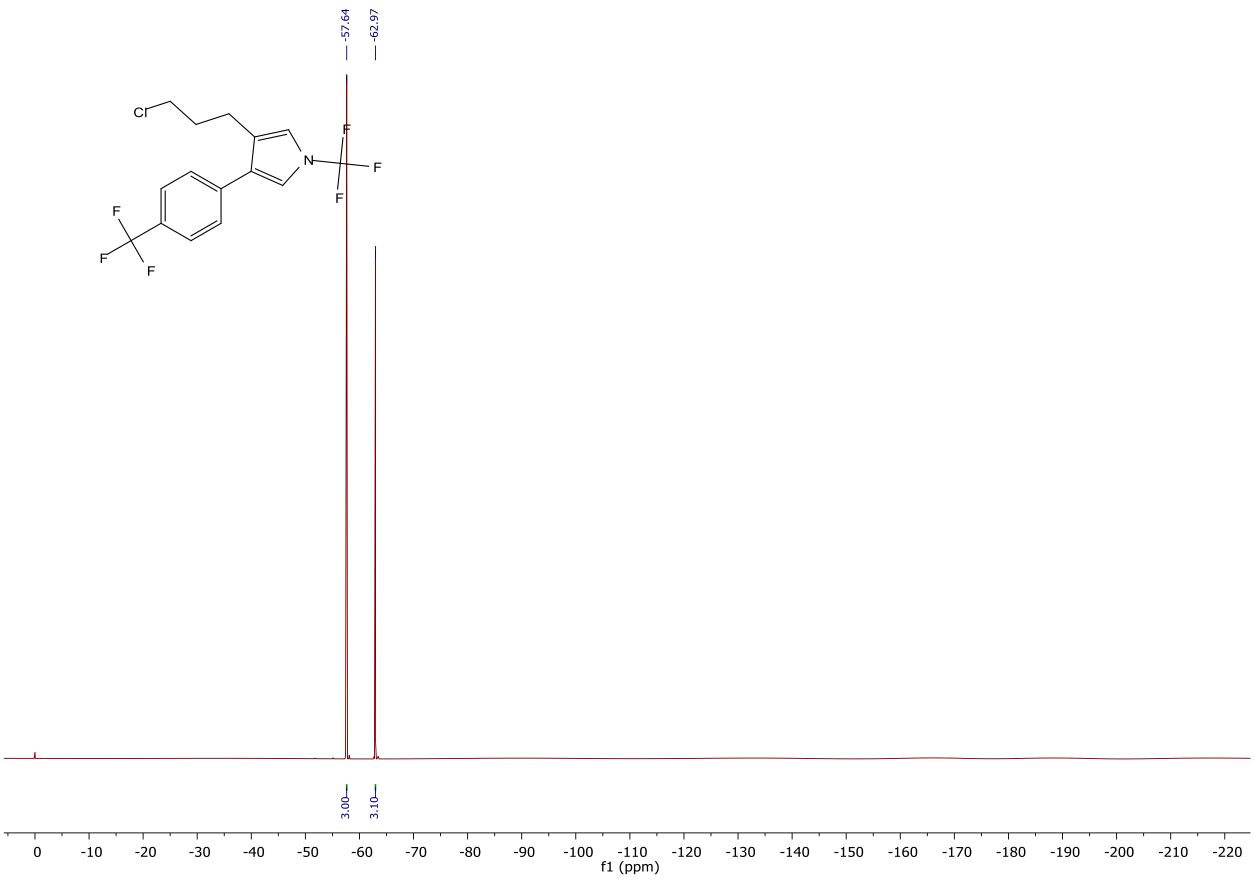

^{1}H NMR (400 MHz, $CDCl_3$) of **3n** + **3n’**

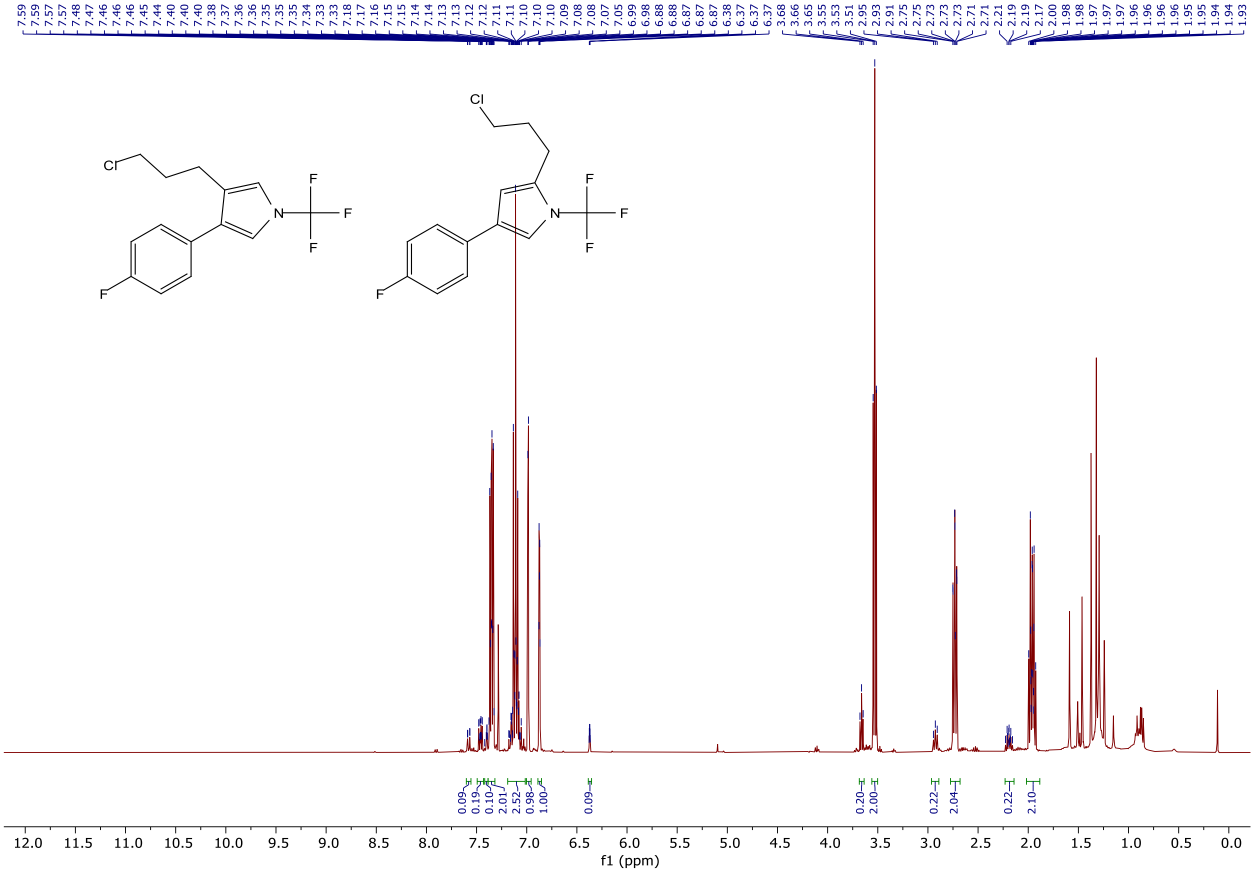

^{13}C NMR (101 MHz, $CDCl_3$) of **3n** + **3n'**

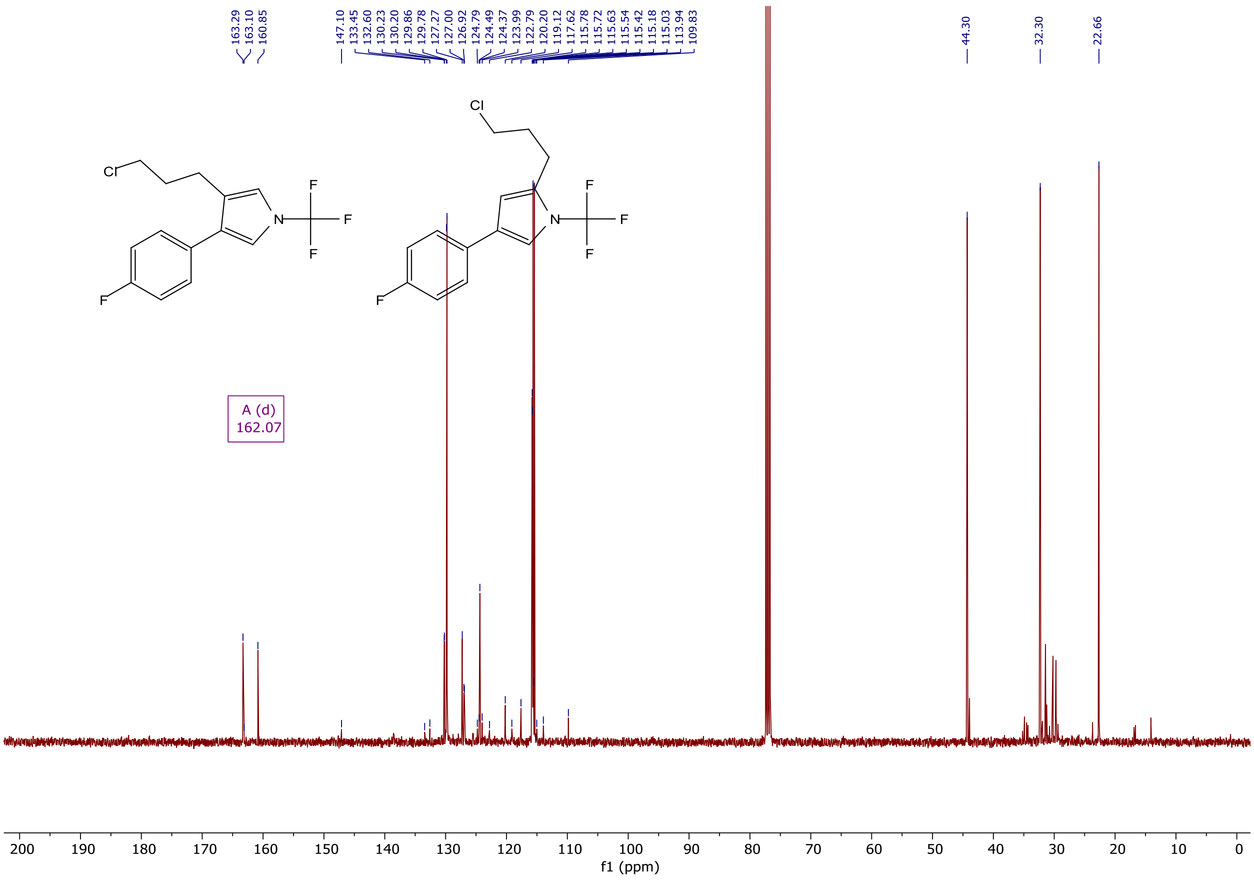

^{19}F NMR (376 MHz, $CDCl_3$) of **3n** + **3n’**

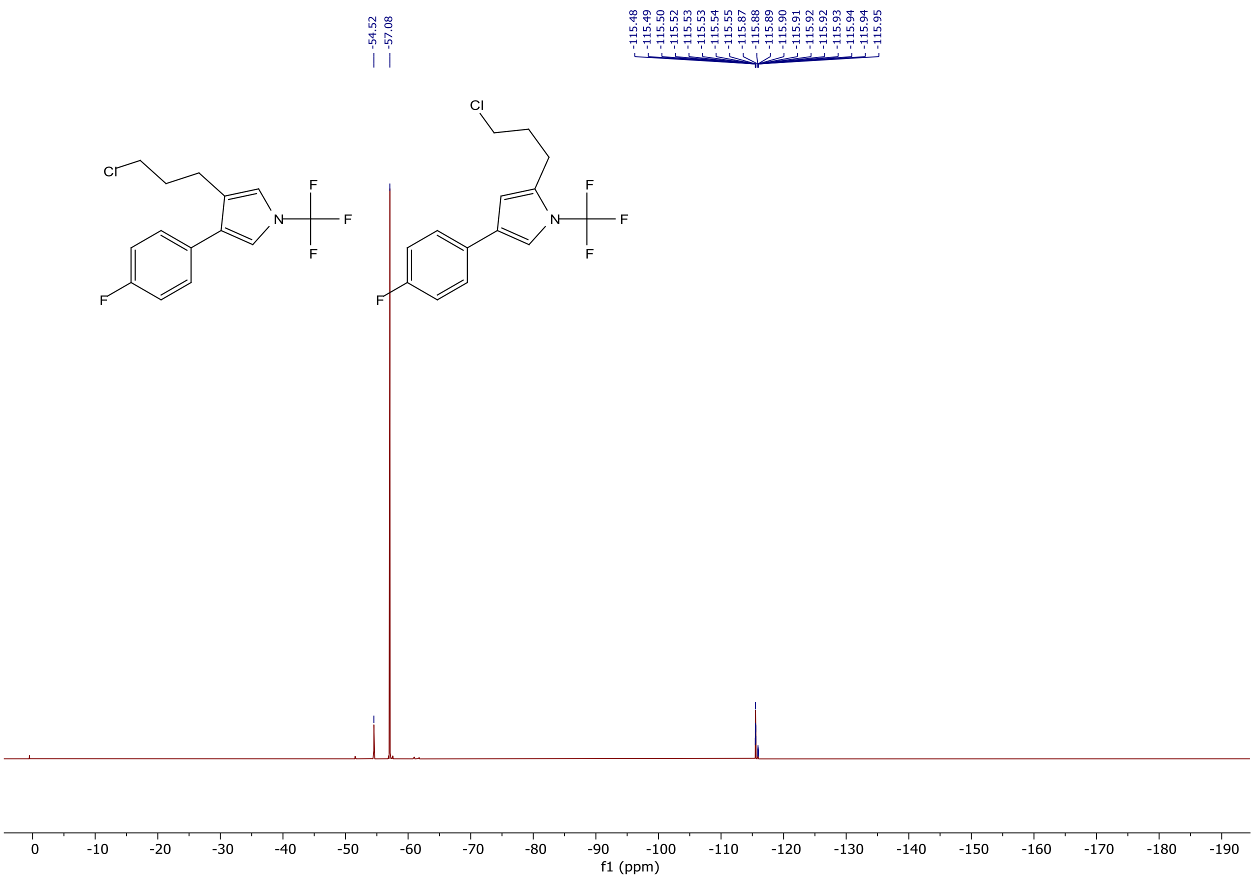

^1H NMR (401 MHz, $CDCl_3$) of **3n**

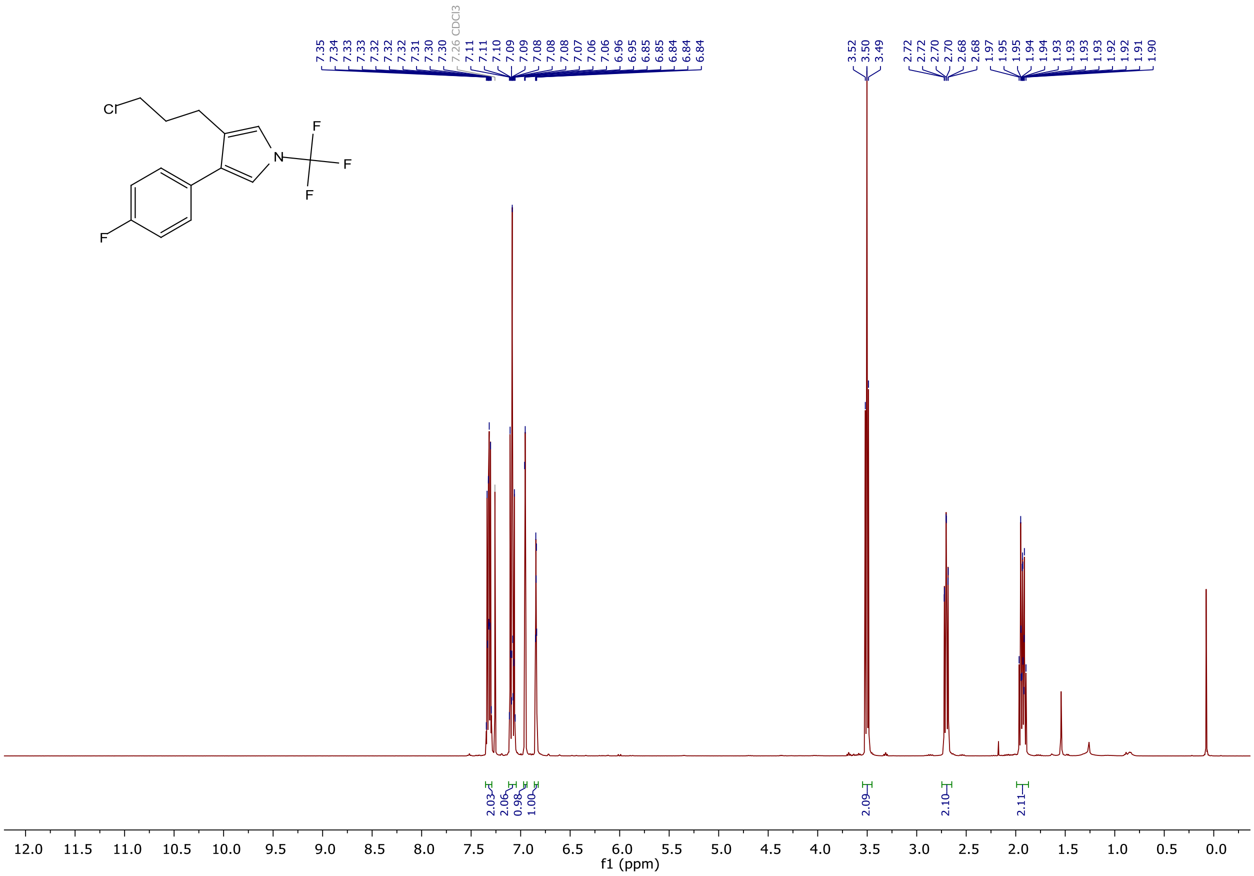

^{13}C NMR (101 MHz, $CDCl_3$) of **3n**

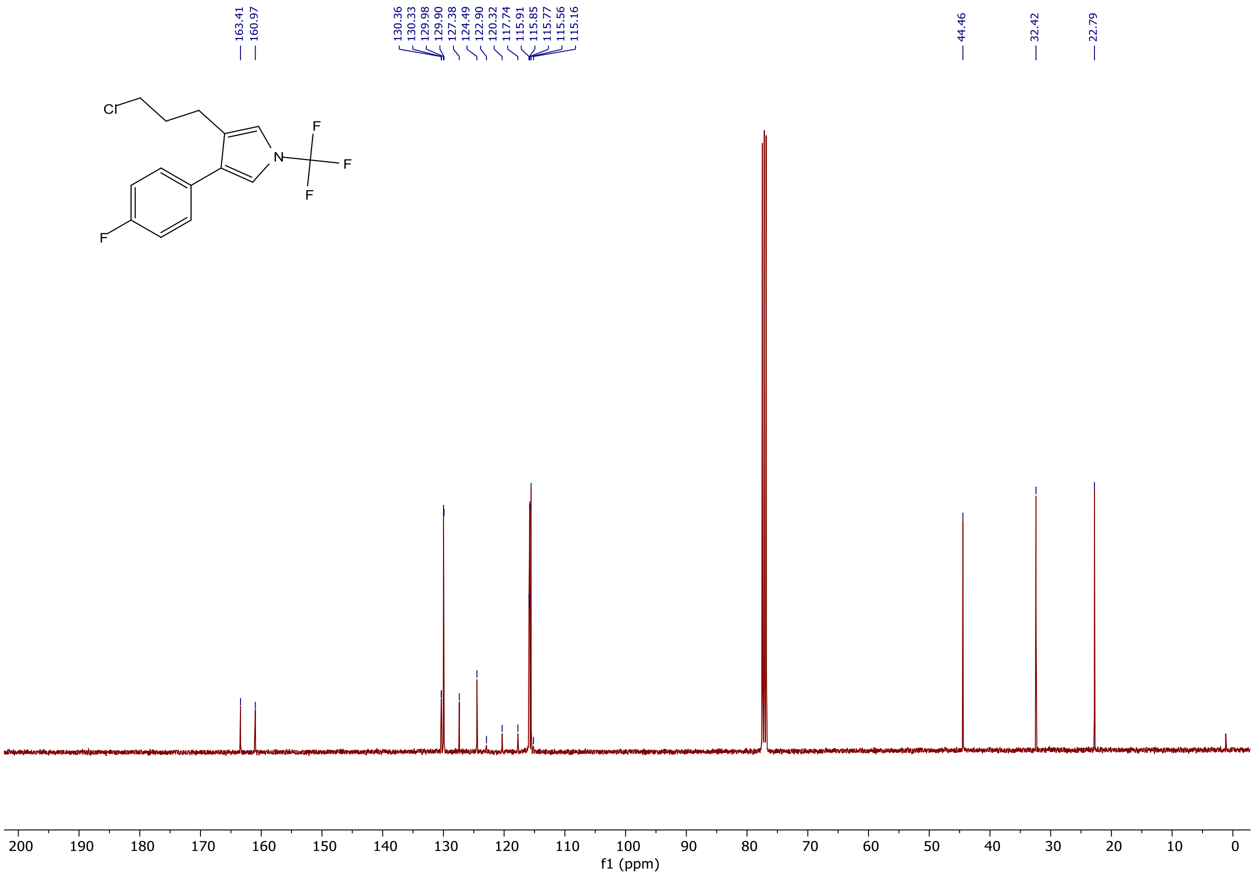

^{19}F NMR (377 MHz, $CDCl_3$) of **3n**

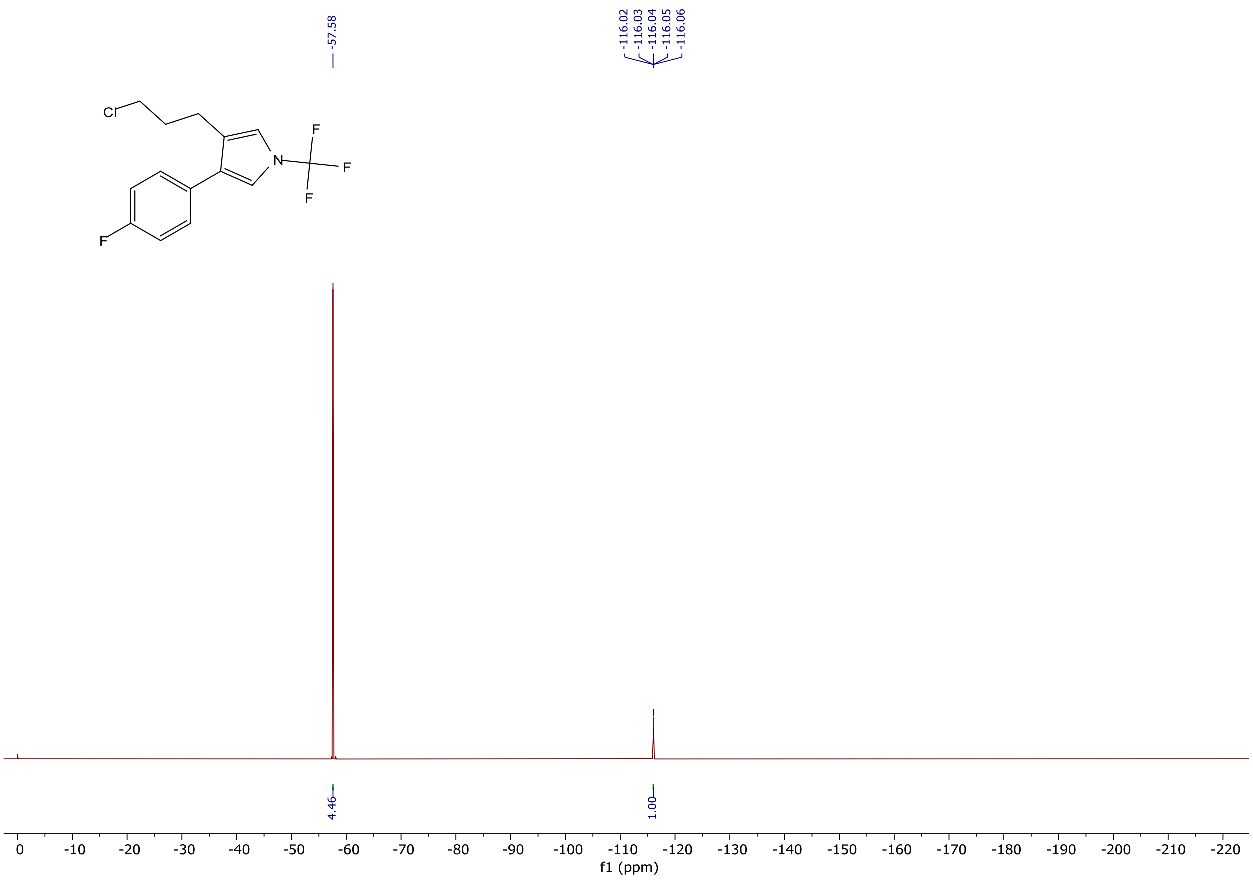

^{1}H NMR (401 MHz, $CDCl_3$) of **3n’**

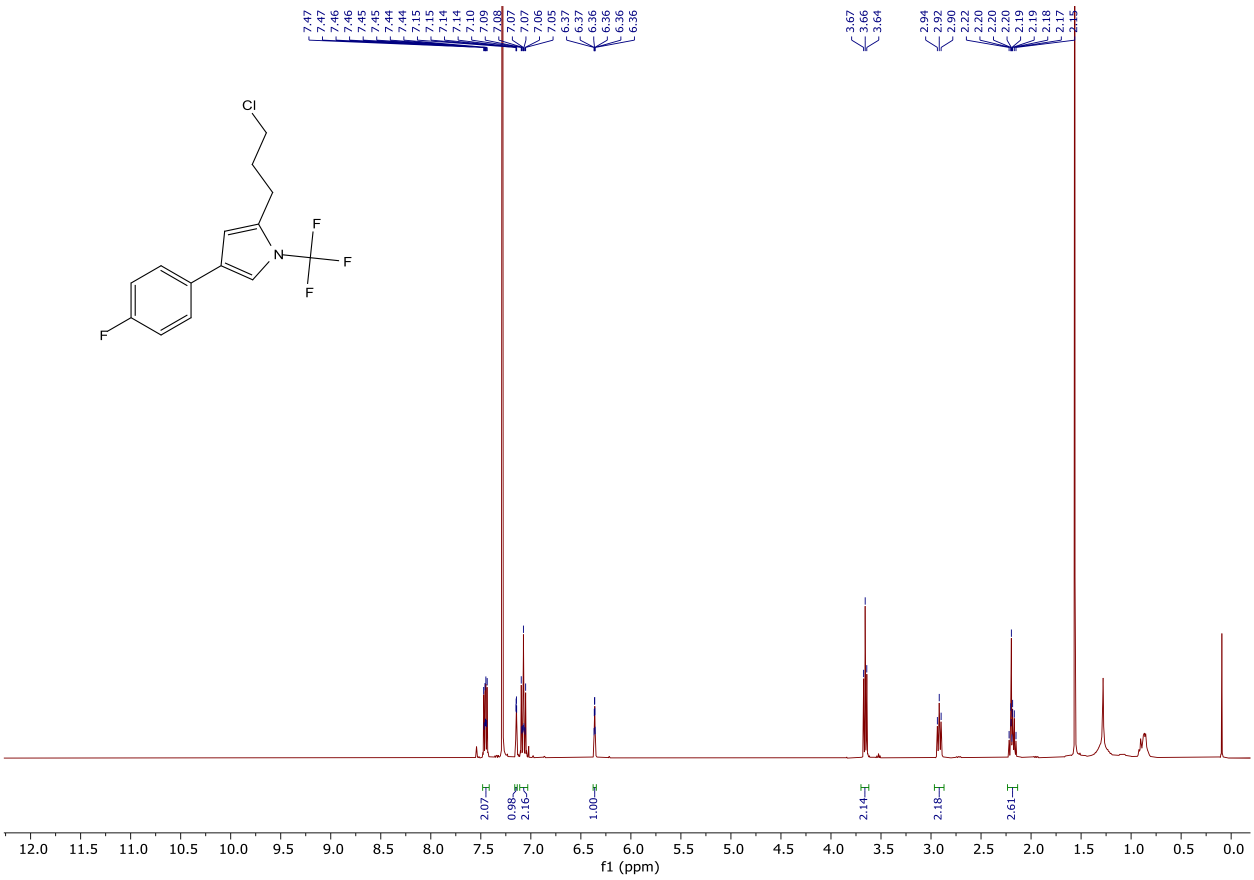

^{19}F NMR (377 MHz, $CDCl_3$) of **3n'**

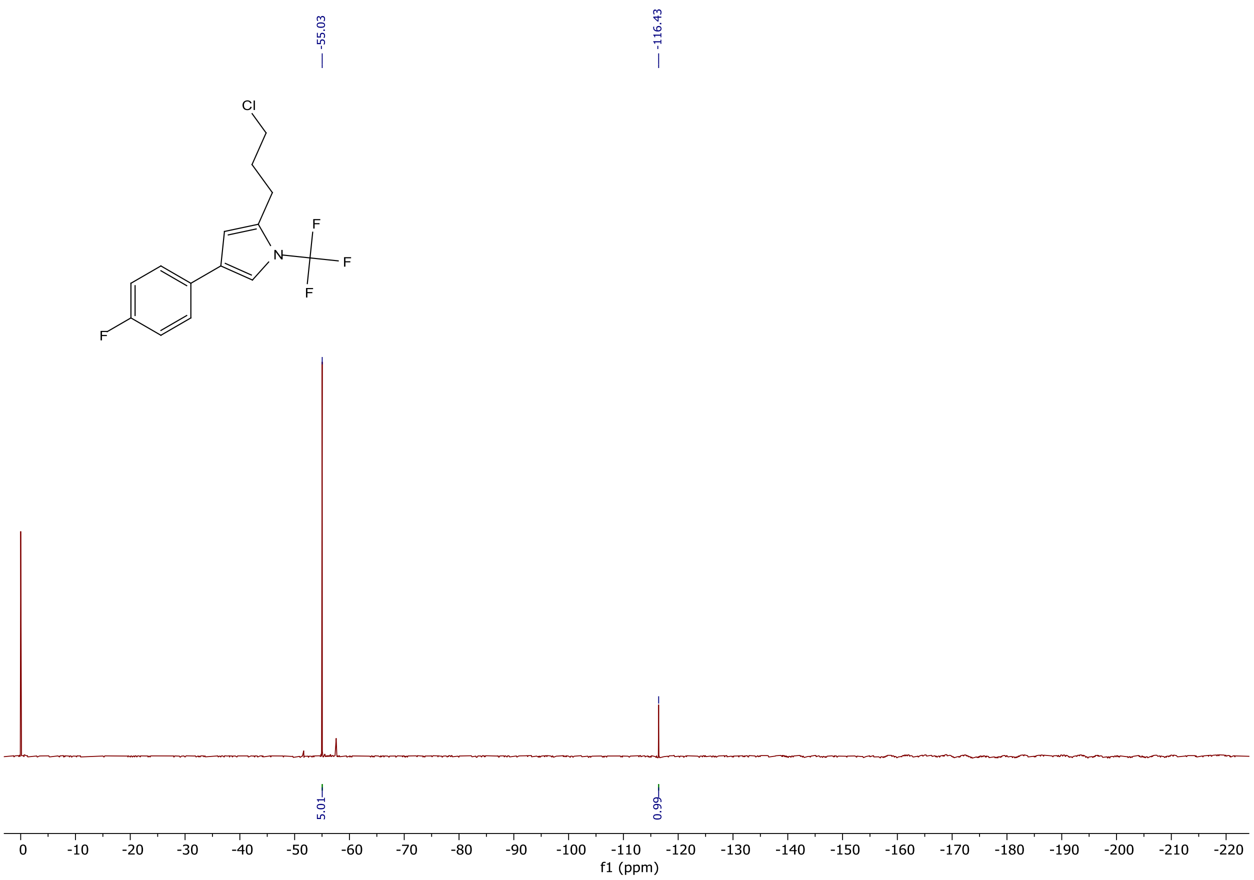

^{1}H NMR (401 MHz, $CDCl_3$) of **3o** + **3o'**

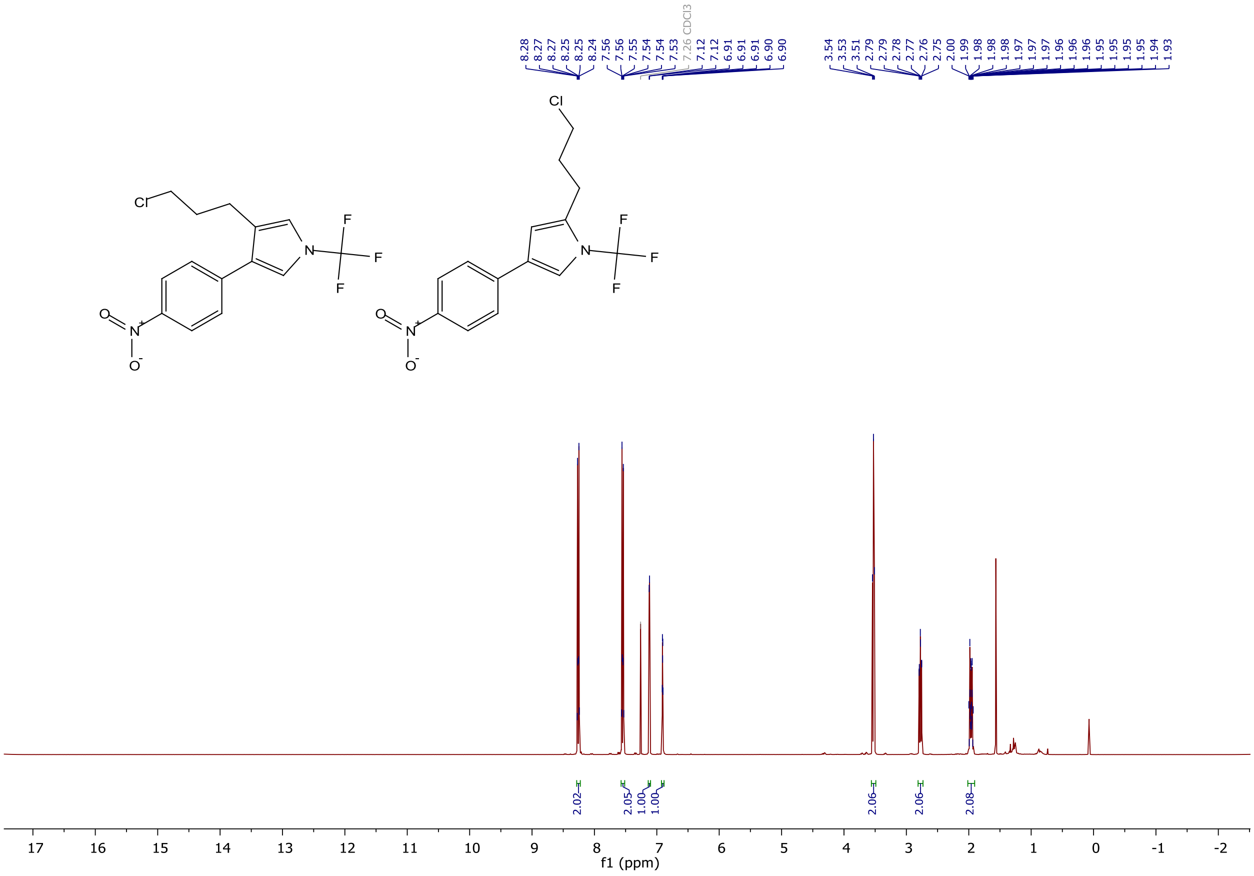

^{13}C NMR (101 MHz, $CDCl_3$) of **3o** + **3o'**

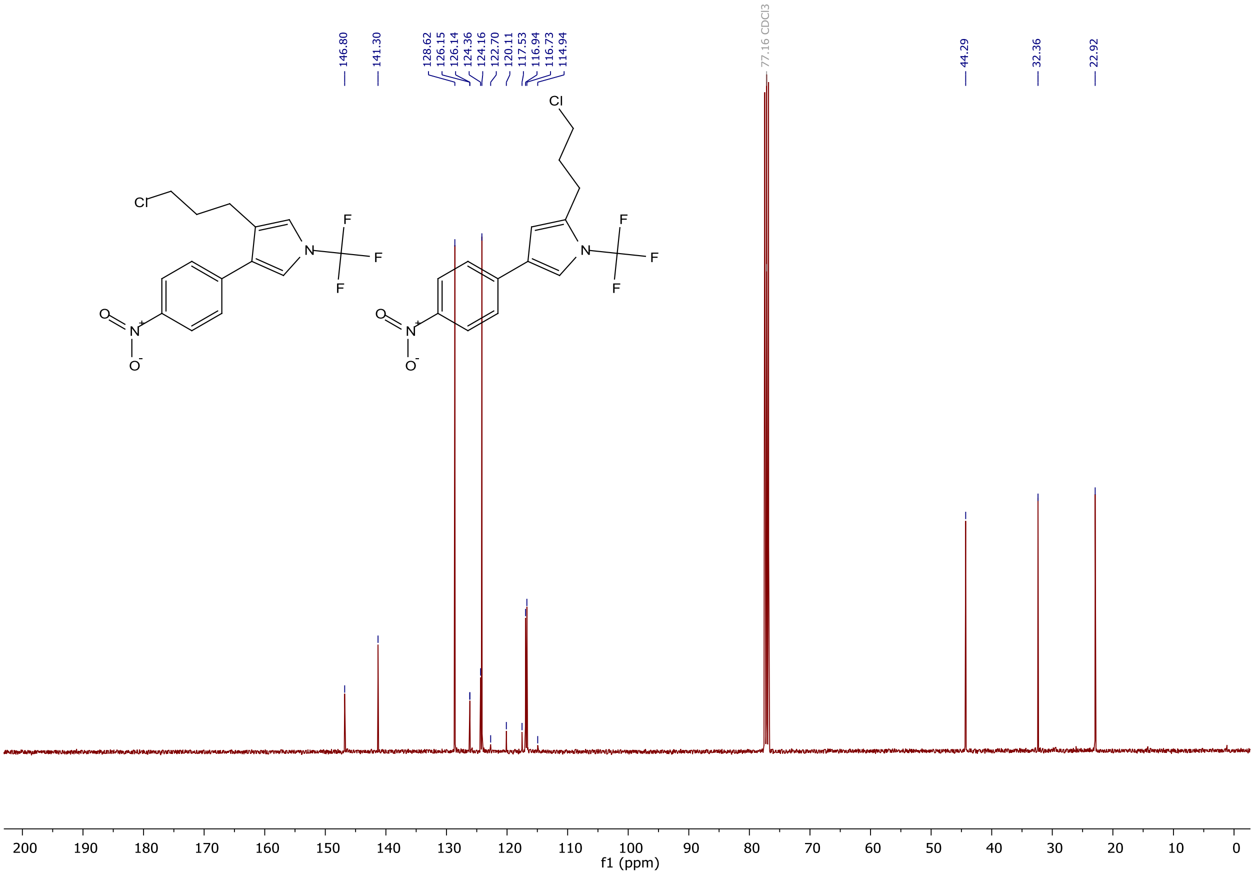

^{19}F NMR (377 MHz, $CDCl_3$) of **3o** + **3o'**

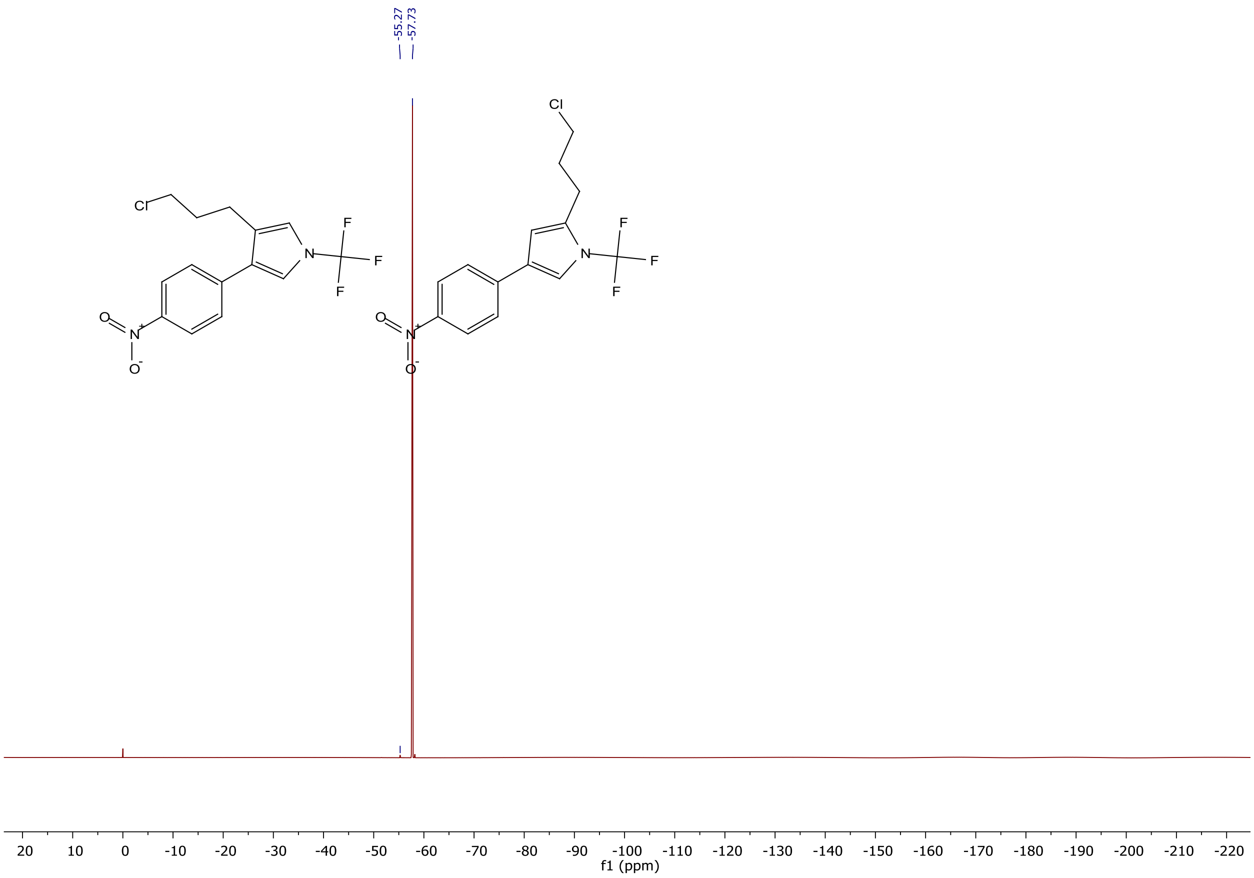

^{1}H NMR (401 MHz, $CDCl_3$) of **3p** + **3p’**

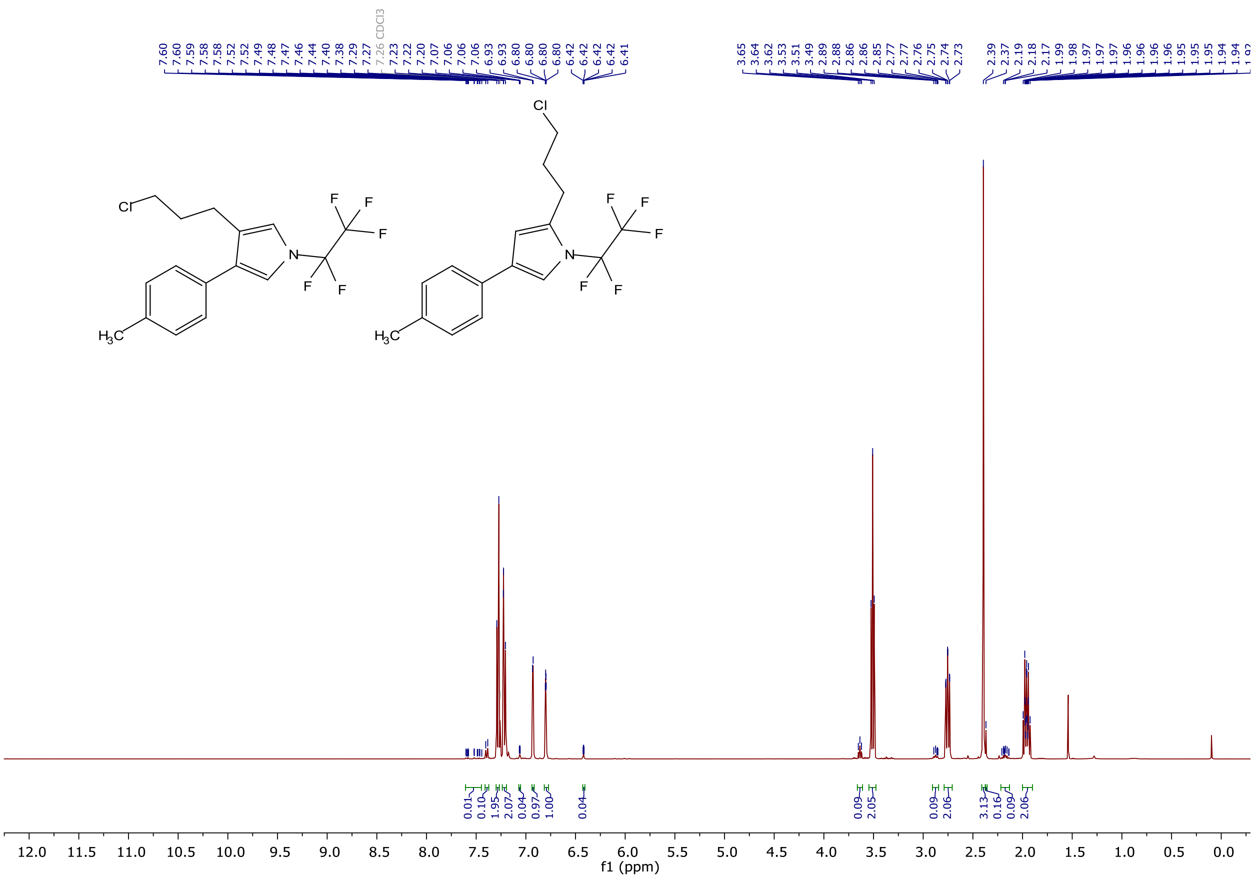

^{13}C NMR (101 MHz, $CDCl_3$) of **3p** + **3p’**

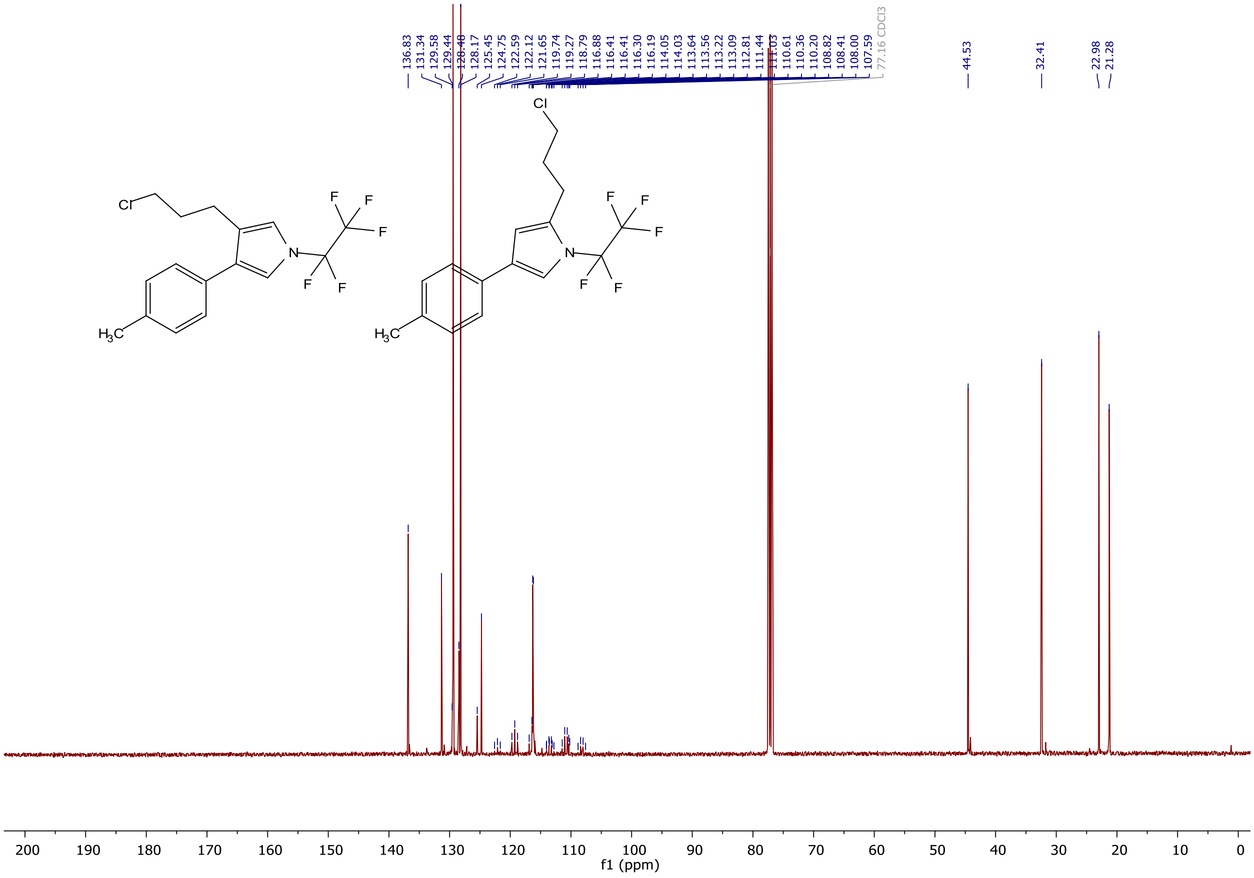

^{19}F NMR (377 MHz, $CDCl_3$) of **3p** + **3p'**

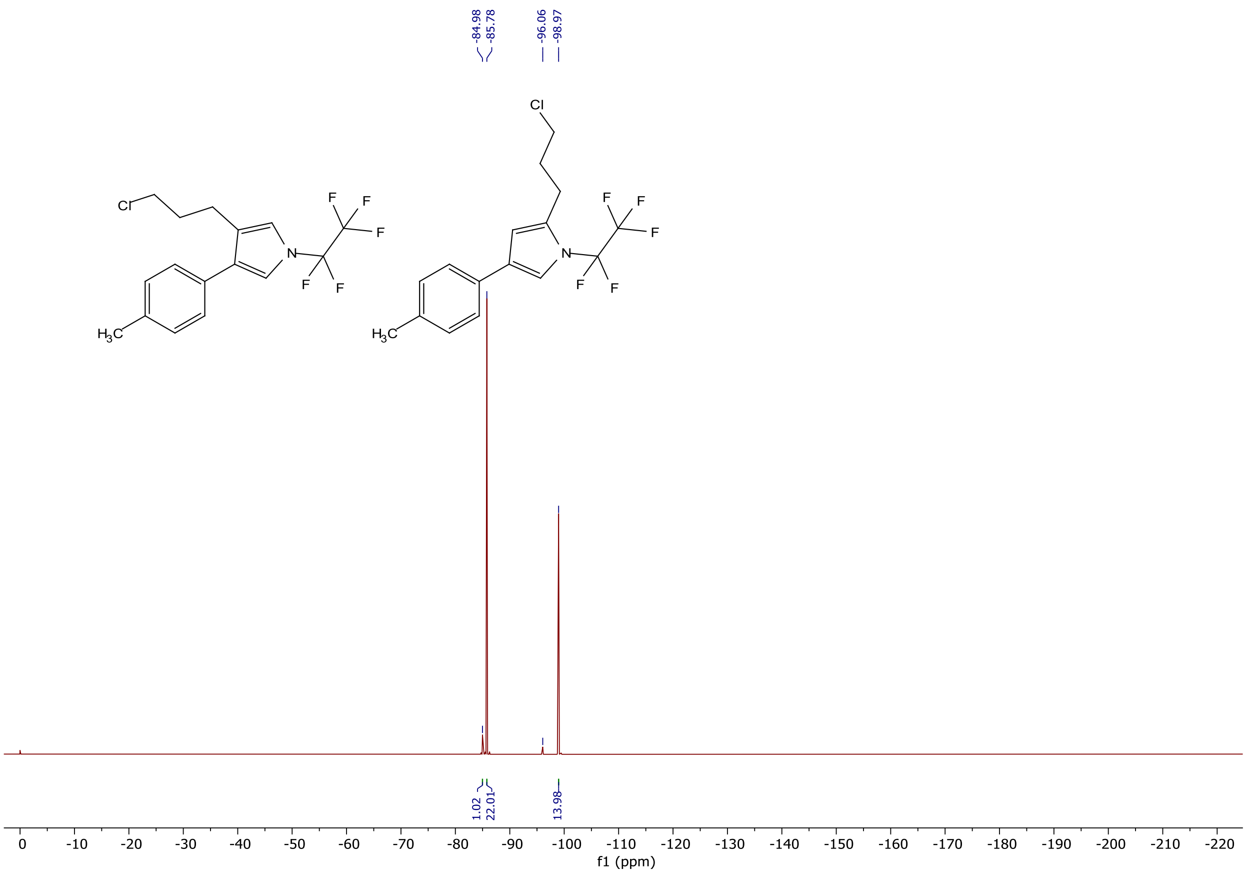

^{1}H NMR (401 MHz, $CDCl_3$) of **3q** + **3q'**

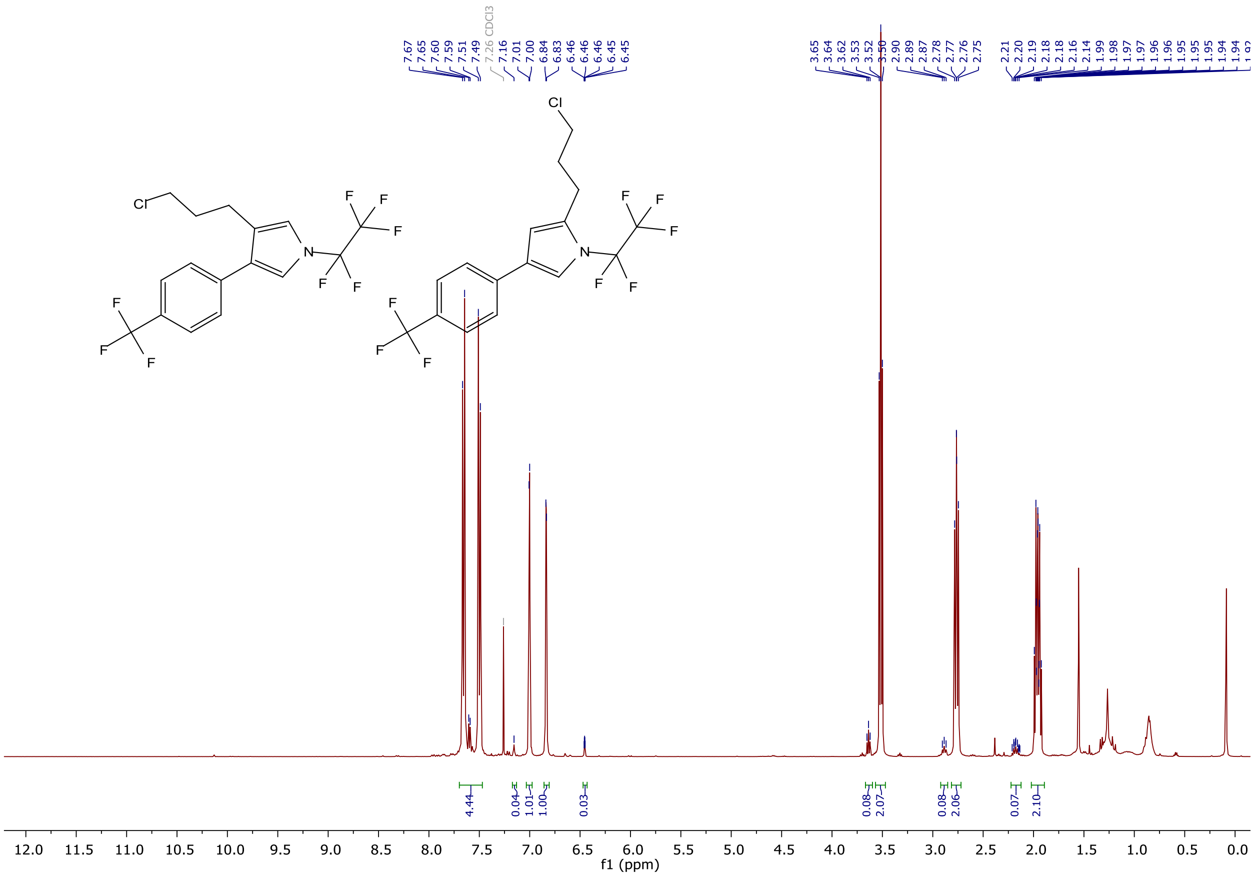

^{13}C NMR (101 MHz, $CDCl_3$) of **3q** + **3q'**

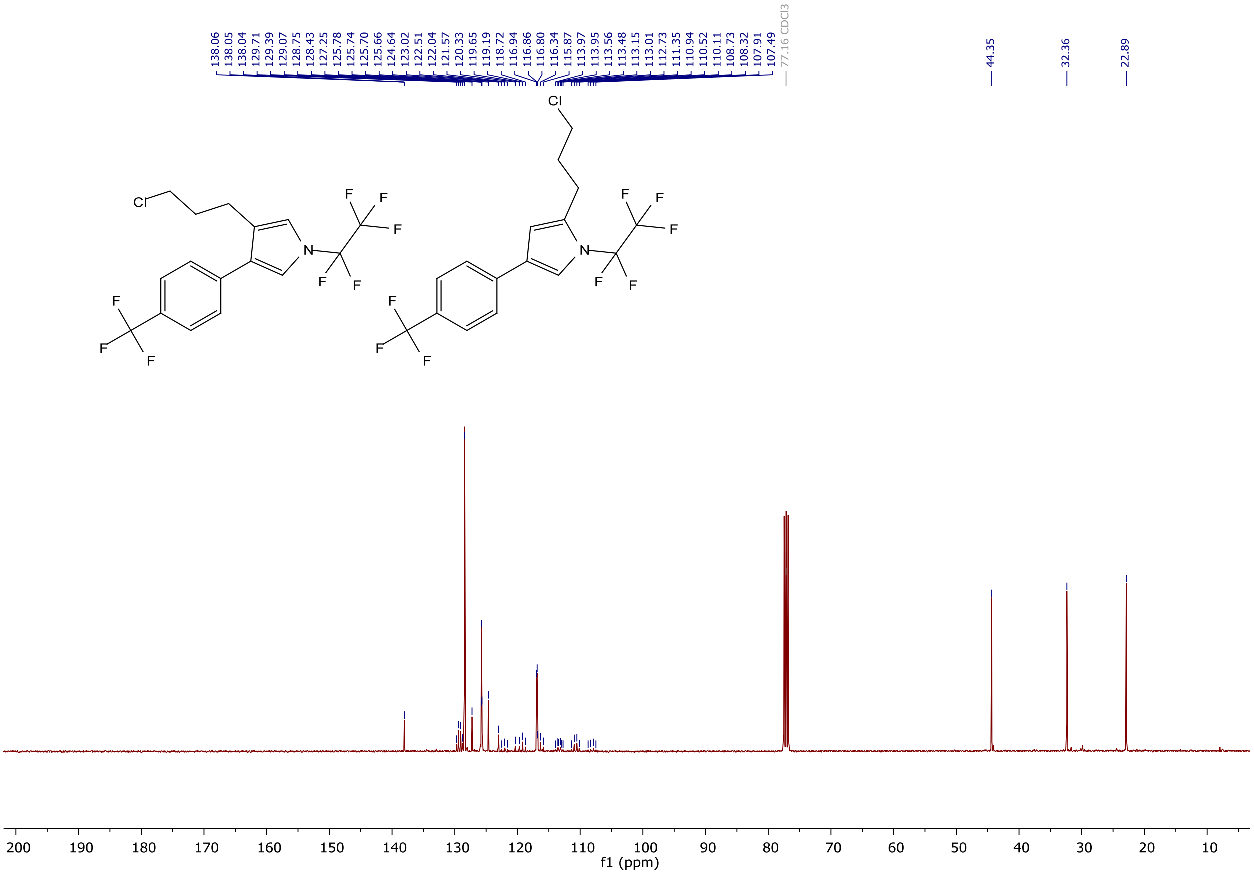

^{19}F NMR (377 MHz, $CDCl_3$) of **3q** + **3q'**

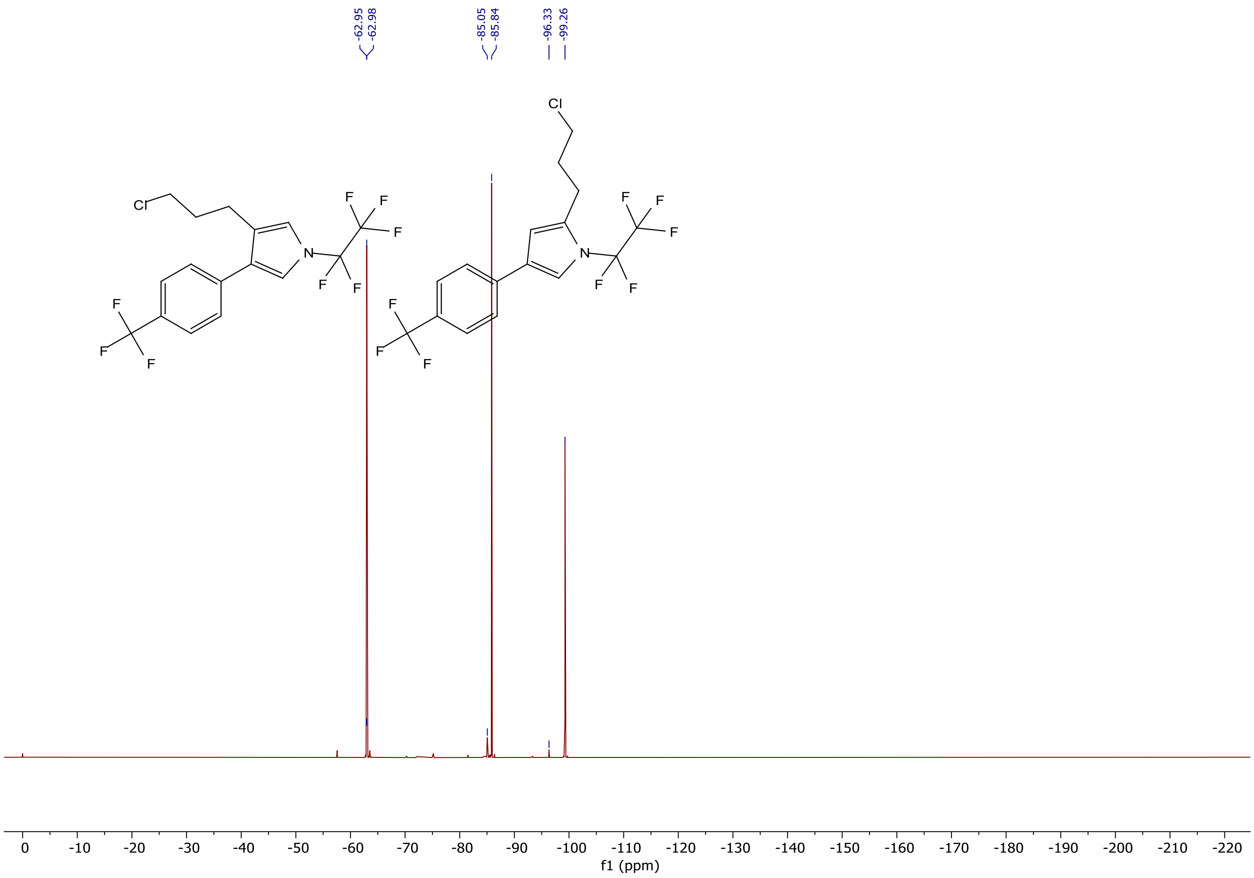

^{1}H NMR (401 MHz, $CDCl_3$) of **3r** + **3r'**

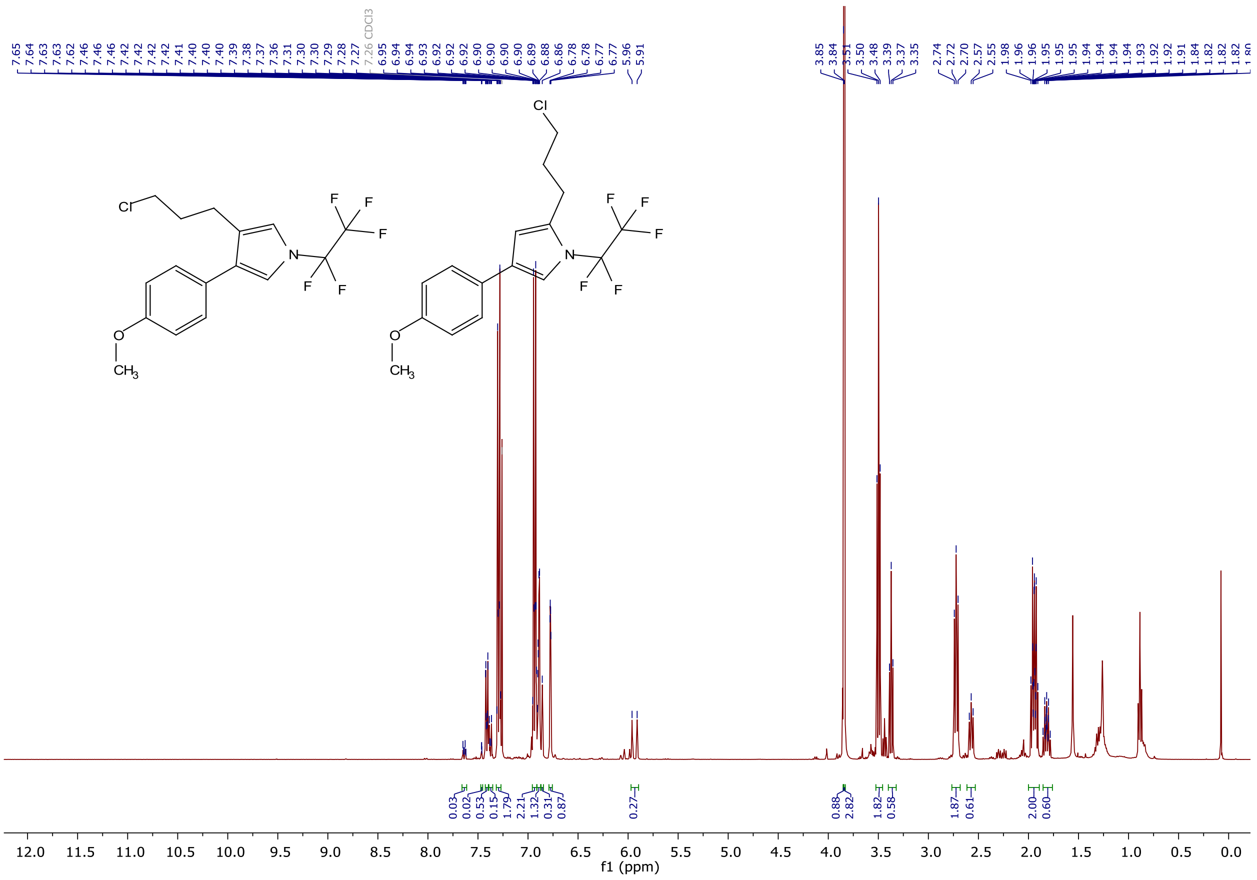

^{13}C NMR (101 MHz, $CDCl_3$) of **3r** + **3r’**

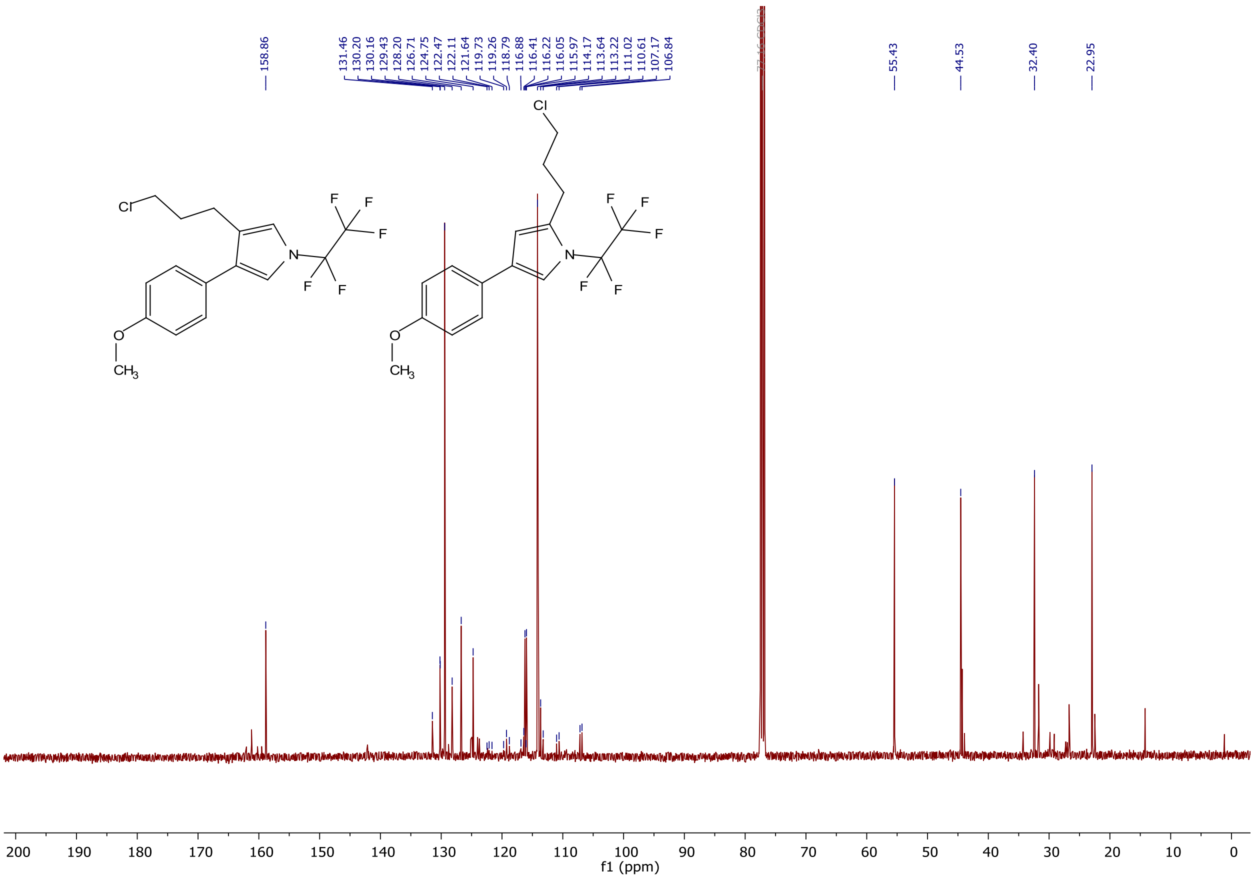

^{19}F NMR (377 MHz, $CDCl_3$) of **3r** + **3r'**

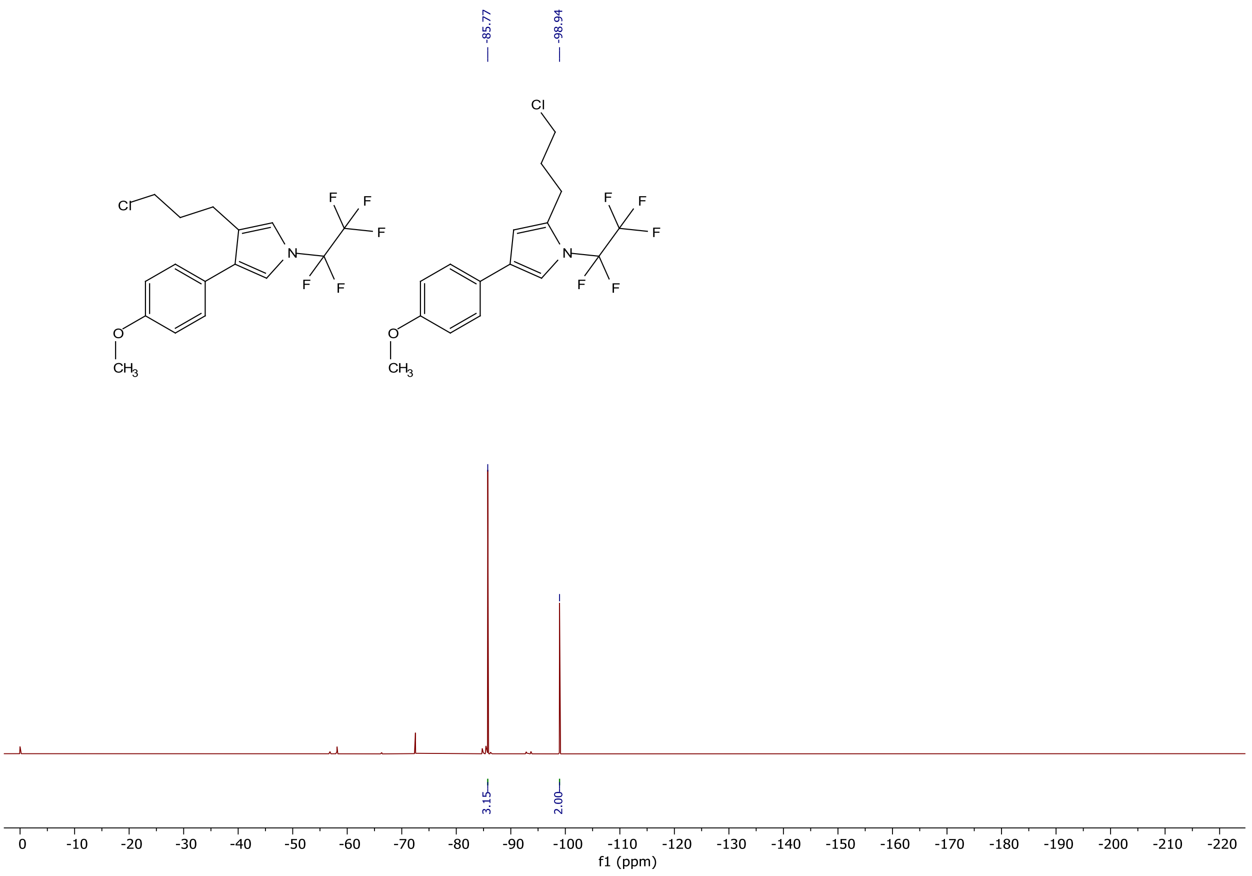

^{1}H NMR (401 MHz, $CDCl_3$) of **4**

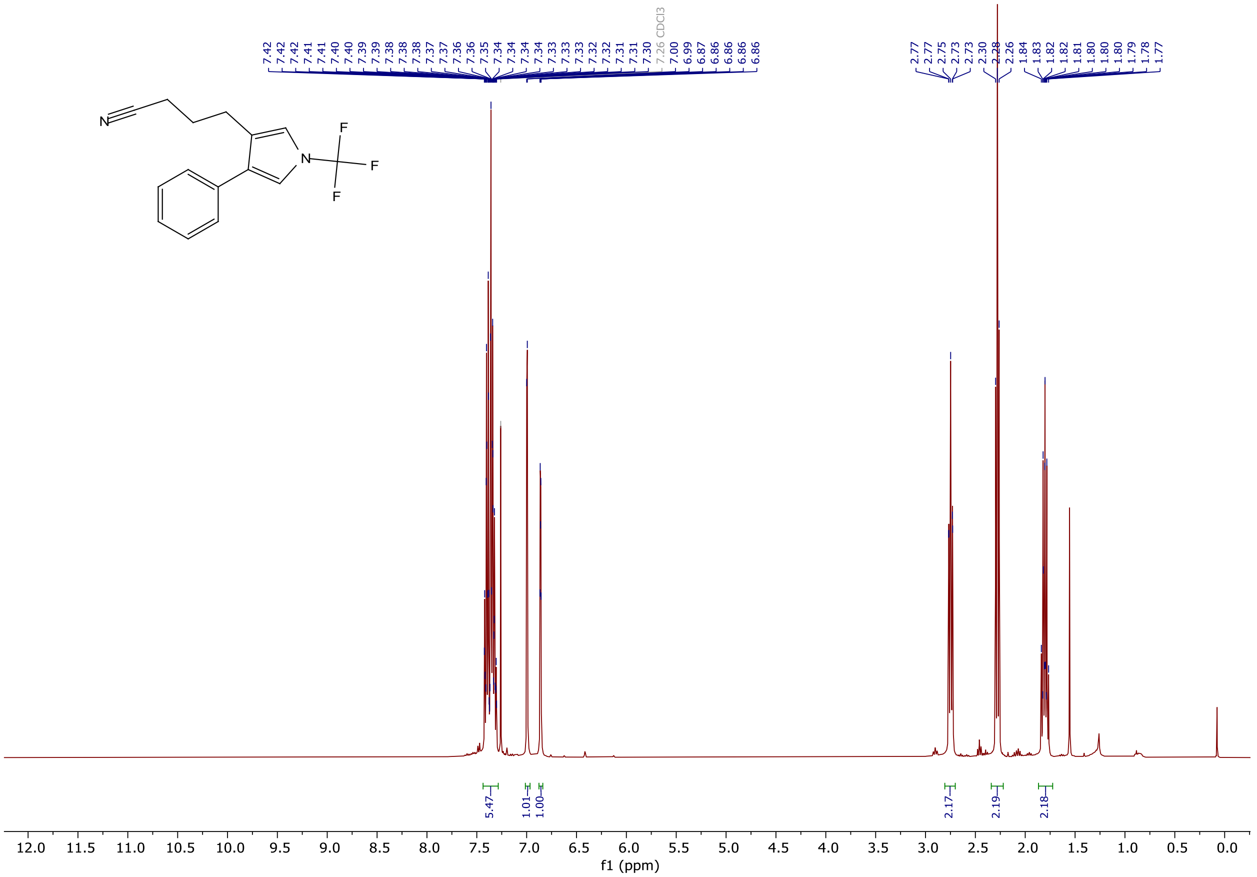

^{13}C NMR (101 MHz, $CDCl_3$) of **4**

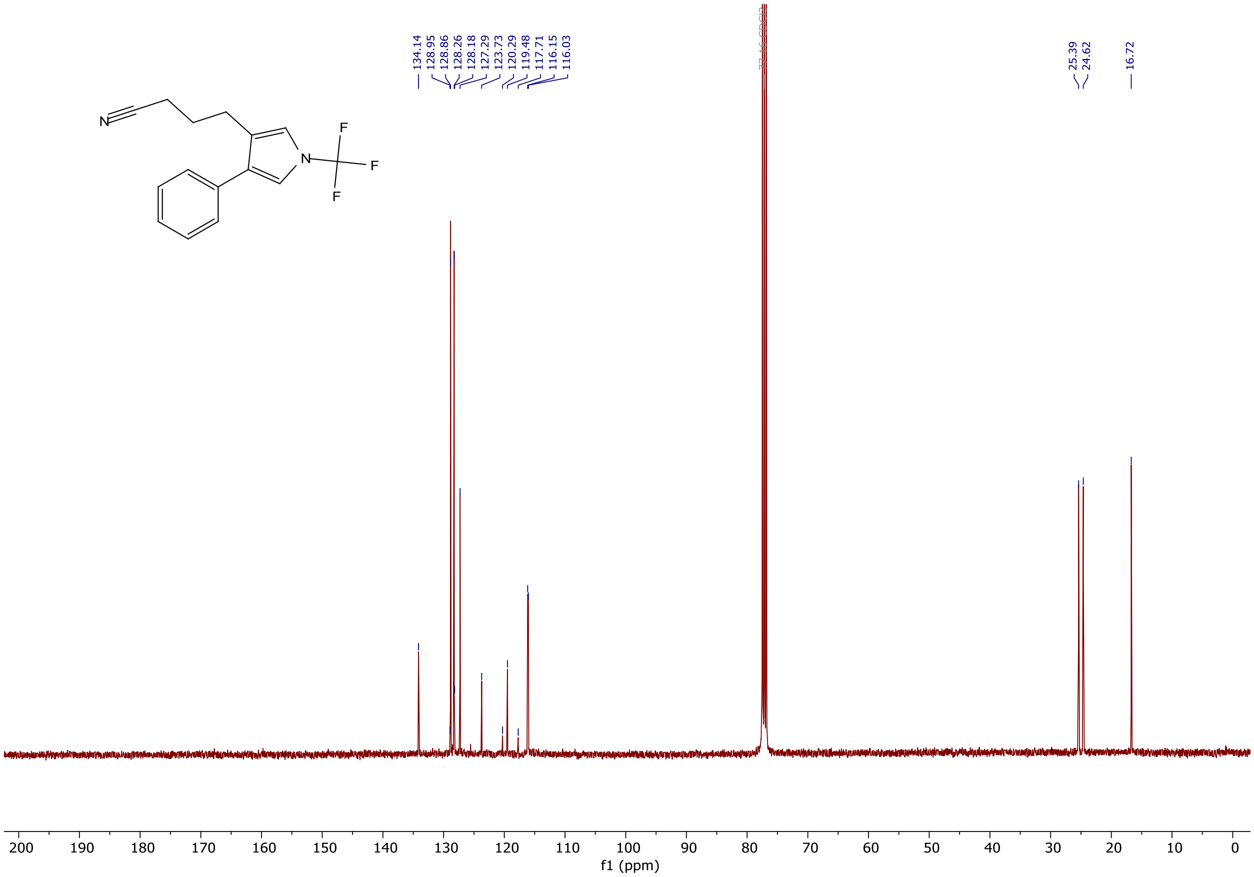

^{19}F NMR (377 MHz, $CDCl_3$) of **4**

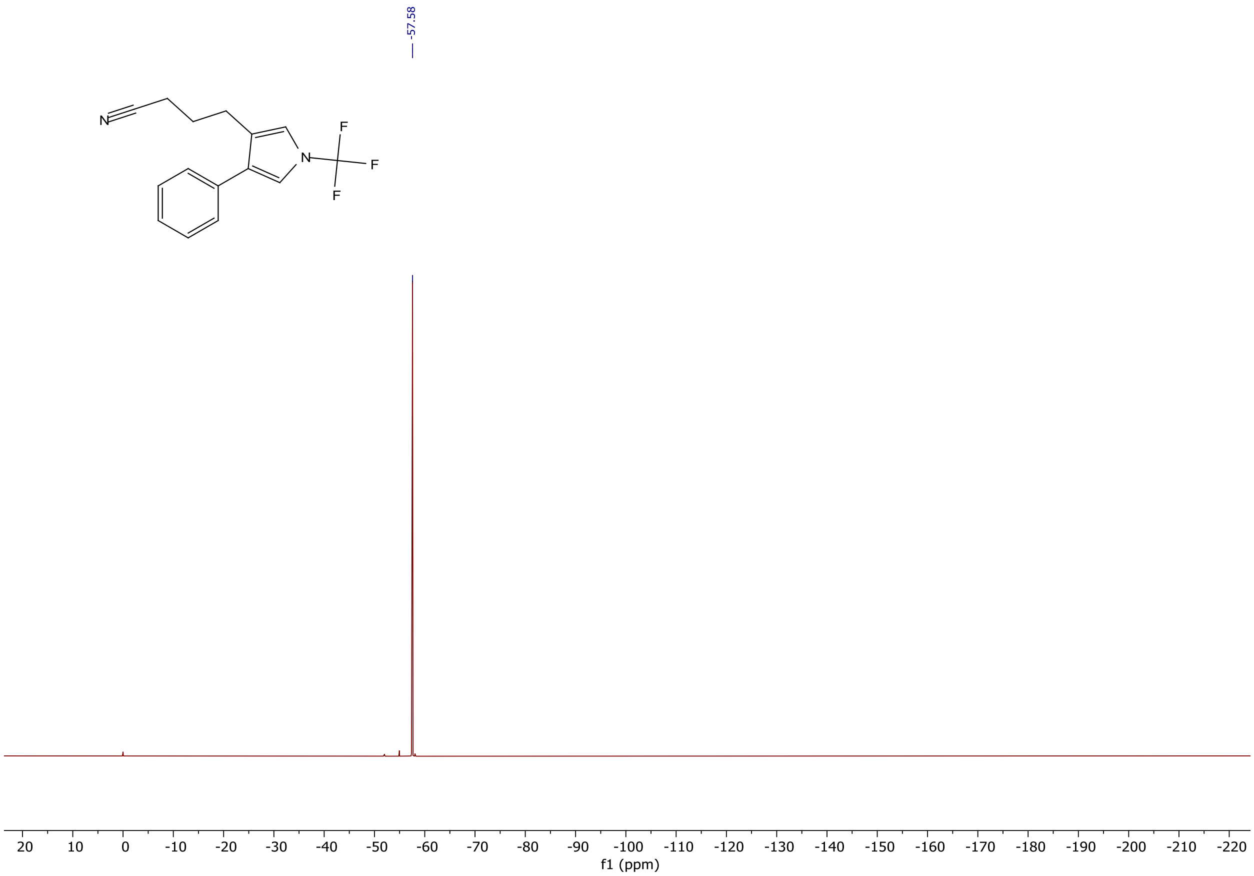

^{1}H NMR (401 MHz, $CDCl_3$) of **6**

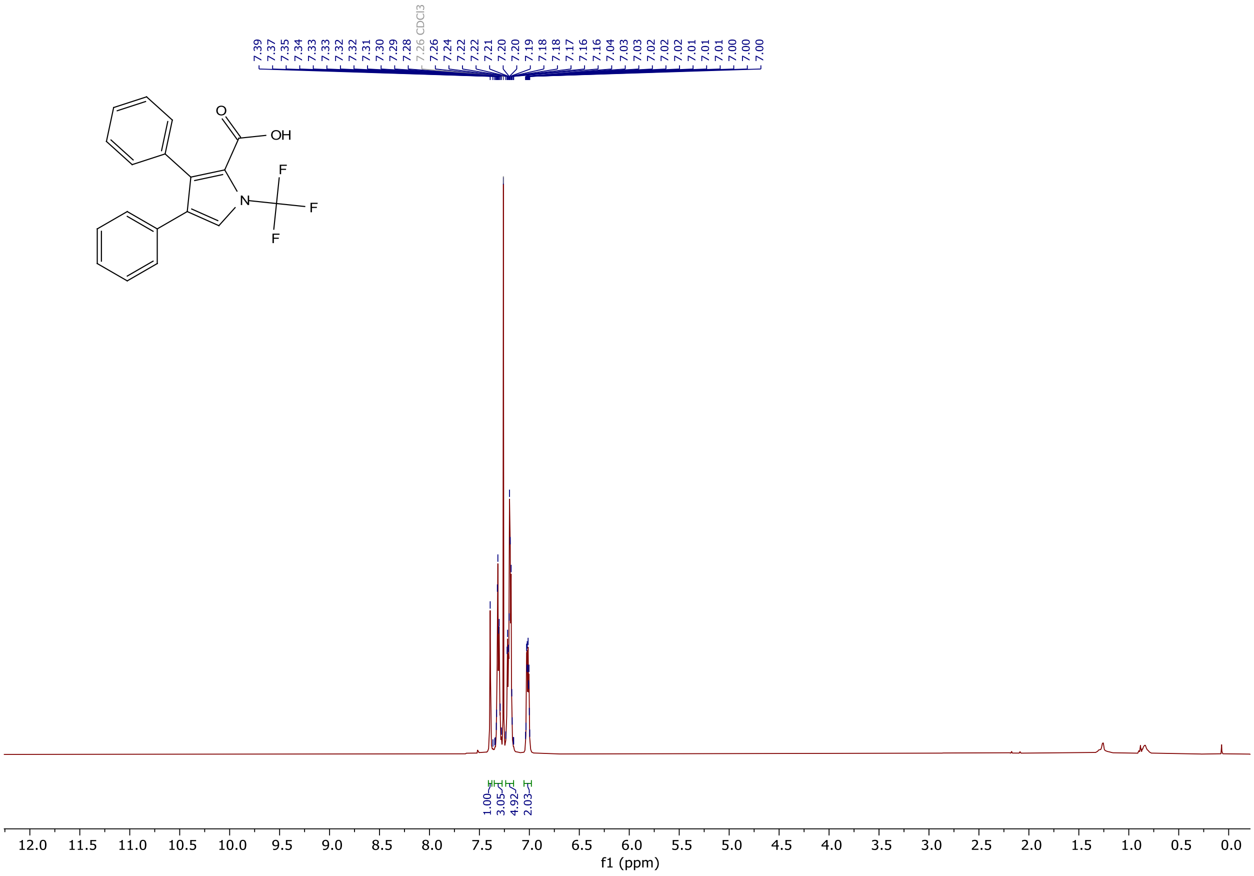

^{13}C NMR (126 MHz, $CDCl_3$) of **6**

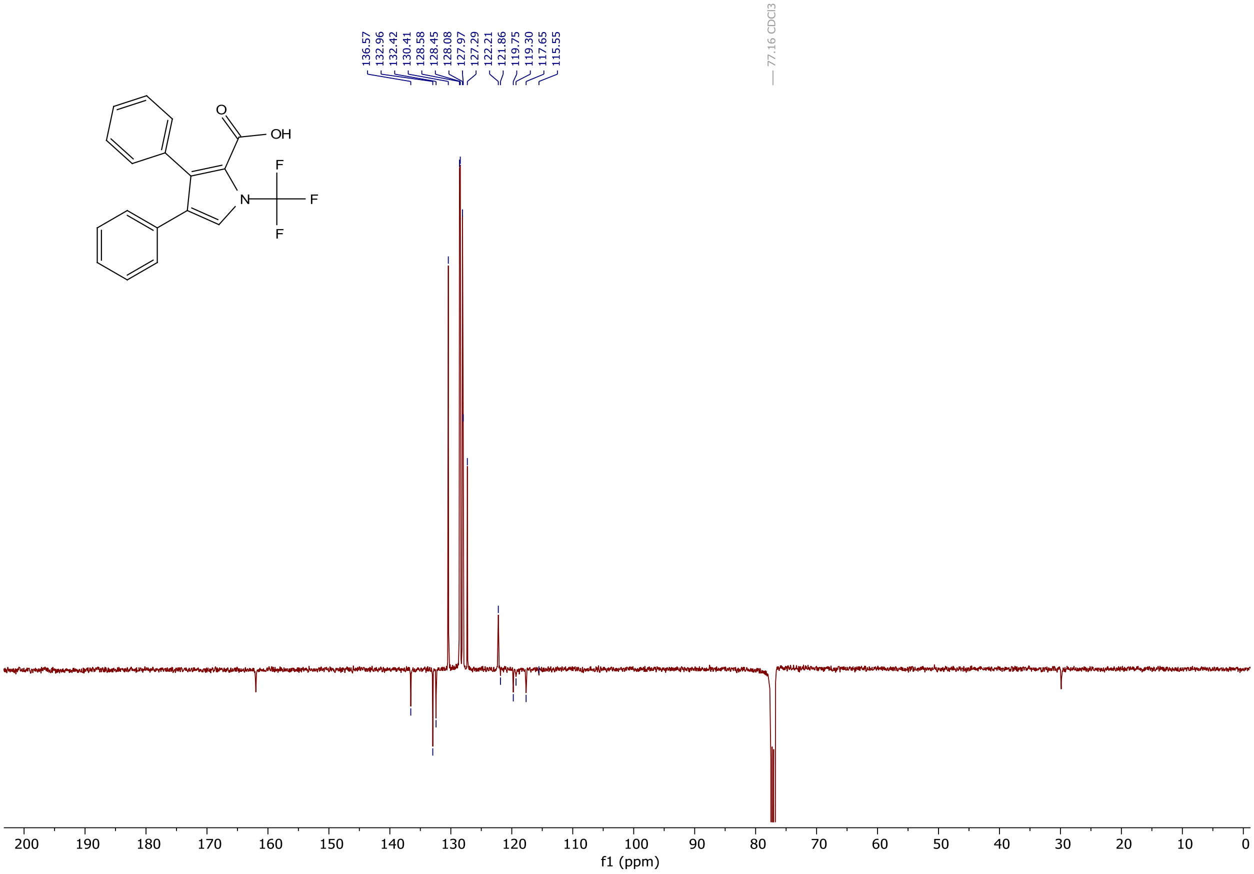

^{19}F NMR (377 MHz, $CDCl_3$) of **6**

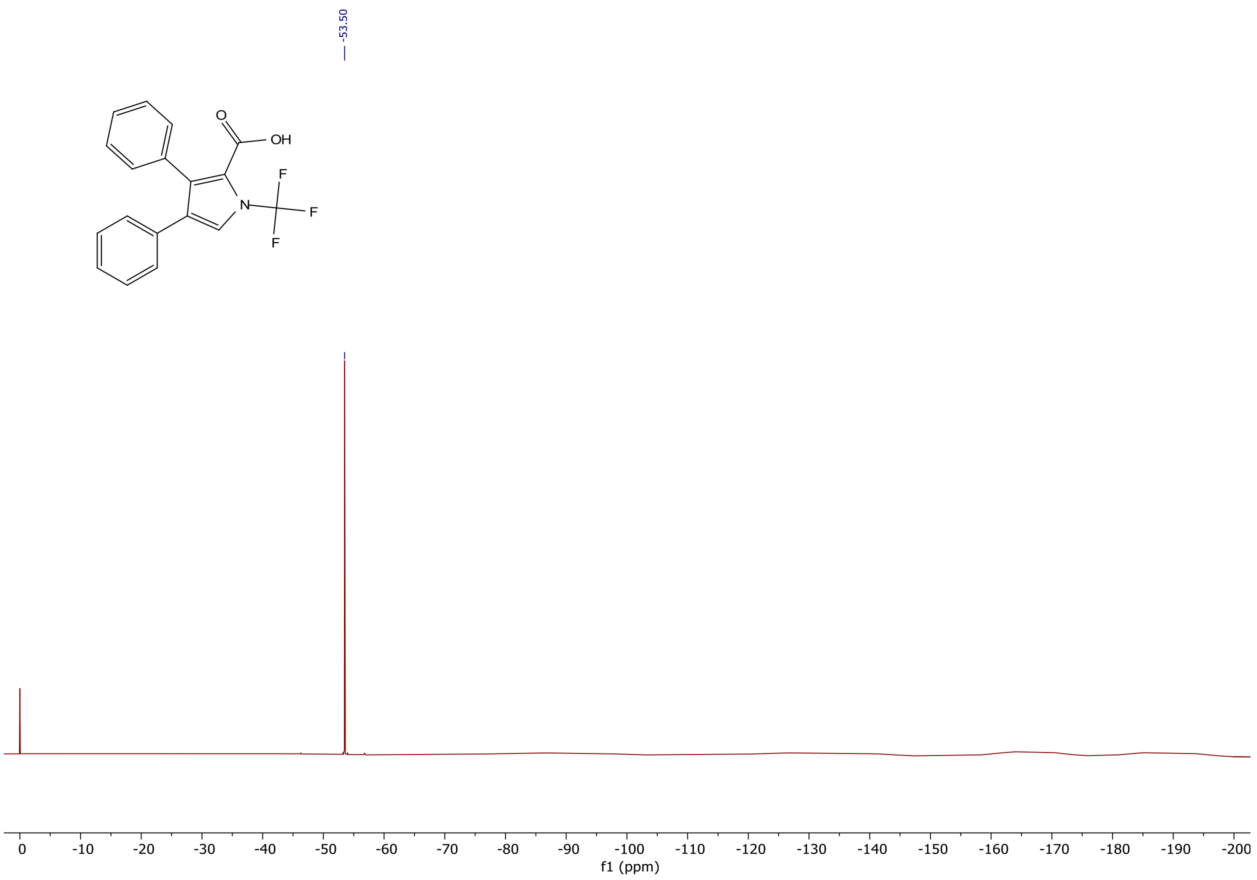

Supplement: File 1 — Experimental part. [file Beilstein_J_Org_Chem-17-504-s001.pdf]
